# Severe Acute Liver Injury After Hepatotoxic Medication Initiation in Real-World Data

**DOI:** 10.1001/jamainternmed.2024.1836

**Published:** 2024-06-24

**Authors:** Jessie Torgersen, Alyssa K. Mezochow, Craig W. Newcomb, Dena M. Carbonari, Sean Hennessy, Christopher T. Rentsch, Lesley S. Park, Janet P. Tate, Norbert Bräu, Debika Bhattacharya, Joseph K. Lim, Catherine Mezzacappa, Basile Njei, Jason A. Roy, Tamar H. Taddei, Amy C. Justice, Vincent Lo Re

**Affiliations:** 1 Division of Infectious Diseases, Department of Medicine, Perelman School of Medicine, University of Pennsylvania, Philadelphia; 2 Department of Biostatistics, Epidemiology and Informatics, Center for Real-World Effectiveness and Safety of Therapeutics, Perelman School of Medicine, University of Pennsylvania, Philadelphia; 3 Department of Non-Communicable Disease Epidemiology, London School of Hygiene & Tropical Medicine, London, United Kingdom; 4 VA Connecticut Healthcare System, US Department of Veterans Affairs, West Haven; 5 Department of Medicine, Yale School of Medicine, New Haven, Connecticut; 6 Center for Population Health Sciences, Stanford University School of Medicine, Stanford, California; 7 Division of Infectious Diseases, Department of Medicine, James J. Peters Department of Veterans Affairs Medical Center, Bronx, New York; 8 Division of Infectious Diseases, Department of Medicine, Icahn School of Medicine at Mount Sinai, New York, New York; 9 Division of Infectious Diseases, Department of Medicine, VA Greater Los Angeles Healthcare System, Los Angeles, California; 10 Division of Infectious Diseases, Department of Medicine, David Geffen School of Medicine at UCLA, Los Angeles, California; 11 Department of Biostatistics, Rutgers University School of Public Health, New Brunswick, New Jersey; 12 Division of Health Policy and Management, Yale School of Public Health, New Haven, Connecticut

## Abstract

**Importance:**

Current approaches to classify the hepatotoxic potential of medications are based on cumulative case reports of acute liver injury (ALI), which do not consider the size of the exposed population. There is little evidence from real-world data (data relating to patient health status and/or the delivery of health care routinely collected from sources outside of a research setting) on incidence rates of severe ALI after initiation of medications, accounting for duration of exposure.

**Objective:**

To identify the most potentially hepatotoxic medications based on real-world incidence rates of severe ALI and to examine how these rates compare with categorization based on case reports.

**Design, Setting, and Participants:**

This series of cohort studies obtained data from the US Department of Veterans Affairs on persons without preexisting liver or biliary disease who initiated a suspected hepatotoxic medication in the outpatient setting between October 1, 2000, and September 30, 2021. Data were analyzed from June 2020 to November 2023.

**Exposures:**

Outpatient initiation of any one of 194 medications with 4 or more published reports of hepatotoxicity.

**Main Outcomes and Measures:**

Hospitalization for severe ALI, defined by either inpatient: (1) alanine aminotransferase level greater than 120 U/L plus total bilirubin level greater than 2.0 mg/dL or (2) international normalized ratio of 1.5 or higher plus total bilirubin level greater than 2.0 mg/dL recorded within the first 2 days of admission. Acute or chronic liver or biliary disease diagnosis recorded during follow-up or as a discharge diagnosis of a hospitalization for severe ALI resulted in censoring. This study calculated age- and sex-adjusted incidence rates of severe ALI and compared observed rates with hepatotoxicity categories based on cumulative published case reports.

**Results:**

The study included 7 899 888 patients across 194 medication cohorts (mean [SD] age, 64.4 [16.4] years, 7 305 558 males [92.5%], 4 354 136 individuals [55.1%] had polypharmacy). Incidence rates of severe ALI ranged from 0 events per 10 000 person-years (candesartan, minocycline) to 86.4 events per 10 000 person-years (stavudine). Seven medications (stavudine, erlotinib, lenalidomide or thalidomide, chlorpromazine, metronidazole, prochlorperazine, and isoniazid) exhibited rates of 10.0 or more events per 10 000 person-years, and 10 (moxifloxacin, azathioprine, levofloxacin, clarithromycin, ketoconazole, fluconazole, captopril, amoxicillin-clavulanate, sulfamethoxazole-trimethoprim, and ciprofloxacin) had rates between 5.0 and 9.9 events per 10 000 person-years. Of these 17 medications with the highest observed rates of severe ALI, 11 (64%) were not included in the highest hepatotoxicity category when based on case reports.

**Conclusions and Relevance:**

In this study, incidence rates of severe ALI using real-world data identified the most potentially hepatotoxic medications and can serve as a tool to investigate hepatotoxicity safety signals obtained from case reports. Case report counts did not accurately reflect the observed rates of severe ALI after medication initiation.

## Key Points

**Question:**

What are the most hepatotoxic medications based on real-world rates of severe acute liver injury and how do these rates compare with hepatotoxicity categorization based on published case reports?

**Findings:**

This series of cohort studies among 7 899 888 persons without liver or biliary disease who initiated any of 194 suspected hepatotoxic medications in the outpatient setting from 2000 to 2021 found that 17 medications had rates of severe acute liver injury at 5.0 or more events per 10 000 person-years, representing the most potentially hepatotoxic medications; 11 medications (64%) were not included in the highest hepatotoxicity category by case reports.

**Meaning:**

Because case reports of medication hepatotoxicity do not consider the number of persons exposed, safety signals derived from case reports should be investigated using epidemiologic data.

## Teaser

This cohort study identifies the most potentially hepatotoxic medications based on clinical incidence rates of hospitalizations for severe acute liver injury (ALI) and examines how these rates compare with the hepatotoxicity categorization of severe ALI using published case reports.

## Introduction

Drug-induced acute liver injury (ALI) is the most common cause of acute liver failure in the US and Europe^1,2,3^ and historically was a frequent reason for withdrawal of approved drugs from 1975 to 2007.^4^ Despite its clinical importance, no systematic approach to classify hepatotoxicity exists, to our knowledge. Researchers have used the number of published case reports of medication hepatotoxicity listed on the US National Institutes of Health LiverTox website^5^ to create categories of likelihood for medications to cause severe ALI (category A [well-known cause], ≥50 cases; category B [highly likely], 12-49 cases; category C [probable], 4-11 cases; and category D [possible], 1-3 cases).^6^ However, cases of drug-induced ALI are frequently underreported.^7,8^ Moreover, categorizing hepatotoxic drugs using case reports does not consider the number of individuals exposed and may not accurately reflect incidence of severe ALI.

Assessing incidence rates of drug-induced ALI following medication initiation within real-world data (data relating to patient health status and/or the delivery of health care routinely collected from sources outside of a research setting) offers an independent means of categorizing the potential hepatotoxicity of medications. Because drug-induced ALI is challenging to confirm in clinical practice, remains a diagnosis of exclusion, and is susceptible to misclassification,^7^ researchers could evaluate severe ALI based on laboratory measures of substantial hepatic injury after systematically excluding non–drug-related causes. This approach provides a more objective screen for hepatotoxicity. Cohorts of initiators of suspected hepatotoxic drugs can be created, with follow-up censored at incident non–drug-related liver or biliary disease diagnoses to avoid capturing events unrelated to medication exposure. Rates of severe ALI could be measured in large cohorts, allowing investigation of hepatotoxicity safety signals from post-marketing adverse event reports. Yet, real-world evidence on rates of severe ALI following initiation of suspected hepatotoxic medications remains unknown.

This study evaluated rates of hospitalization for severe ALI after outpatient initiation of suspected hepatotoxic medications. Drugs with at least 4 published reports of hepatotoxicity were considered suspected hepatotoxins to focus analyses on the most commonly implicated products.^6^ To increase the likelihood that severe ALI events were medication related, rates were assessed after censoring for liver diseases, biliary diseases, or other conditions precipitating findings of ALI. Finally, we assessed whether observed rates of severe ALI reflected existing categories of hepatotoxicity based on the number of published case reports.

## Methods

### Study Design and Data Source

This study conducted cohort studies of initiators of suspected hepatotoxic medications within the US Department of Veterans Affairs (VA) using data between October 1, 1999, and September 30, 2021. The VA comprises more than 1200 points of care nationwide, including hospitals and outpatient clinics. The VA electronic health record data include demographic characteristics, outpatient and hospital diagnoses, laboratory results, and dispensed drugs.^9^ Race and ethnicity were not reported in this study because they should not alter the rate of severe ALI. We followed the Strengthening the Reporting of Observational Studies in Epidemiology (STROBE) reporting guideline.^10^ This study was approved by the institutional review boards of the VA Connecticut Health System, Yale University, and the University of Pennsylvania and was granted a waiver of informed consent as authorized by 45 CRF §46.116(d).

### Study Patients

Patients were eligible for inclusion in a medication initiator cohort if they had (1) a dispensed outpatient fill for any of 220 suspected hepatotoxic medications (defined by ≥4 published reports of hepatotoxicity^6^ [eTable 1 in Supplement 1]) between October 1, 2000, and September 30, 2021; and (2) 365 days or longer prior to the dispensed fill without a prior fill for that medication. We focused on outpatient medication initiation to increase the likelihood that severe ALI events were drug related; inclusion of medication initiators during hospitalization would increase the potential that severe ALI might be due to hospital-related events. A medication was not evaluated if it was (1) not dispensed in the VA during the study period; (2) administered via injection or intravenous route (excluding chemotherapy and hormone therapy); (3) used for alcohol use disorder or liver disease treatment; or (4) an anticoagulant, which would prevent identification of ALI-related coagulopathy. We did not evaluate topical, otic, ophthalmic, subdermal, rectal, or vaginal medications.

The index date was the date the medication was initially dispensed in the outpatient setting. For patients who were dispensed multiple courses, only the first was evaluated. Patients could be included in more than 1 cohort if dispensed multiple suspected hepatotoxic medications during the study period. The 365 days prior to the index date represented the baseline period. Patients were excluded if, during baseline, they had (1) hospitalization for severe ALI (to avoid including prevalent outcomes); (2) anticoagulant dispensed; (3) preexisting liver or biliary disease (to increase the likelihood that severe ALI events were medication related), defined by hepatitis B infection (positive hepatitis B surface antigen, e antigen, or DNA test result), hepatitis C infection (positive hepatitis C RNA or genotype test result), or liver or biliary disease diagnosis (eTable 2 in Supplement 1); or (4) a condition that might precipitate findings consistent with severe ALI (eTable 3 in Supplement 1). To minimize potential for inclusion of persons with undiagnosed liver or biliary disease, we further excluded those who, during baseline, had 2 alanine aminotransferase (ALT) results at 40 U/L or higher (to convert to μkat/L, multiply by 0.0167) at least 6 months apart (validated to identify persons with metabolic dysfunction–associated steatotic liver disease in the VA health care system^11^), 1 ALT level greater than 100 U/L, or 1 alkaline phosphatase result with a level greater than 172 mg/dL (1.5 times the upper limit of normal [ULN], 115 mg/dL) (to convert to μkat/L, multiply by 0.0167).

Follow-up began on the index date and continued until study end point (defined in the first paragraph of the next section), medication discontinuation (no further fills within 30 days after last day’s supply), anticoagulant dispensing, incident liver or biliary disease or other condition precipitating findings of ALI, 12 months after index date (because drug-induced ALI typically develops within 12 months of initiation^12^), last VA contact, or September 30, 2021, whichever occurred first.

### Main Study Outcome

The primary outcome was hospitalization for severe ALI, defined by meeting either of the following definitions within the first 2 days of admission: (1) ALT level greater than 120 U/L (3 times ULN, 40 U/L) plus total bilirubin level greater than 2.0 mg/dL (to convert to mmol/L, multiply by 17.104, 2 times ULN, 1.0 mg/dL) (definition 1); or (2) international normalized ratio (INR) of 1.5 or higher plus total bilirubin level greater than 2.0 mg/dL (definition 2). Both definitions represent severe ALI and have been used by the US Food and Drug Administration’s Sentinel System to assess clinically significant drug-induced ALI in the postmarketing period.^13^ Definition 1 represents Hy’s Law biochemical criteria,^14^ which identifies hepatocellular injury severe enough to interfere with bilirubin excretion and predisposes a high mortality risk.^15,16^ Definition 2 identifies hepatic dysfunction that might present in an advanced stage of acute liver failure, in which liver aminotransferases might not be high enough to meet definition 1.^13^ We evaluated severe ALI within the first 2 days of admission to avoid ascertaining outcomes that developed as a result of hospitalization. Outcomes were classified on the admission date. We assessed hospitalizations for severe ALI because such events represent ALI at the severe end of the spectrum, minimizing potential for misclassification bias. To increase the likelihood that severe ALI events were drug related, patients with a discharge diagnosis of liver or biliary disease or other condition that precipitates findings consistent with severe ALI (eTables 2 and 3 in Supplement 1) were censored as nonevents on the admission date.

To estimate the frequency that severe ALI events were medication related, we randomly sampled 75 patients who had an event. Two hepatologists independently reviewed the hospital records of these patients and assessed whether a medication possibly caused or contributed to the severe ALI. Among the 75 patients, 57 (76.0%; 95% CI, 64.7%-85.1%) had a hospitalization for severe ALI that was deemed medication related. Details are given in the eMethods in Supplement 1.

### Covariates

Baseline data included date and route of medication administration and days’ supply; age; sex; body mass index (BMI; calculated as weight in kilograms divided by height in meters squared); diabetes and hyperlipidemia diagnoses (eTable 4 in Supplement 1); medication count at initiation date; and INR, total bilirubin, ALT, and alkaline phosphatase results. Medication count was evaluated because of the observed linear association between the number of dispensed medications and drug interactions.^17^ Medication count was defined by the number of unique drugs dispensed 90 days or less prior to the index date; patients were classified as having polypharmacy if dispensed 5 or more medications.^18,19^ For co-formulated medications, each component was counted separately. Data collected during follow-up included number of days’ supply of medications; hospital ALT, INR, and total bilirubin level; anticoagulant use; liver transplant (eTable 5 in Supplement 1); and all-cause mortality within 180 days of hospitalization for severe ALI.

### Statistical Analysis

We calculated age- and sex-adjusted rates of outcomes for each medication using Poisson regression analysis. The model included age (as a continuous variable), sex, and suspected hepatotoxic drug. A test for goodness of fit confirmed the data were not overdispersed. To ensure sufficient precision to estimate rates, we included medication cohorts in the primary analysis if either (1) the 95% CI width for the rate was less than 3 times the point estimate, or (2) 10 000 or more person-years of follow-up was present.

We organized medications into groups based on observed rate of severe ALI (≥10.0 [group 1], 5.0-9.9 [group 2], 3.0-4.9 [group 3], 1.0-2.9 [group 4], and <1.0 [group 5] events per 10 000 person-years). We classified groups 1 and 2 medications as the most potentially hepatotoxic. We identified the number (percentage) of groups 1 and 2 medications that were classified as category A, B, or C by case report–based categorization.^6^

We performed several sensitivity analyses to assess the robustness of the results. First, we repeated the analysis after additionally adjusting for diabetes, hyperlipidemia, and obesity (BMI > 30), since metabolic comorbidities may alter rates of severe ALI.^20^ Second, we stratified results by sex. Third, we reran our analysis after limiting the cohorts to persons younger than 65 years, since veterans younger than 65 years are less likely to receive care outside the VA because they are typically ineligible for US Medicare.^21^ Fourth, we explored the effects of drug-drug interactions on the results. Since patients could be dispensed multiple hepatotoxic medications that might increase rates of severe ALI and misclassify assignment of medications to a group, we excluded individuals who were additionally dispensed any medication from groups 1 and 2 no more than 90 days prior to the index date and recalculated the rates. To explore whether our groupings were preserved with polypharmacy, we recalculated rates among initiators dispensed 5 or more unique medications within 90 days prior to the index date. Data were analyzed from June 2020 to November 2023 using SAS Enterprise Guide 8.2 (SAS Institute Inc).

## Results

The study included 7 899 888 patients who initiated treatment with a suspected hepatotoxic medication in the outpatient setting from 2000 to 2021 (mean [SD] age, 64.4 [16.4] years, 7 305 558 males [92.5%], and 4 354 136 individuals [55.1%] had polypharmacy).

Among the 220 medications with 4 or more published reports of hepatotoxicity, 26 could not be evaluated because they were (1) not dispensed in the VA (n = 8), (2) administered via injection or an intravenous route (n = 13), (3) used for alcohol use disorder or liver disease treatment (n = 2), or (4) anticoagulants (n = 3), leaving 194 medications for evaluation (eTable 1 in Supplement 1). For each of the 194 medication cohorts, eTable 6 in Supplement 1 provides the reasons for exclusion and final samples. The most common reason for exclusion was 365 or fewer days in the VA prior to the initial outpatient fill.

Among the 7 899 888 patients, we identified 1739 hospitalizations for severe ALI; 5 patients (0.3%) underwent liver transplant and 473 (27.2%) died within 180 days of an event. After calculating age- and sex-adjusted rates of severe ALI, 91 medications (46.9%) were not included in the primary analysis because either the 95% CI was more than 3 times the point estimate of severe ALI or there was too little person-time of follow-up (eTable 7 in Supplement 1). Three of these drugs (didanosine, nevirapine, and pyrazinamide) had rates of 10 events or more per 10 000 person-years, while 68 drugs had no observed events.

**Figure 1.**
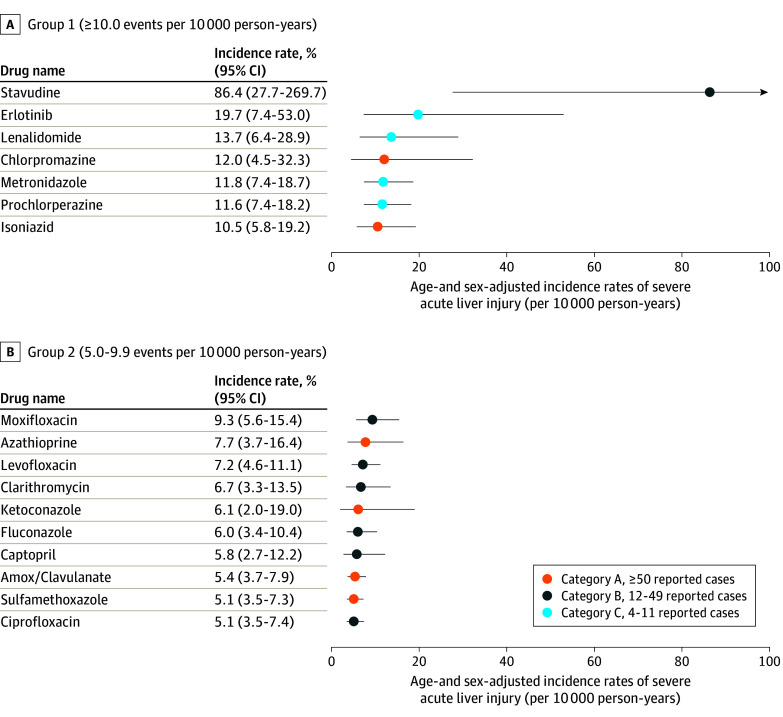
Incidence Rates of Hospitalization for Severe Acute Liver Injury (ALI) for Group 1 and 2 Medications Age- and sex-adjusted incidence rates (solid circles), reported with 95% CIs (whiskers), were assessed among patients without preexisting liver or biliary disease and after censoring for incident diagnoses of liver or biliary diseases or other conditions that might precipitate findings consistent with severe ALI. Colors represent the categories of likelihood to cause severe ALI based on the number of published case reports on the medication’s hepatotoxicity. Amox indicates amoxicillin.

**Figure 2.**
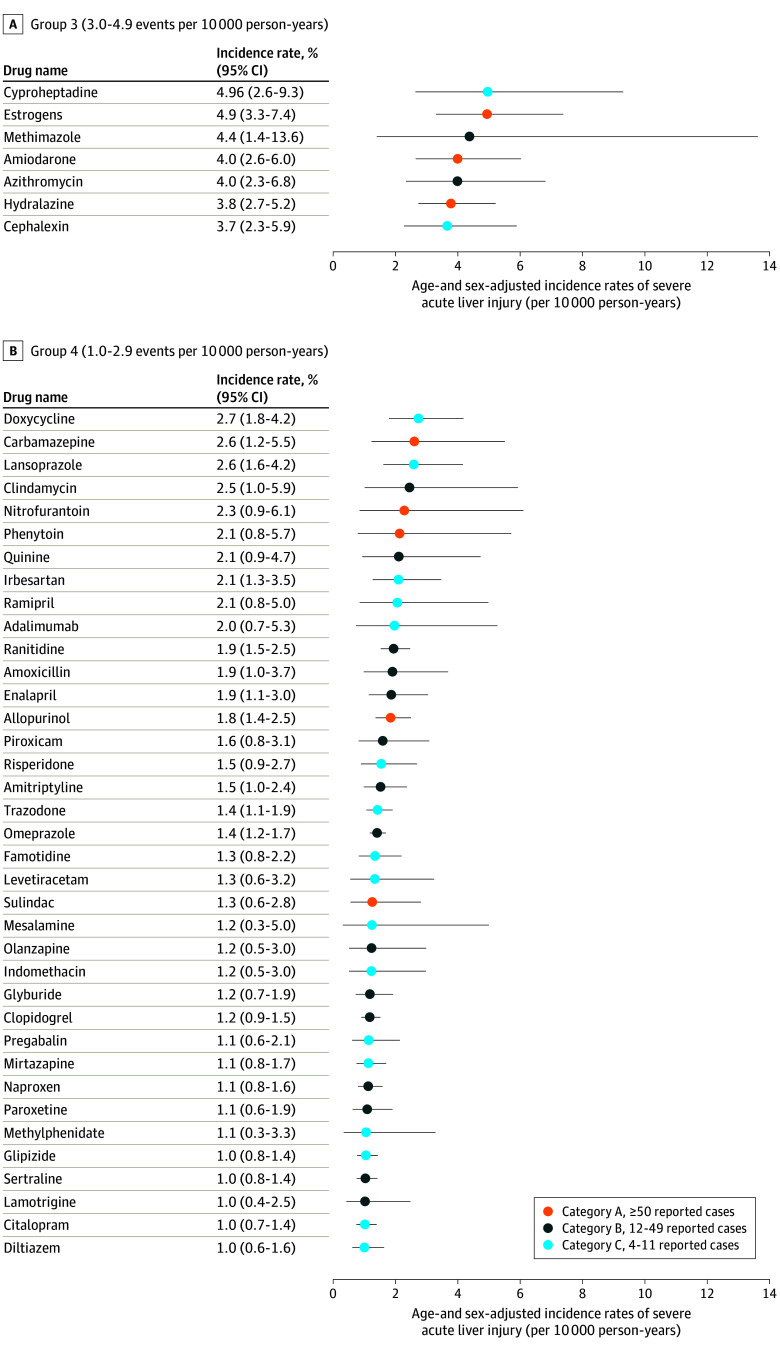
Incidence Rates of Hospitalization for Severe Acute Liver Injury (ALI) for Group 3 and 4 Medications Age- and sex-adjusted incidence rates (solid circles), reported with 95% CIs (whiskers), were assessed among patients without preexisting liver or biliary disease and after censoring for incident diagnoses of liver or biliary diseases or other conditions that might precipitate findings consistent with severe ALI. Colors represent the categories of likelihood to cause severe ALI based on the number of published case reports of the medication’s hepatotoxicity.

**Figure 3.**
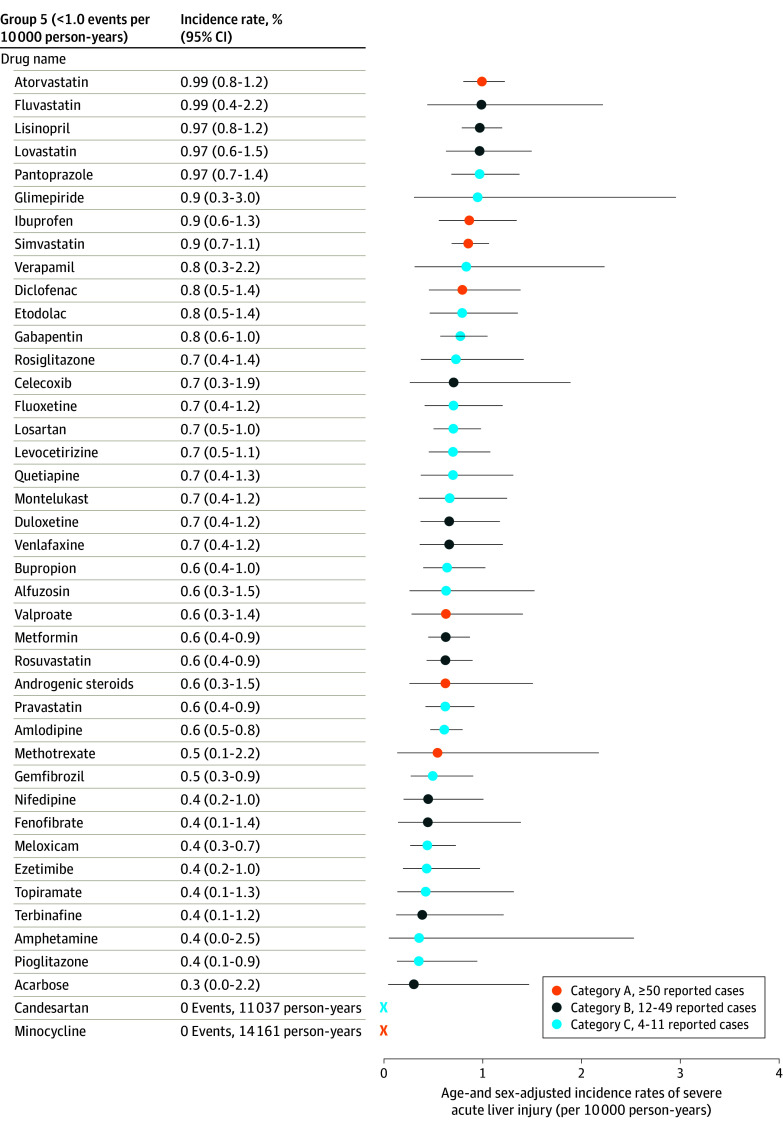
Incidence Rates of Hospitalization for Severe Acute Liver Injury (ALI) for Group 5 Medications Age- and sex-adjusted incidence rates (solid circles), reported with 95% CIs (whiskers), were assessed among patients without preexisting liver or biliary disease and after censoring for incident diagnoses of liver or biliary diseases or other conditions that might precipitate findings consistent with severe ALI. Colors represent the categories of likelihood to cause severe ALI based on numbers of published case reports of hepatotoxicity.

Figure 1 shows the 17 medications in groups 1 and 2 with the highest observed rates of severe ALI, their age- and sex-adjusted rates of severe ALI, and hepatotoxicity category based on the number of reported cases. Sample sizes and numbers of events are reported in the Table. Incidence rates of severe ALI ranged from 0 events per 10 000 person-years (candesartan, minocycline) to 86.4 events per 10 000 person-years (stavudine). Seven medications (stavudine, erlotinib, lenalidomide or thalidomide, chlorpromazine, metronidazole, prochlorperazine, and isoniazid) exhibited rates of 10.0 or more events per 10 000 person-years, and 10 (moxifloxacin, azathioprine, levofloxacin, clarithromycin, ketoconazole, fluconazole, captopril, amoxicillin-clavulanate, sulfamethoxazole-trimethoprim, and ciprofloxacin) had rates between 5.0 and 9.9 events per 10 000 person-years. Despite these medications having the highest rates of severe ALI, 11 medications (64%) were classified in the less hepatotoxic categories B or C based on case reports. Eleven medications (64%) in groups 1 and 2 were antimicrobials. Rates of severe ALI, sample sizes, and number of events for the medications in group 3 (n = 7), group 4 (n = 37), and group 5 (n = 42) are shown in Figure 2 and Figure 3 and in eTable 8 in Supplement 1. Among the group 3, 4, and 5 medications, 3 (43%), 5 (14%), and 8 (19%), respectively, were classified as category A (most hepatotoxic) by case reports.

**Table.** Age- and Sex-Adjusted Incidence Rates of Hospitalization for Severe Acute Liver Injury for Group 1 and 2 Medications^a^

Medication	Hepatotoxicity category^b^	No. of initiators	Age, median (IQR), y	Male, No. (%)	Female, No. (%)	No. of person-years	No. of events	Adjusted incidence rate (95% CI), events per 10 000 person-years^c^
Group 1 (≥10.0 events per 10 000 person-years)								
Stavudine^d^	B	750	48.5 (42.0-54.8)	724 (96.5)	26 (3.5)	306	3	86.4 (27.7-269.7)
Erlotinib	C	4356	71.3 (64.4-78.7)	4225 (97.0)	131 (3.0)	1133	4	19.7 (7.4-53.0)
Lenalidomide or thalidomide	C	8191	71.8 (65.4-78.2)	7975 (97.4)	216 (2.6)	2860	7	13.7 (6.4-28.9)
Chlorpromazine	A	17 449	60.4 (49.7-69.7)	16 253 (93.1)	1196 (6.9)	2542	4	12.0 (4.5-32.3)
Metronidazole^d^	C	423 666	57.6 (43.6-68.2)	309 774 (73.1)	113 892 (26.9)	12 340	19	11.8 (7.4-18.7)
Prochlorperazine	C	167 779	63.5 (51.9-72.2)	145 045 (86.5)	22 734 (13.5)	11 999	20	11.6 (7.4-18.2)
Isoniazid^d^	A	20 476	58.6 (47.4-69.2)	18 719 (91.4)	1757 (8.6)	7642	11	10.5 (5.8-19.2)
Group 2 (5.0-9.9 events per 10 000 person-years)								
Moxifloxacin^d^	B	376 367	63.9 (56.1-73.7)	346 663 (92.1)	29 704 (7.9)	11 141	16	9.3 (5.6-15.4)
Azathioprine or mercaptopurine	A	16 033	62.6 (50.3-71.3)	14 188 (88.5)	1845 (11.5)	6305	7	7.7 (3.7-16.4)
Levofloxacin or ofloxacin^d^	B	580 210	65.9 (55.8-73.9)	537 103 (92.6)	43 107 (7.4)	18 799	21	7.2 (4.6-11.1)
Clarithromycin^d^	B	210 356	61.8 (52.0-70.9)	190 190 (90.4)	20 166 (9.6)	8169	8	6.7 (3.3-13.5)
Ketoconazole^d^	A	29 976	59.0 (45.5-70.5)	27 763 (92.6)	2213 (7.4)	2980	3	6.1 (2.0-19.0)
Fluconazole^d^	B	287 646	55.6 (40.7-67.7)	172 110 (59.8)	115 536 (40.2)	16 008	13	6.0 (3.4-10.4)
Captopril	B	18 863	70.5 (60.4-78.3)	18 378 (97.4)	485 (2.6)	6859	7	5.8 (2.7-12.2)
Amoxicillin with clavulanate^d^	A	1 235 143	60.6 (49.2-69.9)	1 114 900 (90.3)	120 243 (9.7)	38 233	29	5.4 (3.7-7.9)
Sulfamethoxazole with trimethoprim^d^	A	1 025 123	62.9 (52.0-71.8)	915 649 (89.3)	109 474 (10.7)	42 145	32	5.1 (3.5-7.3)
Ciprofloxacin^d^	B	1 125 460	64.8 (56.3-73.2)	1 028 351 (91.4)	97 109 (8.6)	36 803	29	5.1 (3.5-7.4)

^a^ Rates of severe acute liver injury were assessed among patients without preexisting liver or biliary disease and after censoring for incident diagnoses of liver or biliary diseases or other conditions that may precipitate findings consistent with liver injury.

^b^ Based on published reports. Original category as defined by Björnsson and Hoofnagle.^6^ Medications listed in the US National Institutes of Health LiverTox database were classified into categories of likelihood for causing acute liver injury based on the number of published reports of hepatotoxicity (category A, ≥50 cases; category B, 12-49 cases; category C, 4-11 cases).

^c^ Incidence rates adjusted for age and sex.

^d^ Represents antimicrobial (ie, antibacterial, antiviral, or antifungal) medications.

### Sensitivity Analyses

After additionally adjusting for diabetes, hyperlipidemia, and obesity, rates of severe ALI remained similar (eFigures 1-3 in Supplement 1). Second, among medications with rates that could be estimated with sufficient precision, the groupings were similar in males and females (eTable 9 in Supplement 1). The rate of severe ALI with estrogen or progestin medications was higher in males than females (10.4 vs 0.0 events per 10 000 person-years). Third, after limiting each cohort to persons younger than 65 years, severe ALI rates were slightly lower in magnitude compared with the primary analysis. Rates of severe ALI for captopril and sulfamethoxazole decreased from 5.8 to 2.7 and 5.1 to 3.1 events per 10 000 person-years, respectively; however, the groupings of the remaining medications remained preserved (eFigures 4-6 in Supplement 1). Fourth, after excluding persons additionally dispensed groups 1 and 2 medications 90 or fewer days prior to suspected hepatotoxic drug initiation (eTable 10 in Supplement 1), rates of severe ALI for groups 1 and 2 medications were slightly lower than in the primary analysis (eFigures 7-9 in Supplement 1). Rates of outcomes for fluconazole and captopril decreased from 6.0 to 1.8 and from 5.8 to 2.6 events per 10 000 person-years, respectively, but groupings for other medications were generally preserved with the recalculated rates. When severe ALI in the presence of polypharmacy was examined, rates of outcomes were similar to the primary analysis and groupings were preserved (eFigures 10-12 in Supplement 1).

## Discussion

This series of cohort studies used real-world data to measure incidence rates of hospitalization for severe ALI following outpatient initiation of suspected hepatotoxic medications among persons without preexisting liver or biliary disease. The analyses identified 17 medications (groups 1 and 2) with the highest rates of severe ALI. These drugs remained the most potentially hepatotoxic after excluding concomitant users of other medications of groups 1 and 2 and with polypharmacy. Categorization of hepatotoxicity based on the number of published case reports did not accurately reflect observed rates of severe ALI.

Antimicrobial medications represented 64% of the medications from groups 1 and 2 with the highest rates of severe ALI. Antifungal and older antiretroviral medications have been particularly implicated as hepatotoxic.^22,23^ This is consistent with our findings.

This study represents a systematic, reproducible approach to using real-world data to measure rates of severe ALI following medication initiation among patients without liver or biliary disease. Regulators and clinicians have relied on published case reports to assess the hepatic safety of drugs, but our results show that the number of reports inaccurately represents rates of severe ALI. We created cohorts of new initiators of outpatient medications, defined the period of exposure as the time receiving medication through 30 days after discontinuation (to capture events shortly after cessation) and evaluated a composite laboratory-based severe ALI outcome indicating significant hepatic injury. We reduced the likelihood of capturing non–drug-related severe ALI by censoring as nonevents patients with liver or biliary disease or other conditions precipitating ALI. We accounted for potential effects of drug-drug interactions by examining rates of outcomes after exclusion of concomitant users of groups 1 and 2 drugs and those with polypharmacy; results showed that medications from groups 1 and 2 remained the most hepatotoxic. These methods could allow measurement of rates of severe ALI for medications and enable evaluation of hepatotoxicity safety signals from spontaneous reports.

The study has important implications for clinical care. Patients initiating a medication with a high rate of severe ALI might require closer monitoring of liver-related laboratory tests to detect evolving hepatic dysfunction earlier, which might improve prognosis. Within electronic health record systems, automated messages could alert clinicians ordering a high-risk medication to the potential for severe ALI and to consider laboratory monitoring. The results also provide real-world evidence of the hepatic safety of certain medications. For example, although some statins were included in category A or B based on reported cases of hepatotoxicity, rates of severe ALI for these medications were low (<1.0 event per 10 000 person-years).

Our method of measuring rates of severe ALI after suspected hepatotoxic medication initiation could be applied within other electronic medical record data outside the VA system. The approach could be modified to identify new potentially hepatotoxic drugs by looking back from hospitalizations for severe ALI and examining associations with proximal medications. This method could also be expanded to examine other medication-related toxic effects on organs.

### Limitations

Our study has several limitations. First, we did not perform a causality assessment of all outcomes. Within a random sample of severe ALI events, 76% were deemed medication related by hepatologist review. Some severe ALI events might have been caused by herbal and dietary supplements, over-the-counter drugs such as acetaminophen, or other hepatotoxic medications that were not ascertained. Some events may have been due to non–drug-related conditions that evaded censoring because they were not recorded as discharge diagnoses. We also might have missed steatotic liver disease among individuals without baseline liver-related laboratory tests. However, there is no reason to believe that these products or conditions were differentially distributed across cohorts.

Second, because ascertainment of ALI relied on laboratory tests, surveillance bias may have contributed to the results, as clinicians might have performed more testing on persons dispensed medications with a greater number of hepatotoxicity reports. Third, our approach might have underestimated rates of outcomes, since veterans may present for emergent care outside the VA. Fourth, the 95% CIs around the severe ALI incidence rates could have been too narrow, as they did not account for the rate of outcome misclassification. Fifth, because we did not perform hypothesis testing or make formal statistical comparisons in rates between the medications of interest, we did not apply adjustment for multiple comparisons in our results.

Sixth, while we examined medication count as a proxy for drug-drug interactions, it was beyond the scope of our analyses to examine rates of severe ALI with specific combinations of medications. Seventh, we did not evaluate the influence of medication dose on severe ALI rates. Eighth, we did not examine rates of severe ALI after initiation of medications that had 1 to 3 published reports of hepatotoxicity. Instead, we focused on medications with the highest number of hepatotoxicity reports, since these are likely to be perceived as having the highest risk of severe ALI. Future studies should evaluate these other medications. Ninth, our study included predominantly male patients without liver or biliary disease. Results may not be generalizable to females or individuals with liver disease.

## Conclusions

This series of cohort studies demonstrated that real-world data can be used to measure rates of hospitalization for severe ALI following outpatient initiation of suspected hepatotoxic medications among persons without preexisting liver or biliary disease. We identified medications with the highest rates of severe ALI after censoring for non–drug-related causes. Categorization of hepatotoxicity by case reports did not accurately reflect severe ALI rates using real-world data. This study provides a framework for investigating postmarketing hepatotoxicity safety signals. Future studies should evaluate rates of severe ALI among persons with chronic liver disease to provide evidence on the hepatic safety of medications in these patients.

## Notes

Supplement 1. **eMethods.** Estimate of the Frequency With Which Hospitalizations for Severe Acute Liver Injury (ALI) Were Possibly Caused by a Medication **eTable 1.** Categories of Medications Classified as Suspected Hepatotoxic Based on Numbers of Published Cases of Idiosyncratic Drug-Induced Liver Injury **eTable 2.** List of International Classification of Diseases, Ninth Revision (ICD-9) and International Classification of Diseases, Tenth Revision (ICD-10) Diagnostic Codes Used to Identify Liver/Biliary Disease for Exclusion and Censoring **eTable 3.** List of International Classification of Diseases, Ninth Revision (ICD-9) and International Classification of Diseases, Tenth Revision (ICD-10) Diagnostic Codes Used to Identify Certain Conditions That Could Precipitate Findings Consistent With Severe Acute Liver Injury for Exclusion and Censoring **eTable 4.** List of International Classification of Diseases, Ninth Revision (ICD-9) and International Classification of Diseases, Tenth Revision (ICD-10) Diagnostic Codes Used to Identify Diabetes Mellitus and Hyperlipidemia **eTable 5.** List of International Classification of Diseases, Ninth Revision (ICD-9) and International Classification of Diseases, Tenth Revision (ICD-10) Diagnostic Codes and Current Procedural Terminology (CPT) Procedure Codes Used to Identify Liver Transplantation **eTable 6.** Reasons for Exclusion of Patients Within Each of the 194 Medication Initiator Cohorts **eTable 7.** Age-/Sex-Adjusted Incidence Rates of Severe Acute Liver Injury Among Initiators of 91 Medications That Were Not Included in the Primary Analysis Because Either the Width of the 95% Confidence Interval for the Severe Acute Liver Injury Incidence Rate was ≥3 Times the Point Estimate or There was <10,000 Person-Years of Follow-Up Present **eTable 8.** Incidence Rates of Severe Acute Liver Injury for Group 3-5 Medications With ≥4 Published Reports of Hepatotoxicity (Categories A, B, C) **eTable 9.** Incidence Rates of Severe Acute Liver Injury by Sex for Medications With Rates That Could be Estimated With Sufficient Precision **eTable 10.** Most Common Group 1 and Group 2 Medications That Were Additionally Dispensed Within 90 Days of Initiation of Each Medication of Interest **eFigure 1.** Rates of Severe Acute Liver Injury for Group 1-2 Initiators Additionally Adjusted for Metabolic Comorbidities **eFigure 2.** Rates of Severe Acute Liver Injury for Group 3-4 Initiators Additionally Adjusted for Metabolic Comorbidities **eFigure 3.** Rates of Severe Acute Liver Injury for Group 5 Initiators Additionally Adjusted for Metabolic Comorbidities **eFigure 4.** Rates of Severe Acute Liver Injury for Group 1-2 Initiators <65 Years of Age **eFigure 5.** Rates of Severe Acute Liver Injury for Group 3-4 Initiators <65 Years of Age **eFigure 6.** Rates of Severe Acute Liver Injury for Group 5 Initiators <65 Years of Age **eFigure 7.** Rates of Severe Acute Liver Injury for Group 1-2 Initiators Additionally Dispensed Group 1-2 Medications **eFigure 8.** Rates of Severe Acute Liver Injury for Group 3-4 Initiators Additionally Dispensed Group 1-2 Medications **eFigure 9.** Rates of Severe Acute Liver Injury for Group 5 Initiators Additionally Dispensed Group 1-2 Medications **eFigure 10.** Rates of Severe Acute Liver Injury for Group 1-2 Initiators Dispensed ≥5 Unique Medications **eFigure 11.** Rates of Severe Acute Liver Injury for Group 3-4 Initiators Dispensed ≥5 Unique Medications **eFigure 12.** Rates of Severe Acute Liver Injury for Group 5 Initiators Dispensed ≥5 Unique Medications

Supplement 2.
Data Sharing Statement
