# Supplementary material for: Severe Acute Liver Injury After Hepatotoxic Medication Initiation in Real-World Data
Source: JAMA Intern Med. 2024 Jun 24;184(8):943–52. doi: 10.1001/jamainternmed.2024.1836 (PMC11197444; doi:10.1001/jamainternmed.2024.1836)
Supplement: Supplement 1. — eMethods. Estimate of the Frequency With Which Hospitalizations for Severe Acute Liver Injury (ALI) Were Possibly Caused by a Medication eTable 1. Categories of Medications Classified as Suspected Hepatotoxic Based on Numbers of Published Cases of Idiosyncratic Drug-Induced Liver Injury eTable 2. List of International Classification of Diseases, Ninth Revision (ICD-9) and International Classification of Diseases, Tenth Revision (ICD-10) Diagnostic Codes Used to Identify Liver/Biliary Disease for Exclusion and Censoring eTable 3. List of International Classification of Diseases, Ninth Revision (ICD-9) and International Classification of Diseases, Tenth Revision (ICD-10) Diagnostic Codes Used to Identify Certain Conditions That Could Precipitate Findings Consistent With Severe Acute Liver Injury for Exclusion and Censoring eTable 4. List of International Classification of Diseases, Ninth Revision (ICD-9) and International Classification of Diseases, Tenth Revision (ICD-10) Diagnostic Codes Used to Identify Diabetes Mellitus and Hyperlipidemia eTable 5. List of International Classification of Diseases, Ninth Revision (ICD-9) and International Classification of Diseases, Tenth Revision (ICD-10) Diagnostic Codes and Current Procedural Terminology (CPT) Procedure Codes Used to Identify Liver Transplantation eTable 6. Reasons for Exclusion of Patients Within Each of the 194 Medication Initiator Cohorts eTable 7. Age-/Sex-Adjusted Incidence Rates of Severe Acute Liver Injury Among Initiators of 91 Medications That Were Not Included in the Primary Analysis Because Either the Width of the 95% Confidence Interval for the Severe Acute Liver Injury Incidence Rate was ≥3 Times the Point Estimate or There was <10,000 Person-Years of Follow-Up Present eTable 8. Incidence Rates of Severe Acute Liver Injury for Group 3-5 Medications With ≥4 Published Reports of Hepatotoxicity (Categories A, B, C) eTable 9. Incidence Rates of Severe Acute Liver Injury by Sex for Medications With Rates [file jamainternmed-e241836-s001.pdf]

Supplementary Online Content

Torgersen J, Mezochow AK, Newcomb CW, et al. Severe acute liver injury after hepatotoxic medication initiation in real-world data. *JAMA Intern Med.* Published online June 24, 2024. doi:10.1001/jamainternmed.2024.1836

eMethods. Estimate of the Frequency With Which Hospitalizations for Severe Acute Liver Injury (ALI) Were Possibly Caused by a Medication

eTable 1. Categories of Medications Classified as Suspected Hepatotoxic Based on Numbers of Published Cases of Idiosyncratic Drug-Induced Liver Injury

eTable 2. List of International Classification of Diseases, Ninth Revision (ICD-9) and International Classification of Diseases, Tenth Revision (ICD-10) Diagnostic Codes Used to Identify Liver/Biliary Disease for Exclusion and Censoring

eTable 3. List of International Classification of Diseases, Ninth Revision (ICD-9) and International Classification of Diseases, Tenth Revision (ICD-10) Diagnostic Codes Used to Identify Certain Conditions That Could Precipitate Findings Consistent With Severe Acute Livery Injury for Exclusion and Censoring

eTable 4. List of International Classification of Diseases, Ninth Revision (ICD-9) and International Classification of Diseases, Tenth Revision (ICD-10) Diagnostic Codes Used to Identify Diabetes Mellitus and Hyperlipidemia

eTable 5. List of International Classification of Diseases, Ninth Revision (ICD-9) and International Classification of Diseases, Tenth Revision (ICD-10) Diagnostic Codes and Current Procedural Terminology (CPT) Procedure Codes Used to Identify Liver Transplantation

eTable 6. Reasons for Exclusion of Patients Within Each of the 194 Medication Initiator Cohorts

eTable 7. Age-/Sex-Adjusted Incidence Rates of Severe Acute Liver Injury Among Initiators of 91 Medications That Were Not Included in the Primary Analysis Because Either the Width of the 95% Confidence Interval for the Severe Acute Liver Injury Incidence Rate was ≥3 Times the Point Estimate or There was <10,000 Person-Years of Follow-Up Present

eTable 8. Incidence Rates of Severe Acute Liver Injury for Group 3-5 Medications With ≥4 Published Reports of Hepatotoxicity (Categories A, B, C)

eTable 9. Incidence Rates of Severe Acute Liver Injury by Sex for Medications With Rates That Could be Estimated With Sufficient Precision

eTable 10. Most Common Group 1 and Group 2 Medications That Were Additionally Dispensed Within 90 Days of Initiation of Each Medication of Interest

eFigure 1. Rates of Severe Acute Liver Injury for Group 1-2 Initiators Additionally Adjusted for Metabolic Comorbidities

eFigure 2. Rates of Severe Acute Liver Injury for Group 3-4 Initiators Additionally Adjusted for Metabolic Comorbidities

eFigure 3. Rates of Severe Acute Liver Injury for Group 5 Initiators Additionally Adjusted for Metabolic Comorbidities

eFigure 4. Rates of Severe Acute Liver Injury for Group 1-2 Initiators <65 Years of Age

eFigure 5. Rates of Severe Acute Liver Injury for Group 3-4 Initiators <65 Years of Age

eFigure 6. Rates of Severe Acute Liver Injury for Group 5 Initiators <65 Years of Age

eFigure 7. Rates of Severe Acute Liver Injury for Group 1-2 Initiators Additionally Dispensed Group 1-2 Medications

eFigure 8. Rates of Severe Acute Liver Injury for Group 3-4 Initiators Additionally Dispensed Group 1-2 Medications

eFigure 9. Rates of Severe Acute Liver Injury for Group 5 Initiators Additionally Dispensed Group 1-2 Medications

eFigure 10. Rates of Severe Acute Liver Injury for Group 1-2 Initiators Dispensed ≥5 Unique Medications

eFigure 11. Rates of Severe Acute Liver Injury for Group 3-4 Initiators Dispensed ≥5 Unique Medications

eFigure 12. Rates of Severe Acute Liver Injury for Group 5 Initiators Dispensed ≥5 Unique Medications

This supplementary material has been provided by the authors to give readers additional information about their work.

eMethods. Estimate of the frequency with which hospitalizations for severe acute liver injury (ALI) were possibly caused by a medication.

There is no accepted international gold standard for determining drug-induced causality of severe ALI.[1] Therefore, in a sample of patients with a hospitalization for severe ALI in the primary study, we estimated the frequency with which a medication caused or contributed to the severe ALI based on review of medical records by hepatologists.

Among the initiators of suspected hepatotoxic medications (defined by at least four published reports of hepatotoxicity based on assessment of the LiverTox website[2]) in the primary study, we selected a random sample of 75 patients electronically identified as having a hospitalization for severe ALI. Two hepatologists independently reviewed these patients' electronic medical records, including hospital admission notes, gastroenterology/hepatology notes, other specialist consultation notes, hospital progress notes, hospital laboratory results, and discharge summaries.

Each hepatologist adjudicator first separately assessed whether the patient had a condition that might exclude drug-induced ALI as a cause of the severe ALI, including: 1) ischemic events, 2) vascular abnormalities, 3) viral hepatitis, 4) pancreaticobiliary disease, 5) hepatic infiltrative disease, 6) non-viral hepatitis, and 7) metabolic/genetic disorders (detailed in **eMethods Table 1**).

eMethods Table 1. Non-drug-related categories and conditions of severe acute liver injury with methods of confirmation.

Category of Non-Drug-Related ALI	Conditions	Methods of Confirmation
Ischemic events	Cardiogenic, hypovolemic, septic shock	Acute elevations in liver aminotransferases in context of documented hypoperfusion event
Vascular abnormality	Congestive hepatopathy; hepatic vein thrombosis (Budd-Chiari syndrome)	History, abdominal imaging
Viral hepatitis	Hepatitis A, B, C, D, E; cytomegalovirus; Epstein-Barr virus; herpes simplex virus	Serologic or virologic tests
Pancreaticobiliary disease	Cholangitis, cholecystitis, choledocholithiasis, pancreaticobiliary cancer, primary sclerosing cholangitis	History, abdominal imaging, pathology
Hepatic infiltrative disease	Hepatocellular carcinoma, hepatic metastasis of cancer, leukemia/lymphoma	History, abdominal imaging, pathology
Non-viral hepatitis	Alcoholic hepatitis/liver disease, alpha-1-antitrypsin deficiency, autoimmune hepatitis	History, serology, pathology
Metabolic/genetic disorders	Celiac disease, hereditary hemochromatosis, rhabdomyolysis, thyrotoxicosis, Wilson's disease	Clinical history, serology, pathology

Abbreviations: ALI, acute liver injury

Next, the hepatologists determined if a medication possibly caused or contributed to this patient's severe ALI based on clinical, biochemical, and/or histological evidence; time between the start of a medication and the onset of ALI; clinical signature; evidence of improvement of the ALI upon medication withdrawal (de-challenge; if applicable); and the effect of any rechallenge.[1,3] Since drug-induced ALI is a challenging and subjective diagnosis to confirm,[1,3] even for experts, we classified a severe ALI event as medication-related if *either* hepatologist indicated a possible drug-induced causality.

We estimated that a sample of 75 patients with a hospitalization for severe ALI would allow determination of the proportion that had a medication as the etiology with a maximum 95% confidence interval of ±10%, assuming at least 80% had drug-induced severe ALI.[4] Data were analyzed using SAS 9.4 (SAS Institute Inc., Cary, NC).

Among the 75 patients selected for medical record review, 57 (76.0%; 95% confidence interval, 64.7-85.1%) had a hospitalization for severe ALI that was deemed by a hepatologist to be medication-related. **eMethods Table 2** reports the causes of all non-drug-related severe ALI events as adjudicated by the hepatologist reviewers. Among the 18 patients classified as not having had severe ALI caused by a medication, the most common diagnoses identified by the hepatologists as likely causing their severe ALI were congestive hepatopathy (n=7; 38.9%), hypovolemic shock (n=4; 22.2%), alcoholic hepatitis (n=4; 22.2%) and sepsis (n=4; 22.2%).

eMethods Table 2. Etiologies of severe acute liver events that were adjudicated as non-drug-related by hepatologists.

Patient	Non-Drug-Related Etiologies for Hospitalization for Severe Acute Liver Injury*
1	Pancreaticobiliary cancer; sepsis
2	Hemolysis; sepsis
3	Sepsis; rhabdomyolysis
4	Cardiovascular shock; hypovolemic shock
5	Hypovolemic shock
6	Congestive hepatopathy; SIRS; alcoholic hepatitis
7	Congestive hepatopathy; alcoholic hepatitis
8	Lymphoma; cardiovascular shock
9	Congestive hepatopathy; rhabdomyolysis
10	Congestive hepatopathy
11	Choledocholithiasis; hypovolemic shock; SIRS
12	Small bowel obstruction; cardiovascular shock
13	Sepsis; congestive hepatopathy
14	Alcoholic hepatitis; congestive hepatopathy
15	Hemorrhagic shock; SIRS
16	Colonic perforation; cancer
17	Congestive hepatopathy; alcoholic hepatitis; hepatitis C
18	Hypovolemic shock

Abbreviations: SIRS, systemic inflammatory response syndrome
*Adjudicators could indicate multiple possible etiologies for a non-drug-related severe acute liver injury event.

REFERENCES

1. Rockey DC, Seeff LB, Rochon J, et al. Causality assessment in drug-induced liver injury using a structured expert opinion process: comparison to the Roussel-Uclaf causality assessment method. *Hepatology.* 2010;51(6):2117-2126.
2. Bjornsson ES, Hoofnagle JH. Categorization of drugs implicated in causing liver injury: Critical assessment based on published case reports. *Hepatology.* 2016;63(2):590-603.
3. Fontana RJ, Liou I, Reuben A, et al. AASLD practice guidance on drug, herbal, and dietary supplement-induced liver injury. *Hepatology.* 2023;77(3):1036-1065.
4. Weinstein EJ, Ritchey ME, Lo Re V, 3rd. Core concepts in pharmacoepidemiology: Validation of health outcomes of interest within real-world healthcare databases. *Pharmacoepidemiol Drug Saf.* 2023;32(1):1-8.

eTable 1. Categories of medications classified as suspected hepatotoxic based on numbers of published cases of idiosyncratic drug-induced liver injury (category A, ≥50 cases; category B, 12-49 cases; category C, 4-11 cases). Italicized medications (n=26) were unable to be evaluated because they were not marketed in the United States,[a] not dispensed within the VA during the study period;[b] administered via injection/intravenous route;[c] anticoagulants;[d] or used for alcohol use disorder or liver disease treatment.[e]

#	Category A (n=48)	Category B (n=76)	Category C (n=96)
1	Allopurinol	Acarbose	Abacavir
2	Amiodarone	Amitriptyline	Acebutolol
3	Androgenic steroids[f]	*Amodiaquine*[a]	Acitretin
4	Atorvastatin	Amoxicillin	Adalimumab
5	Auranofin or gold products[f]	Asparaginase or pegaspargase	Albendazole
6	Azathioprine mercaptopurine	Azithromycin	Alfuzosin
7	Busulfan	Captopril	Amlodipine
8	Carbamazepine	*Cefazolin*[c]	Amphetamines[g]
9	Chlorpromazine	Ceftriaxone	*Amphotericin*[c]
10	Clavulanate with amoxicillin	Celecoxib	*Ampicillin*[c]
11	Dantrolene	Chlorpropamide	Anakinra
12	Diclofenac	Chlorzoxazone	Atomoxetine
13	Didanosine	Cimetidine	Bosentan
14	*Disulfiram*[e]	Ciprofloxacin	Bupropion
15	Efavirenz	Clarithromycin	Candesartan
16	Erythromycin	Clindamycin	Cephalexin
17	Estrogens or progestins[f]	Clopidogrel	Citalopram or escitalopram
18	*Floxuridine*[c]	Cloxacillin	Clomiphene
19	*Flucloxacillin*[a]	Clozapine	Cyproheptadine
20	Flutamide	Cyclophosphamide or ifosphamide	Cytarabine
21	*Halothane*[b]	*Cyproterone*[a]	*Dactinomycin*[c]
22	Hydralazine	*Dacarbazine*[c]	*Daptomycin*[c]
23	Ibuprofen	Dicloxacillin	*Desflurane*[c]
24	Infliximab	Doxorubicin	Diflunisal
25	*Interferon-α or peginterferon*[e]	Duloxetine	Diltiazem
26	Interferon-β	Enalapril	Disopyramide
27	Isoniazid	*Enflurane*[c]	Doxycycline
28	Ketoconazole	Etanercept	*Dronedarone*[b]
29	Methotrexate	Ethionamide	Erlotinib
30	Methyldopa	Felbamate	Etodolac
31	Minocycline	Fenofibrate	Etoposide
32	Nevirapine	Fluconazole	Ezetimibe
33	*Nimesulide*[a]	Fluvastatin	Famotidine
34	Nitrofurantoin	Glyburide (Glibenclamide)	Flavocoxid
35	Phenytoin of fosphenytoin	Haloperidol	Fluorouracil
36	Propylthiouracil	*Heparin*[d]	Fluoxetine
37	Pyrazinamide	Imatinib	Gabapentin
38	Quinidine	Imipramine	Gefitinib
39	Rifampin	Irinotecan	Gemcitabine
40	Simvastatin	*Isoflurane*[c]	Gemfibrozil
41	Sulfamethoxazole with trimethoprim	Itraconazole	Glimepiride
42	Sulfasalazine	*Ketamine*[c]	Glipizide
43	*Sulfonamides (unspecified)*[b,f]	Lamotrigine	Hydroxyurea
44	Sulindac	Leflunomide	Indomethacin
45	Telithromycin	Levofloxacin or ofloxacin	Irbesartan
46	Thioguanine	Lisinopril	Isotretinoin

#	Category A (n=48)	Category B (n=76)	Category C (n=96)
47	Ticlopidine	Lovastatin	Ketoprofen
48	Valproate	Melphalan	Labetalol
49		Metformin	Lansoprazole or dexlansoprazole
50		Methimazole (Thiamazole)	Lenalidomide or thalidomide
51		Moxifloxacin	Levetiracetam
52		Naproxen	Levocetirizine or cetirizine
53		Nifedipine	Linezolid
54		Olanzapine	Losartan
55		Omeprazole or esomeprazole	Meloxicam
56		Oxacillin	Mesalamines[h]
57		Oxaliplatin	Methoxsalen
58		Paroxetine	Methylphenidate or dexmethylphenidate
59		Penicillamine	Metronidazole
60		Phenobarbital	Mexiletine
61		Piroxicam	Mirtazapine
62		Propafenone	Mitomycin
63		Quinine	Montelukast
64		Ranitidine	*Nafcillin*[c]
65		*Rivaroxaban*[d]	Natalizumab
66		Rosuvastatin	Nefazodone
67		Sertraline	Nilutamide
68		*Sevoflurane*[c]	Norfloxacin
69		Stavudine	Orlistat
70		Tamoxifen	Oxaprozin
71		Terbinafine	Pantoprazole
72		Thiabendazole	Penicillin G
73		Thioridazine	Phenelzine
74		Venlafaxine or dexvenlafaxine	Pioglitazone
75		Voriconazole	Pravastatin
76		Zidovudine	Pregabalin
77			Procainamide
78			Prochlorperazine
79			Pyrimethamine
80			Quetiapine
81			Ramipril
82			Risperidone
83			Ritonavir
84			Rosiglitazone
85			Sorafenib
86			Temozolomide
87			Tolcapone
88			Topiramate
89			Trastuzumab
90			Trazodone
91			*Trimethoprim*[b]
92			Vancomycin
93			Verapamil
94			Vincristine
95			*Warfarin*[d]
96			Zafirlukast

Original category as defined by Björnsson and Hoofnagle, *Hepatology* 2016;63:590-603. Medications listed in the National Institutes of Health LiverTox database were classified into categories of likelihood for causing acute liver injury based on the number of published reports of hepatotoxicity (category A, ≥50 cases; category B, 12-49 cases; category C, 4-11 cases).

[a] Not evaluated because the medication was not marketed in the United States during the study period.

[b] Not evaluated because the medication was not dispensed within the VA during the study period.
[c] Not evaluated because the medication is administered via injection or intravenous route.
[d] Not evaluated because the medication is an anticoagulant.
[e] Not evaluated because the medication is used for alcohol use disorder or liver disease treatment.
[f] Represents groups of medications.
[g] Includes amphetamine, dextroamphetamine, lisdexamfetamine, and methamphetamine.
[h] Includes mesalamine, balsalazide, and olsalazine.

eTable 2. List of International Classification of Diseases, Ninth Revision (ICD-9) and International Classification of Diseases, Tenth Revision (ICD-10) diagnostic codes used to identify liver/biliary disease for exclusion and censoring. Five diagnostic codes in the other unspecified hepatitis/liver disease category were not used for censoring because of the potential for clinicians to record drug-induced liver injury with these codes.

Type of Liver/Biliary Disease	Code Type	Code	Description
Alcoholic liver disease	ICD-9	571.0	Alcoholic fatty liver
Alcoholic liver disease	ICD-9	571.1	Acute alcoholic hepatitis
Alcoholic liver disease	ICD-9	571.2	Alcoholic cirrhosis of the liver
Alcoholic liver disease	ICD-9	571.3	Alcoholic liver damage, unspecified
Alcoholic liver disease	ICD-10	K70	Alcoholic liver disease
Alcoholic liver disease	ICD-10	K70.0	Alcoholic fatty liver
Alcoholic liver disease	ICD-10	K70.1	Alcoholic hepatitis
Alcoholic liver disease	ICD-10	K70.10	Alcoholic hepatitis without ascites
Alcoholic liver disease	ICD-10	K70.11	Alcoholic hepatitis with ascites
Alcoholic liver disease	ICD-10	K70.2	Alcoholic fibrosis/sclerosis of the liver
Alcoholic liver disease	ICD-10	K70.3	Alcoholic cirrhosis of liver
Alcoholic liver disease	ICD-10	K70.30	Alcoholic cirrhosis of liver without ascites
Alcoholic liver disease	ICD-10	K70.31	Alcoholic cirrhosis of liver with ascites
Alcoholic liver disease	ICD-10	K70.4	Alcoholic hepatic failure
Alcoholic liver disease	ICD-10	K70.40	Alcoholic hepatic failure without coma
Alcoholic liver disease	ICD-10	K70.41	Alcoholic hepatic failure with coma
Alcoholic liver disease	ICD-10	K70.9	Alcoholic liver disease, unspecified
Alpha-1-antitrypsin deficiency	ICD-9	273.4	Alpha-1-antitrypsin deficiency
Alpha-1-antitrypsin deficiency	ICD-10	E88.01	Alpha-1-antitrypsin deficiency
Autoimmune hepatitis	ICD-9	571.42	Autoimmune hepatitis
Autoimmune hepatitis	ICD-10	K75.4	Autoimmune hepatitis
Biliary disease	ICD-9	574	Cholelithiasis
Biliary disease	ICD-9	574.0	Calculus of gallbladder with acute cholecystitis
Biliary disease	ICD-9	574.00	Calculus of gallbladder with acute cholecystitis, without mention of obstruction
Biliary disease	ICD-9	574.01	Calculus of gallbladder with acute cholecystitis, with obstruction
Biliary disease	ICD-9	574.1	Calculus of gallbladder with other cholecystitis
Biliary disease	ICD-9	574.10	Calculus of gallbladder with other cholecystitis, without mention of obstruction
Biliary disease	ICD-9	574.11	Calculus of gallbladder with other cholecystitis, with obstruction
Biliary disease	ICD-9	574.2	Calculus of gallbladder without mention of cholecystitis

Type of Liver/Biliary Disease	Code Type	Code	Description
Biliary disease	ICD-9	574.20	Calculus of gallbladder without mention of cholecystitis, without mention of obstruction
Biliary disease	ICD-9	574.21	Calculus of gallbladder without mention of cholecystitis, with obstruction
Biliary disease	ICD-9	574.3	Calculus of bile duct with acute cholecystitis
Biliary disease	ICD-9	574.30	Calculus of bile duct with acute cholecystitis, without mention of obstruction
Biliary disease	ICD-9	574.31	Calculus of bile duct with acute cholecystitis, with obstruction
Biliary disease	ICD-9	574.4	Calculus of bile duct with other cholecystitis
Biliary disease	ICD-9	574.40	Calculus of bile duct with other cholecystitis, without mention of obstruction
Biliary disease	ICD-9	574.41	Calculus of bile duct with other cholecystitis, with obstruction
Biliary disease	ICD-9	574.5	Calculus of bile duct without mention of cholecystitis
Biliary disease	ICD-9	574.50	Calculus of bile duct without mention of cholecystitis, without mention of obstruction
Biliary disease	ICD-9	574.51	Calculus of bile duct without mention of cholecystitis, with obstruction
Biliary disease	ICD-9	574.6	Calculus of gallbladder and bile duct with acute cholecystitis
Biliary disease	ICD-9	574.60	Calculus of gallbladder and bile duct with acute cholecystitis, without mention of obstruction
Biliary disease	ICD-9	574.61	Calculus of gallbladder and bile duct with acute cholecystitis, with obstruction
Biliary disease	ICD-9	574.7	Calculus of gallbladder and bile duct with other cholecystitis
Biliary disease	ICD-9	574.70	Calculus of gallbladder and bile duct with other cholecystitis, without mention of obstruction
Biliary disease	ICD-9	574.71	Calculus of gallbladder and bile duct with other cholecystitis, with obstruction
Biliary disease	ICD-9	574.8	Calculus of gallbladder and bile duct with acute and chronic cholecystitis
Biliary disease	ICD-9	574.80	Calculus of gallbladder and bile duct with acute and chronic cholecystitis, without mention of obstruction
Biliary disease	ICD-9	574.81	Calculus of gallbladder and bile duct with acute and chronic cholecystitis, with obstruction
Biliary disease	ICD-9	574.9	Calculus of gallbladder and bile duct without cholecystitis
Biliary disease	ICD-9	574.90	Calculus of gallbladder and bile duct without cholecystitis, without mention of obstruction
Biliary disease	ICD-9	574.91	Calculus of gallbladder and bile duct without cholecystitis, with obstruction
Biliary disease	ICD-9	575	Other disorders of gallbladder
Biliary disease	ICD-9	575.0	Acute cholesystitis
Biliary disease	ICD-9	575.1	Other cholecystitis
Biliary disease	ICD-9	575.10	Cholecyctitis, unspecified
Biliary disease	ICD-9	575.11	Chronic cholecystitis
Biliary disease	ICD-9	575.12	Acute and chronic cholesystitis
Biliary disease	ICD-9	575.2	Obstruction of gallbladder
Biliary disease	ICD-9	575.3	Hydrops of gallbladder
Biliary disease	ICD-9	575.4	Perforation of gallbladder
Biliary disease	ICD-9	575.5	Fistula of gallbladder

Type of Liver/Biliary Disease	Code Type	Code	Description
Biliary disease	ICD-9	575.9	Unspecified disorder of gallbladder
Biliary disease	ICD-9	576.1	Cholangitis
Biliary disease	ICD-9	576.2	Obstruction of bile duct
Biliary disease	ICD-9	576.3	Perforation of bile duct
Biliary disease	ICD-9	576.4	Fistula of bile duct
Biliary disease	ICD-9	576.9	Unspecified disorder of biliary tract
Biliary disease	ICD-9	575.8	Other specified disorders of gallbladder
Biliary disease	ICD-9	576.8	Other specified disorders of biliary tract
Biliary disease	ICD-10	K80	Cholelithiasis
Biliary disease	ICD-10	K80.0	Calculus of gallbladder with acute cholecystitis
Biliary disease	ICD-10	K80.00	Calculus of gallbladder with acute cholecystitis without obstruction
Biliary disease	ICD-10	K80.01	Calculus of gallbladder with acute cholecystitis with obstruction
Biliary disease	ICD-10	K80.1	Calculus of gallbladder with other cholecystitis
Biliary disease	ICD-10	K80.10	Calculus of gallbladder with chronic cholecystitis without obstruction
Biliary disease	ICD-10	K80.11	Calculus of gallbladder with chronic cholecystitis with obstruction
Biliary disease	ICD-10	K80.12	Calculus of gallbladder with acute and chronic cholecystitis without obstruction
Biliary disease	ICD-10	K80.13	Calculus of gallbladder with acute and chronic cholecystitis with obstruction
Biliary disease	ICD-10	K80.18	Calculus of gallbladder with other cholecystitis without obstruction
Biliary disease	ICD-10	K80.19	Calculus of gallbladder with other cholecystitis with obstruction
Biliary disease	ICD-10	K80.2	Calculus of gallbladder without cholecystitis
Biliary disease	ICD-10	K80.20	Calculus of gallbladder without cholecystitis without obstruction
Biliary disease	ICD-10	K80.21	Calculus of gallbladder without cholecystitis with obstruction
Biliary disease	ICD-10	K80.3	Calculus of bile duct with cholangitis
Biliary disease	ICD-10	K80.30	Calculus of bile duct with cholangitis unspecified, without obstruction
Biliary disease	ICD-10	K80.31	Calculus of bile duct with cholangitis unspecified, with obstruction
Biliary disease	ICD-10	K80.32	Calculus of bile duct with acute cholangitis without obstruction
Biliary disease	ICD-10	K80.33	Calculus of bile duct with acute cholangitis with obstruction
Biliary disease	ICD-10	K80.34	Calculus of bile duct with chronic cholangitis without obstruction
Biliary disease	ICD-10	K80.35	Calculus of bile duct with chronic cholangitis with obstruction
Biliary disease	ICD-10	K80.36	Calculus of bile duct with acute and chronic cholangitis without obstruction
Biliary disease	ICD-10	K80.37	Calculus of bile duct with acute and chronic cholangitis with obstruction
Biliary disease	ICD-10	K80.4	Calculus of bile duct with cholecystitis
Biliary disease	ICD-10	K80.40	Calculus of bile duct with cholecystitis unspecified, without obstruction
Biliary disease	ICD-10	K80.41	Calculus of bile duct with cholecystitis unspecified, with obstruction

Type of Liver/Biliary Disease	Code Type	Code	Description
Biliary disease	ICD-10	K80.42	Calculus of bile duct with acute cholecystitis without obstruction
Biliary disease	ICD-10	K80.43	Calculus of bile duct with acute cholecystitis with obstruction
Biliary disease	ICD-10	K80.44	Calculus of bile duct with chronic cholecystitis without obstruction
Biliary disease	ICD-10	K80.45	Calculus of bile duct with chronic cholecystitis with obstruction
Biliary disease	ICD-10	K80.46	Calculus of bile duct with acute and chronic cholecystitis without obstruction
Biliary disease	ICD-10	K80.47	Calculus of bile duct with acute and chronic cholecystitis with obstruction
Biliary disease	ICD-10	K80.5	Calculus of bile duct without cholangitis or cholecystitis
Biliary disease	ICD-10	K80.50	Calculus of bile duct without cholangitis or cholecystitis without obstruction
Biliary disease	ICD-10	K80.51	Calculus of bile duct without cholangitis or cholecystitis with obstruction
Biliary disease	ICD-10	K80.6	Calculus of gallbladder and bile duct with cholecystitis
Biliary disease	ICD-10	K80.60	Calculus of gallbladder and bile duct with cholecystitis unspecified, without obstruction
Biliary disease	ICD-10	K80.61	Calculus of gallbladder and bile duct with cholecystitis unspecified, with obstruction
Biliary disease	ICD-10	K80.62	Calculus of gallbladder and bile duct with acute cholecystitis without obstruction
Biliary disease	ICD-10	K80.63	Calculus of gallbladder and bile duct with acute cholecystitis with obstruction
Biliary disease	ICD-10	K80.64	Calculus of gallbladder and bile duct with chronic cholecystitis without obstruction
Biliary disease	ICD-10	K80.65	Calculus of gallbladder and bile duct with chronic cholecystitis with obstruction
Biliary disease	ICD-10	K80.66	Calculus of gallbladder and bile duct with acute and chronic cholecystitis without obstruction
Biliary disease	ICD-10	K80.67	Calculus of gallbladder and bile duct with acute and chronic cholecystitis with obstruction
Biliary disease	ICD-10	K80.7	Calculus of gallbladder and bile duct without cholecystitis
Biliary disease	ICD-10	K80.70	Calculus of gallbladder and bile duct without cholecystitis without obstruction
Biliary disease	ICD-10	K80.71	Calculus of gallbladder and bile duct without cholecystitis with obstruction
Biliary disease	ICD-10	K80.8	Other cholelithiasis
Biliary disease	ICD-10	K80.80	Other cholelithiasis without obstruction
Biliary disease	ICD-10	K80.81	Other cholelithiasis with obstruction
Biliary disease	ICD-10	K81	Cholecystitis
Biliary disease	ICD-10	K81.0	Acute cholecystitis
Biliary disease	ICD-10	K81.1	Chronic cholecystitis
Biliary disease	ICD-10	K81.2	Acute cholecystitis with chronic cholecystitis
Biliary disease	ICD-10	K81.9	Cholecystitis, unspecified
Biliary disease	ICD-10	K82	Other diseases of gallbladder
Biliary disease	ICD-10	K82.0	Obstruction of gallbladder
Biliary disease	ICD-10	K82.1	Hydrops of gallbladder
Biliary disease	ICD-10	K82.2	Perforation of gallbladder

Type of Liver/Biliary Disease	Code Type	Code	Description
Biliary disease	ICD-10	K82.3	Fistula of gallbladder
Biliary disease	ICD-10	K82.8	Other specified diseases of gallbladder
Biliary disease	ICD-10	K82.9	Disease of gallbladder, unspecified
Biliary disease	ICD-10	K82.A	Disorders of gallbladder in diseases classified elsewhere
Biliary disease	ICD-10	K82.A1	Gangrene of gallbladder in cholecystitis
Biliary disease	ICD-10	K82.A2	Perforation of gallbladder in cholecystitis
Biliary disease	ICD-10	K83.0	Cholangitis
Biliary disease	ICD-10	K83.09	Other cholangitis
Biliary disease	ICD-10	K83.1	Obstruction of bile duct
Biliary disease	ICD-10	K83.2	Perforation of bile duct
Biliary disease	ICD-10	K83.3	Fistula of bile duct
Biliary disease	ICD-10	K83.5	Biliary cyst
Biliary disease	ICD-10	K83.8	Other specified diseases of biliary tract
Biliary disease	ICD-10	K83.9	Disease of biliary tract, unspecified
Biliary disease	ICD-10	K87	Disorders of gallbladder, biliary tract and pancreas in diseases classified elsewhere
Cancer in the liver or biliary tree	ICD-9	155	Malignant neoplasm of liver and intrahepatic bile ducts
Cancer in the liver or biliary tree	ICD-9	155.0	Malignant neoplasm of liver, primary
Cancer in the liver or biliary tree	ICD-9	155.1	Malignant neoplasm of intrahepatic bile ducts
Cancer in the liver or biliary tree	ICD-9	155.2	Malignant neoplasm liver, not specified as primary or secondary
Cancer in the liver or biliary tree	ICD-9	156	Malignant neoplasm of gallbladder and extrahepatic bile ducts
Cancer in the liver or biliary tree	ICD-9	156.0	Malignant neoplasm of gallbladder
Cancer in the liver or biliary tree	ICD-9	156.1	Malignant neoplasm of extrahepatic bile ducts
Cancer in the liver or biliary tree	ICD-9	156.2	Malignant neoplasm of ampulla of Vater
Cancer in the liver or biliary tree	ICD-9	156.8	Malignant neoplasm of other specified sites of gallbladder and extrahepatic bile ducts
Cancer in the liver or biliary tree	ICD-9	156.9	Malignant neoplasm of biliary tract, part unspecified site
Cancer in the liver or biliary tree	ICD-9	197.7	Malignant neoplasm of liver, secondary
Cancer in the liver or biliary tree	ICD-9	209.72	Secondary neuroendocrine tumor of liver
Cancer in the liver or biliary tree	ICD-9	230.8	Carcinoma in situ of liver and biliary system
Cancer in the liver or biliary tree	ICD-10	C22	Malignant neoplasm of liver and intrahepatic bile ducts
Cancer in the liver or biliary tree	ICD-10	C22.0	Liver cell carcinoma
Cancer in the liver or biliary tree	ICD-10	C22.1	Intrahepatic bile duct carcinoma
Cancer in the liver or biliary tree	ICD-10	C22.2	Hepatoblastoma
Cancer in the liver or biliary tree	ICD-10	C22.3	Angiosarcoma of liver
Cancer in the liver or biliary tree	ICD-10	C22.4	Other sarcomas of liver

Type of Liver/Biliary Disease	Code Type	Code	Description
Cancer in the liver or biliary tree	ICD-10	C22.7	Other specified carcinomas of liver
Cancer in the liver or biliary tree	ICD-10	C22.8	Malignant neoplasm of liver, primary, unspecified as to type
Cancer in the liver or biliary tree	ICD-10	C22.9	Malignant neoplasm liver not specified as primary/secondary
Cancer in the liver or biliary tree	ICD-10	C23	Malignant neoplasm of gallbladder
Cancer in the liver or biliary tree	ICD-10	C24	Malignant neoplasm of other and unspecified parts of biliary tract
Cancer in the liver or biliary tree	ICD-10	C24.0	Malignant neoplasm of extrahepatic bile duct
Cancer in the liver or biliary tree	ICD-10	C24.1	Malignant neoplasm of ampulla of Vater
Cancer in the liver or biliary tree	ICD-10	C24.8	Malignant neoplasm of overlapping sites of biliary tract
Cancer in the liver or biliary tree	ICD-10	C24.9	Malignant neoplasm of biliary tract, unspecified
Cancer in the liver or biliary tree	ICD-10	C78.7	Secondary malignant neoplasm of liver and intrahepatic bile duct
Cancer in the liver or biliary tree	ICD-10	C7B.02	Secondary carcinoid tumors of liver
Cancer in the liver or biliary tree	ICD-10	D01.5	Carcinoma in situ of liver, gallbladder and bile ducts
Cirrhosis (compensated or decompensated)	ICD-9	456.0	Esophageal varices with bleeding
Cirrhosis (compensated or decompensated)	ICD-9	456.1	Esophageal varices without bleeding
Cirrhosis (compensated or decompensated)	ICD-9	456.2	Esophageal varices in diseases classified elsewhere
Cirrhosis (compensated or decompensated)	ICD-9	456.20	Esophageal varices in diseases classified elsewhere with bleeding
Cirrhosis (compensated or decompensated)	ICD-9	456.21	Esophageal varices in diseases classified elsewhere without bleeding
Cirrhosis (compensated or decompensated)	ICD-9	567.23	Spontaneous bacterial peritonitis
Cirrhosis (compensated or decompensated)	ICD-9	571.5	Cirrhosis of liver without mention of alcohol
Cirrhosis (compensated or decompensated)	ICD-9	572.2	Hepatic encephalopathy
Cirrhosis (compensated or decompensated)	ICD-9	572.3	Portal hypertension
Cirrhosis (compensated or decompensated)	ICD-9	572.4	Hepatorenal syndrome
Cirrhosis (compensated or decompensated)	ICD-9	789.5	Ascites
Cirrhosis (compensated or decompensated)	ICD-9	789.59	Other ascites
Cirrhosis (compensated or decompensated)	ICD-10	I85	Esophageal varices
Cirrhosis (compensated or decompensated)	ICD-10	I85.0	Esophageal varices
Cirrhosis (compensated or decompensated)	ICD-10	I85.00	Esophageal varices without bleeding
Cirrhosis (compensated or decompensated)	ICD-10	I85.01	Esophageal varices with bleeding
Cirrhosis (compensated or decompensated)	ICD-10	I85.1	Secondary esophageal varices
Cirrhosis (compensated or decompensated)	ICD-10	I85.10	Secondary esophageal varices without bleeding
Cirrhosis (compensated or decompensated)	ICD-10	I85.11	Secondary esophageal varices with bleeding
Cirrhosis (compensated or decompensated)	ICD-10	K65.2	Spontaneous bacterial peritonitis
Cirrhosis (compensated or decompensated)	ICD-10	K71.51	Toxic liver disease with chronic active hepatitis with ascites
Cirrhosis (compensated or decompensated)	ICD-10	K72.1	Chronic hepatic failure

Type of Liver/Biliary Disease	Code Type	Code	Description
Cirrhosis (compensated or decompensated)	ICD-10	K72.10	Chronic hepatic failure without coma
Cirrhosis (compensated or decompensated)	ICD-10	K72.11	Chronic hepatic failure with coma
Cirrhosis (compensated or decompensated)	ICD-10	K72.9	Hepatic failure, unspecified
Cirrhosis (compensated or decompensated)	ICD-10	K72.90	Hepatic failure, unspecified without coma
Cirrhosis (compensated or decompensated)	ICD-10	K72.91	Hepatic failure, unspecified with coma
Cirrhosis (compensated or decompensated)	ICD-10	K74.1	Hepatic sclerosis
Cirrhosis (compensated or decompensated)	ICD-10	K74.2	Hepatic fibrosis with hepatic sclerosis
Cirrhosis (compensated or decompensated)	ICD-10	K74.60	Unspecified cirrhosis of liver
Cirrhosis (compensated or decompensated)	ICD-10	K76.7	Hepatorenal syndrome
Cirrhosis (compensated or decompensated)	ICD-10	K76.81	Hepatopulmonary syndrome
Cirrhosis (compensated or decompensated)	ICD-10	R18.8	Other ascites
Congestive hepatopathy	ICD-9	573.0	Chronic passive congestion of the liver
Congestive hepatopathy	ICD-10	K76.1	Chronic passive congestion of the liver
Hemochromatosis	ICD-9	275.01	Hereditary hemochromatosis
Hemochromatosis	ICD-9	275.02	Hemochromatosis due to repeated red blood cell transfusions
Hemochromatosis	ICD-9	275.03	Other hemochromatosis
Hemochromatosis	ICD-10	E83.110	Hereditary hemochromatosis
Hepatitis A	ICD-9	070.0	Viral hepatitis A with hepatic coma
Hepatitis A	ICD-9	070.1	Viral hepatitis A without mention of hepatic coma
Hepatitis A	ICD-10	B15	Acute hepatitis A
Hepatitis A	ICD-10	B15.0	Hepatitis A with hepatic coma
Hepatitis A	ICD-10	B15.9	Hepatitis A without hepatic coma
Hepatitis B	ICD-9	070.2	Viral hepatitis B with hepatic coma
Hepatitis B	ICD-9	070.20	Viral hepatitis B with hepatic coma, acute or unspecified, without mention of hepatitis delta
Hepatitis B	ICD-9	070.22	Chronic viral hepatitis B with hepatic coma without mention of hepatitis delta
Hepatitis B	ICD-9	070.3	Viral hepatitis B without mention of hepatic coma
Hepatitis B	ICD-9	070.30	Viral hepatitis B without mention of hepatic coma, acute or unspecified, without mention of hepatitis delta
Hepatitis B	ICD-9	070.32	Chronic viral hepatitis B without mention of hepatic coma without mention of hepatitis delta
Hepatitis B	ICD-9	V02.61	Hepatitis B Carrier
Hepatitis B	ICD-10	B16	Acute hepatitis B
Hepatitis B	ICD-10	B16.2	Acute hepatitis B without delta-agent with hepatic coma
Hepatitis B	ICD-10	B16.9	Acute hepatitis B without delta-agent and without hepatic coma
Hepatitis B	ICD-10	B18.1	Chronic viral hepatitis B without delta-agent

Type of Liver/Biliary Disease	Code Type	Code	Description
Hepatitis B	ICD-10	B19.1	Unspecified viral hepatitis B
Hepatitis B	ICD-10	B19.10	Unspecified viral hepatitis B without hepatic coma
Hepatitis B	ICD-10	B19.11	Unspecified viral hepatitis B with hepatic coma
Hepatitis B and D	ICD-9	070.21	Viral hepatitis B with hepatic coma, acute or unspecified, with hepatitis delta
Hepatitis B and D	ICD-9	070.23	Chronic viral hepatitis B with hepatic coma with hepatitis delta
Hepatitis B and D	ICD-9	070.31	Viral hepatitis B without mention of hepatic coma, acute or unspecified, with hepatitis delta
Hepatitis B and D	ICD-9	070.33	Chronic viral hepatitis B without mention of hepatic coma with mention of hepatitis delta
Hepatitis B and D	ICD-9	070.42	Hepatitis delta without mention of active hepatitis B disease with hepatic coma
Hepatitis B and D	ICD-9	070.52	Hepatitis delta without mention of active hepatitis B disease or hepatic coma
Hepatitis B and D	ICD-10	B16.0	Acute hepatitis B with delta-agent with hepatic coma
Hepatitis B and D	ICD-10	B16.1	Acute hepatitis B with delta-agent without hepatic coma
Hepatitis B and D	ICD-10	B17.0	Acute delta-(super) infection of hepatitis B carrier
Hepatitis B and D	ICD-10	B18.0	Chronic viral hepatitis B with delta-agent
Hepatitis C	ICD-9	070.41	Acute hepatitis C with hepatic coma
Hepatitis C	ICD-9	070.44	Chronic hepatitis C with hepatic coma
Hepatitis C	ICD-9	070.51	Acute hepatitis C without mention of hepatic coma
Hepatitis C	ICD-9	070.54	Chronic hepatitis C without mention of hepatic coma
Hepatitis C	ICD-9	070.7	Unspecified viral hepatitis C
Hepatitis C	ICD-9	070.70	Unspecified viral hepatitis C without hepatic coma
Hepatitis C	ICD-9	070.71	Unspecified viral hepatitis C with hepatic coma
Hepatitis C	ICD-9	V02.62	Hepatitis C carrier
Hepatitis C	ICD-10	B17.1	Acute hepatitis C
Hepatitis C	ICD-10	B17.10	Acute hepatitis C without hepatic coma
Hepatitis C	ICD-10	B17.11	Acute hepatitis C with hepatic coma
Hepatitis C	ICD-10	B18.2	Chronic viral hepatitis C
Hepatitis C	ICD-10	B19.2	Unspecified viral hepatitis C
Hepatitis C	ICD-10	B19.20	Unspecified viral hepatitis C without hepatic coma
Hepatitis C	ICD-10	B19.21	Unspecified viral hepatitis C with hepatic coma
Hepatitis E	ICD-9	070.43	Hepatitis E with hepatic coma
Hepatitis E	ICD-9	070.53	Hepatitis E without mention of hepatic coma
Hepatitis E	ICD-10	B17.2	Acute hepatitis E
Metabolic dysfunction-associated steatotic liver disease	ICD-9	571.8	Other chronic non-alcoholic liver disease

Type of Liver/Biliary Disease	Code Type	Code	Description
Metabolic dysfunction-associated steatotic liver disease	ICD-9	571.9	Unspecified chronic liver disease without mention of alcohol
Metabolic dysfunction-associated steatotic liver disease	ICD-10	K76.0	Fatty change of liver, not elsewhere classified
Other acute or unspecified viral hepatitis	ICD-9	070	Viral hepatitis
Other acute or unspecified viral hepatitis	ICD-9	070.4	Other specified viral hepatitis with hepatic coma
Other acute or unspecified viral hepatitis	ICD-9	070.49	Other specified viral hepatitis with hepatic coma
Other acute or unspecified viral hepatitis	ICD-9	070.5	Other specified viral hepatitis without mention of hepatic coma
Other acute or unspecified viral hepatitis	ICD-9	070.59	Other specified viral hepatitis without mention of hepatic coma
Other acute or unspecified viral hepatitis	ICD-9	070.6	Unspecified viral hepatitis with hepatic coma
Other acute or unspecified viral hepatitis	ICD-9	070.9	Unspecified viral hepatitis without mention of hepatic coma
Other acute or unspecified viral hepatitis	ICD-9	072.71	Mumps hepatitis
Other acute or unspecified viral hepatitis	ICD-9	573.1	Hepatitis in viral diseases classified elsewhere
Other acute or unspecified viral hepatitis	ICD-9	V02.69	Other viral hepatitis carrier
Other acute or unspecified viral hepatitis	ICD-10	B00.81	Herpesviral hepatitis
Other acute or unspecified viral hepatitis	ICD-10	B17	Other acute viral hepatitis
Other acute or unspecified viral hepatitis	ICD-10	B17.8	Other specified acute viral hepatitis
Other acute or unspecified viral hepatitis	ICD-10	B17.9	Acute viral hepatitis, unspecified
Other acute or unspecified viral hepatitis	ICD-10	B18	Chronic viral hepatitis
Other acute or unspecified viral hepatitis	ICD-10	B18.8	Other chronic viral hepatitis
Other acute or unspecified viral hepatitis	ICD-10	B18.9	Chronic viral hepatitis, unspecified
Other acute or unspecified viral hepatitis	ICD-10	B19	Unspecified viral hepatitis
Other acute or unspecified viral hepatitis	ICD-10	B19.0	Unspecified viral hepatitis with hepatic coma
Other acute or unspecified viral hepatitis	ICD-10	B19.9	Unspecified viral hepatitis without hepatic coma
Other acute or unspecified viral hepatitis	ICD-10	B25.1	Cytomegaloviral hepatitis
Other acute or unspecified viral hepatitis	ICD-10	B26.81	Mumps hepatitis
Other acute or unspecified viral hepatitis	ICD-10	B94.2	Sequelae of viral hepatitis
Other acute or unspecified viral hepatitis	ICD-10	O98.4	Viral hepatitis complicating pregnancy, childbirth and the puerperium
Other acute or unspecified viral hepatitis	ICD-10	O98.41	Viral hepatitis complicating pregnancy
Other acute or unspecified viral hepatitis	ICD-10	O98.411	Viral hepatitis complicating pregnancy, first trimester
Other acute or unspecified viral hepatitis	ICD-10	O98.412	Viral hepatitis complicating pregnancy, second trimester
Other acute or unspecified viral hepatitis	ICD-10	O98.413	Viral hepatitis complicating pregnancy, third trimester
Other acute or unspecified viral hepatitis	ICD-10	O98.419	Viral hepatitis complicating pregnancy, unspecified trimester
Other acute or unspecified viral hepatitis	ICD-10	O98.42	Viral hepatitis complicating childbirth
Other acute or unspecified viral hepatitis	ICD-10	O98.43	Viral hepatitis complicating the puerperium

Type of Liver/Biliary Disease	Code Type	Code	Description
Other acute or unspecified viral hepatitis	ICD-10	P35.3	Congenital viral hepatitis
Other unspecified hepatitis/liver disease	ICD-9	091.62	Secondary syphilitic hepatitis
Other unspecified hepatitis/liver disease	ICD-9	130.5	Hepatitis due to toxoplasmosis
Other unspecified hepatitis/liver disease	ICD-9	571	Chronic liver disease and cirrhosis
Other unspecified hepatitis/liver disease	ICD-9	571.4	Chronic hepatitis
Other unspecified hepatitis/liver disease	ICD-9	571.40	Chronic hepatitis, unspecified
Other unspecified hepatitis/liver disease	ICD-9	571.41	Chronic persistent hepatitis
Other unspecified hepatitis/liver disease	ICD-9	571.49	Other chronic hepatitis
Other unspecified hepatitis/liver disease	ICD-9	573.2	Hepatitis in other infectious diseases classified elsewhere
Other unspecified hepatitis/liver disease	ICD-9	573.3[a]	Hepatitis, unspecified
Other unspecified hepatitis/liver disease	ICD-9	573.4	Hepatic infarction
Other unspecified hepatitis/liver disease	ICD-9	573.8[a]	Other specified disorder of liver
Other unspecified hepatitis/liver disease	ICD-9	573.9[a]	Unspecified disorder of liver
Other unspecified hepatitis/liver disease	ICD-10	A51.45	Secondary syphilitic hepatitis
Other unspecified hepatitis/liver disease	ICD-10	B58.1	Toxoplasma hepatitis
Other unspecified hepatitis/liver disease	ICD-10	K73	Chronic hepatitis, not elsewhere classified
Other unspecified hepatitis/liver disease	ICD-10	K73.0	Chronic persistent hepatitis, not elsewhere classified
Other unspecified hepatitis/liver disease	ICD-10	K73.1	Chronic lobular hepatitis, not elsewhere classified
Other unspecified hepatitis/liver disease	ICD-10	K73.2	Chronic active hepatitis, not elsewhere classified
Other unspecified hepatitis/liver disease	ICD-10	K73.8	Other chronic hepatitis, not elsewhere classified
Other unspecified hepatitis/liver disease	ICD-10	K73.9	Chronic hepatitis, unspecified
Other unspecified hepatitis/liver disease	ICD-10	K76.89[a]	Other specified diseases of liver
Other unspecified hepatitis/liver disease	ICD-10	K76.9[a]	Liver disease, unspecified
Primary sclerosing cholangitis	ICD-10	K83.01	Primary sclerosing cholangitis
Primary/secondary biliary cirrhosis	ICD-9	571.6	Biliary cirrhosis
Primary/secondary biliary cirrhosis	ICD-10	K74.3	Primary biliary cirrhosis
Primary/secondary biliary cirrhosis	ICD-10	K74.4	Secondary biliary cirrhosis
Primary/secondary biliary cirrhosis	ICD-10	K74.5	Biliary cirrhosis, unspecified
Wilson's disease	ICD-9	275.1	Disorders of copper metabolism
Wilson's disease	ICD-10	E83.01	Wilson's disease

Abbreviations: ICD-9=International Classification of Diseases, Ninth Revision; ICD-10=International Classification of Diseases, Tenth Revision

[a] Diagnostic code was not used for censoring because of the potential for clinicians to record drug-induced liver injury.

eTable 3. List of International Classification of Diseases, Ninth Revision (ICD-9) and International Classification of Diseases, Tenth Revision (ICD-10) diagnostic codes used to identify certain conditions that could precipitate findings consistent with severe acute livery injury for exclusion and censoring.

Type of Non-Drug-Related Condition	Code Type	Code	Description
Acquired hemolytic anemia	ICD-9	283.1	Non-autoimmune hemolytic anemia
Acquired hemolytic anemia	ICD-9	283.10	Non-autoimmune hemolytic anemia, unspecified
Acquired hemolytic anemia	ICD-9	283.11	Hemolytic uremic syndrome
Acquired hemolytic anemia	ICD-9	283.19	Other non-autoimmune hemolytic anemias
Acquired hemolytic anemia	ICD-9	283.2	Hemoglobinuria due to hemolysis from external causes
Acquired hemolytic anemia	ICD-9	283.9	Acquired hemolytic anemia, unspecified
Acquired hemolytic anemia	ICD-10	D59.1	Other autoimmune hemolytic anemias
Acquired hemolytic anemia	ICD-10	D59.10	Autoimmune hemolytic anemia, unspecified
Acquired hemolytic anemia	ICD-10	D59.11	Warm autoimmune hemolytic anemia
Acquired hemolytic anemia	ICD-10	D59.12	Cold autoimmune hemolytic anemia
Acquired hemolytic anemia	ICD-10	D59.13	Mixed type autoimmune hemolytic anemia
Acquired hemolytic anemia	ICD-10	D59.19	Other autoimmune hemolytic anemia
Acquired hemolytic anemia	ICD-10	D59.3	Hemolytic-uremic syndrome
Acquired hemolytic anemia	ICD-10	D59.30	Hemolytic-uremic syndrome, unspecified
Acquired hemolytic anemia	ICD-10	D59.31	Infection-associated hemolytic-uremic syndrome
Acquired hemolytic anemia	ICD-10	D59.32	Hereditary hemolytic-uremic syndrome
Acquired hemolytic anemia	ICD-10	D59.39	Other hemolytic-uremic syndrome
Acquired hemolytic anemia	ICD-10	D59.4	Other nonautoimmune hemolytic anemias
Acquired hemolytic anemia	ICD-10	D59.5	Paroxysmal nocturnal hemoglobinuria [Marchiafava-Micheli]
Acquired hemolytic anemia	ICD-10	D59.6	Hemoglobinuria due to hemolysis from other external causes
Acquired hemolytic anemia	ICD-10	D59.8	Other acquired hemolytic anemias
Acquired hemolytic anemia	ICD-10	D59.9	Acquired hemolytic anemia, unspecified
Alcohol dependence	ICD-9	291.1	Alcohol-induced persisting amnestic disorder
Alcohol dependence	ICD-9	291.2	Alcohol-induced persisting dementia
Alcohol dependence	ICD-9	291.3	Alcohol-induced psychotic disorder with hallucinations
Alcohol dependence	ICD-9	291.5	Alcohol-induced psychotic disorder with delusions
Alcohol dependence	ICD-9	291.82	Alcohol induced sleep disorders
Alcohol dependence	ICD-9	291.89	Other alcohol-induced mental disorders
Alcohol dependence	ICD-9	291.9	Unspecified alcohol-induced mental disorders
Alcohol dependence	ICD-9	303.00	Acute alcoholic intoxication in alcoholism, unspecified
Alcohol dependence	ICD-9	303.01	Acute alcoholic intoxication in alcoholism, continuous
Alcohol dependence	ICD-9	303.02	Acute alcoholic intoxication in alcoholism, episodic

Type of Non-Drug-Related Condition	Code Type	Code	Description
Alcohol dependence	ICD-9	303.90	Other and unspecified alcohol dependence, unspecified
Alcohol dependence	ICD-9	303.91	Other and unspecified alcohol dependence, continuous
Alcohol dependence	ICD-9	303.92	Other and unspecified alcohol dependence, episodic
Alcohol dependence	ICD-10	F10.20	Alcohol dependence, uncomplicated
Alcohol dependence	ICD-10	F10.22	Alcohol dependence with intoxication
Alcohol dependence	ICD-10	F10.220	Alcohol dependence with intoxication, uncomplicated
Alcohol dependence	ICD-10	F10.221	Alcohol dependence with intoxication delirium
Alcohol dependence	ICD-10	F10.229	Alcohol dependence with intoxication, unspecified
Alcohol dependence	ICD-10	F10.24	Alcohol dependence with alcohol-induced mood disorder
Alcohol dependence	ICD-10	F10.25	Alcohol dependence with alcohol-induced psychotic disorder
Alcohol dependence	ICD-10	F10.250	Alcohol dependence with alcohol-induced psychotic disorder with delusions
Alcohol dependence	ICD-10	F10.251	Alcohol dependence with alcohol-induced psychotic disorder with hallucinations
Alcohol dependence	ICD-10	F10.259	Alcohol dependence with alcohol-induced psychotic disorder, unspecified
Alcohol dependence	ICD-10	F10.26	Alcohol dependence with alcohol-induced persisting amnestic disorder
Alcohol dependence	ICD-10	F10.27	Alcohol dependence with alcohol-induced persisting dementia
Alcohol dependence	ICD-10	F10.28	Alcohol dependence with other alcohol-induced disorders
Alcohol dependence	ICD-10	F10.280	Alcohol dependence with alcohol-induced anxiety disorder
Alcohol dependence	ICD-10	F10.281	Alcohol dependence with alcohol-induced sexual dysfunction
Alcohol dependence	ICD-10	F10.282	Alcohol dependence with alcohol-induced sleep disorder
Alcohol dependence	ICD-10	F10.288	Alcohol dependence with other alcohol-induced disorder
Alcohol dependence	ICD-10	F10.29	Alcohol dependence with unspecified alcohol-induced disorder
Alcohol withdrawal with abuse/dependence	ICD-9	291.0	Alcohol withdrawal delirium
Alcohol withdrawal with abuse/dependence	ICD-9	291.81	Alcohol withdrawal
Alcohol withdrawal with abuse/dependence	ICD-10	F10.13	Alcohol abuse with withdrawal
Alcohol withdrawal with abuse/dependence	ICD-10	F10.130	Alcohol abuse with withdrawal, uncomplicated
Alcohol withdrawal with abuse/dependence	ICD-10	F10.131	Alcohol abuse with withdrawal delirium
Alcohol withdrawal with abuse/dependence	ICD-10	F10.132	Alcohol abuse with withdrawal with perceptual disturbance
Alcohol withdrawal with abuse/dependence	ICD-10	F10.139	Alcohol abuse with withdrawal, unspecified
Alcohol withdrawal with abuse/dependence	ICD-10	F10.23	Alcohol dependence with withdrawal
Alcohol withdrawal with abuse/dependence	ICD-10	F10.230	Alcohol dependence with withdrawal, uncomplicated
Alcohol withdrawal with abuse/dependence	ICD-10	F10.231	Alcohol dependence with withdrawal delirium
Alcohol withdrawal with abuse/dependence	ICD-10	F10.232	Alcohol dependence with withdrawal with perceptual disturbance
Alcohol withdrawal with abuse/dependence	ICD-10	F10.239	Alcohol dependence with withdrawal, unspecified
Alcohol withdrawal with abuse/dependence	ICD-10	F10.93	Alcohol use, unspecified with withdrawal
Alcohol withdrawal with abuse/dependence	ICD-10	F10.930	Alcohol use, unspecified with withdrawal, uncomplicated
Alcohol withdrawal with abuse/dependence	ICD-10	F10.931	Alcohol use, unspecified with withdrawal delirium

Type of Non-Drug-Related Condition	Code Type	Code	Description
Alcohol withdrawal with abuse/dependence	ICD-10	F10.932	Alcohol use, unspecified with withdrawal with perceptual disturbance
Alcohol withdrawal with abuse/dependence	ICD-10	F10.939	Alcohol use, unspecified with withdrawal, unspecified
Anemia in chronic diseases	ICD-9	285.2	Anemia of chronic illness
Anemia in chronic diseases	ICD-9	285.21	Anemia in chronic kidney disease
Anemia in chronic diseases	ICD-9	285.22	Anemia in neoplastic disease
Anemia in chronic diseases	ICD-9	285.29	Anemia in other chronic disease
Anemia in chronic diseases	ICD-10	D63	Anemia in chronic diseases classified elsewhere
Anemia in chronic diseases	ICD-10	D63.0	Anemia in neoplastic disease
Anemia in chronic diseases	ICD-10	D63.1	Anemia in chronic kidney disease
Anemia in chronic diseases	ICD-10	D63.8	Anemia in other chronic diseases classified elsewhere
Anemia due to enzyme disorders	ICD-9	282.2	Anemias due to disorders of glutathione metabolism
Anemia due to enzyme disorders	ICD-9	282.3	Other hemolytic anemias due to enzyme deficiency
Anemia due to enzyme disorders	ICD-10	D55	Anemia due to enzyme disorders
Anemia due to enzyme disorders	ICD-10	D55.0	Anemia due to glucose-6-phosphate dehydrogenase [G6PD] deficiency
Anemia due to enzyme disorders	ICD-10	D55.1	Anemia due to other disorders of glutathione metabolism
Anemia due to enzyme disorders	ICD-10	D55.2	Anemia due to disorders of glycolytic enzymes
Anemia due to enzyme disorders	ICD-10	D55.21	Anemia due to pyruvate kinase deficiency
Anemia due to enzyme disorders	ICD-10	D55.29	Anemia due to other disorders of glycolytic enzymes
Anemia due to enzyme disorders	ICD-10	D55.3	Anemia due to disorders of nucleotide metabolism
Anemia due to enzyme disorders	ICD-10	D55.8	Other anemias due to enzyme disorders
Anemia due to enzyme disorders	ICD-10	D55.9	Anemia due to enzyme disorder, unspecified
Budd Chiari Syndrome	ICD-9	453.0	Budd-Chiari syndrome
Budd Chiari Syndrome	ICD-10	I82.0	Budd Chiari syndrome
Cancer of the pancreas	ICD-9	157	Malignant neoplasm of pancreas
Cancer of the pancreas	ICD-9	157.0	Malignant neoplasm of head of pancreas
Cancer of the pancreas	ICD-9	157.1	Malignant neoplasm of body of pancreas
Cancer of the pancreas	ICD-9	157.2	Malignant neoplasm of tail of pancreas
Cancer of the pancreas	ICD-9	157.3	Malignant neoplasm of pancreatic duct
Cancer of the pancreas	ICD-9	157.4	Malignant neoplasm of islets of Langerhans
Cancer of the pancreas	ICD-9	157.8	Malignant neoplasm of other specified sites of pancreas
Cancer of the pancreas	ICD-9	157.9	Malignant neoplasm of pancreas, part unspecified
Cancer of the pancreas	ICD-10	C25	Malignant neoplasm of pancreas
Cancer of the pancreas	ICD-10	C25.0	Malignant neoplasm of head of pancreas
Cancer of the pancreas	ICD-10	C25.1	Malignant neoplasm of body of pancreas
Cancer of the pancreas	ICD-10	C25.2	Malignant neoplasm of tail of pancreas

Type of Non-Drug-Related Condition	Code Type	Code	Description
Cancer of the pancreas	ICD-10	C25.3	Malignant neoplasm of pancreatic duct
Cancer of the pancreas	ICD-10	C25.4	Malignant neoplasm of endocrine pancreas
Cancer of the pancreas	ICD-10	C25.7	Malignant neoplasm of other parts of pancreas
Cancer of the pancreas	ICD-10	C25.8	Malignant neoplasm of overlapping sites of pancreas
Cancer of the pancreas	ICD-10	C25.9	Malignant neoplasm of pancreas, unspecified
Celiac disease	ICD-9	579.0	Celiac disease
Celiac disease	ICD-10	K90.0	Celiac disease
Hypotension	ICD-9	458	Hypotension
Hypotension	ICD-9	458.0	Orthostatic hypotension
Hypotension	ICD-9	458.1	Chronic hypotension
Hypotension	ICD-9	458.2	Iatrogenic hypotension
Hypotension	ICD-9	458.21	Hypotension of hemodialysis
Hypotension	ICD-9	458.29	Other iatrogenic hypotension
Hypotension	ICD-9	458.8	Other specified hypotension
Hypotension	ICD-9	458.9	Hypotension, unspecified
Hypotension	ICD-10	I95	Hypotension
Hypotension	ICD-10	I95.0	Idiopathic hypotension
Hypotension	ICD-10	I95.1	Orthostatic hypotension
Hypotension	ICD-10	I95.2	Hypotension due to drugs
Hypotension	ICD-10	I95.3	Hypotension of hemodialysis
Hypotension	ICD-10	I95.8	Other hypotension
Hypotension	ICD-10	I95.81	Postprocedural hypotension
Hypotension	ICD-10	I95.89	Other hypotension
Hypotension	ICD-10	I95.9	Hypotension, unspecified
Other hereditary hemolytic anemias	ICD-9	282	Hereditary hemolytic anemias
Other hereditary hemolytic anemias	ICD-9	282.0	Hereditary spherocytosis
Other hereditary hemolytic anemias	ICD-9	282.1	Hereditary elliptocytosis
Other hereditary hemolytic anemias	ICD-9	282.7	Other hemoglobinopathies
Other hereditary hemolytic anemias	ICD-9	282.8	Other specified hereditary hemolytic anemias
Other hereditary hemolytic anemias	ICD-9	282.9	Hereditary hemolytic anemia, unspecified
Other hereditary hemolytic anemias	ICD-10	D58	Other hereditary hemolytic anemias
Other hereditary hemolytic anemias	ICD-10	D58.0	Hereditary spherocytosis
Other hereditary hemolytic anemias	ICD-10	D58.1	Hereditary elliptocytosis
Other hereditary hemolytic anemias	ICD-10	D58.2	Other hemoglobinopathies
Other hereditary hemolytic anemias	ICD-10	D58.8	Other specified hereditary hemolytic anemias

Type of Non-Drug-Related Condition	Code Type	Code	Description
Other hereditary hemolytic anemias	ICD-10	D58.9	Hereditary hemolytic anemia, unspecified
Pancreatitis	ICD-9	577	Diseases of pancreas
Pancreatitis	ICD-9	577.0	Acute pancreatitis
Pancreatitis	ICD-9	577.1	Chronic pancreatitis
Pancreatitis	ICD-9	577.2	Cyst and pseudocyst of pancreas
Pancreatitis	ICD-9	577.9	Unspecified disease of pancreas
Pancreatitis	ICD-9	577.8	Other specified diseases of pancreas
Pancreatitis	ICD-10	K85.0	Idiopathic acute pancreatitis
Pancreatitis	ICD-10	K85.00	Idiopathic acute pancreatitis without necrosis or infection
Pancreatitis	ICD-10	K85.01	Idiopathic acute pancreatitis with uninfected necrosis
Pancreatitis	ICD-10	K85.02	Idiopathic acute pancreatitis with infected necrosis
Pancreatitis	ICD-10	K85.1	Biliary acute pancreatitis
Pancreatitis	ICD-10	K85.10	Biliary acute pancreatitis without necrosis or infection
Pancreatitis	ICD-10	K85.11	Biliary acute pancreatitis with uninfected necrosis
Pancreatitis	ICD-10	K85.12	Biliary acute pancreatitis with infected necrosis
Pancreatitis	ICD-10	K85.2	Alcohol induced acute pancreatitis
Pancreatitis	ICD-10	K85.20	Alcohol induced acute pancreatitis without necrosis or infection
Pancreatitis	ICD-10	K85.21	Alcohol induced acute pancreatitis with uninfected necrosis
Pancreatitis	ICD-10	K85.22	Alcohol induced acute pancreatitis with infected necrosis
Pancreatitis	ICD-10	K85.8	Other acute pancreatitis
Pancreatitis	ICD-10	K85.80	Other acute pancreatitis without necrosis or infection
Pancreatitis	ICD-10	K85.81	Other acute pancreatitis with uninfected necrosis
Pancreatitis	ICD-10	K85.82	Other acute pancreatitis with infected necrosis
Pancreatitis	ICD-10	K85.9	Acute pancreatitis, unspecified
Pancreatitis	ICD-10	K85.90	Acute pancreatitis without necrosis or infection, unspecified
Pancreatitis	ICD-10	K85.91	Acute pancreatitis with uninfected necrosis, unspecified
Pancreatitis	ICD-10	K85.92	Acute pancreatitis with infected necrosis, unspecified
Pancreatitis	ICD-10	K86	Other diseases of pancreas
Pancreatitis	ICD-10	K86.0	Alcohol-induced chronic pancreatitis
Pancreatitis	ICD-10	K86.1	Other chronic pancreatitis
Pancreatitis	ICD-10	K86.2	Cyst of pancreas
Pancreatitis	ICD-10	K86.3	Pseudocyst of pancreas
Pancreatitis	ICD-10	K86.8	Other specified diseases of pancreas
Pancreatitis	ICD-10	K86.81	Exocrine pancreatic insufficiency
Pancreatitis	ICD-10	K86.89	Other specified diseases of pancreas
Pancreatitis	ICD-10	K86.9	Disease of pancreas, unspecified

Type of Non-Drug-Related Condition	Code Type	Code	Description
Rhabdomyolysis	ICD-9	728.88	Rhabdomyolysis
Rhabdomyolysis	ICD-10	M62.82	Rhabdomyolysis
Rhabdomyolysis	ICD-10	T79.6	Traumatic ischemia of the muscle
Rhabdomyolysis	ICD-10	T79.6XXA	Traumatic ischemia of the muscle initial encounter
Rhabdomyolysis	ICD-10	T79.6XXD	Traumatic ischemia of the muscle subsequent encounter
Rhabdomyolysis	ICD-10	T79.6XXS	Traumatic ischemia of the muscle sequela
Sepsis without shock	ICD-9	003.1	Salmonella septicemia
Sepsis without shock	ICD-9	020.2	Septicemic plague
Sepsis without shock	ICD-9	022.3	Anthrax septicemia
Sepsis without shock	ICD-9	038	Septicemia
Sepsis without shock	ICD-9	038.0	Streptococcal septicemia
Sepsis without shock	ICD-9	038.1	Staphylococcal septicemia
Sepsis without shock	ICD-9	038.10	Staphylococcal septicemia, unspecified
Sepsis without shock	ICD-9	038.11	Methicillin susceptible Staphylococcus aureus septicemia
Sepsis without shock	ICD-9	038.12	Methicillin resistant Staphylococcus aureus septicemia
Sepsis without shock	ICD-9	038.19	Other staphylococcal septicemia
Sepsis without shock	ICD-9	038.2	Pneumococcal septicemia [Streptococcus pneumoniae septicemia]
Sepsis without shock	ICD-9	038.3	Septicemia due to anaerobes
Sepsis without shock	ICD-9	038.4	Septicemia due to other gram-negative organisms
Sepsis without shock	ICD-9	038.40	Septicemia due to gram-negative organism, unspecified
Sepsis without shock	ICD-9	038.41	Septicemia due to hemophilus influenzae [H. influenzae]
Sepsis without shock	ICD-9	038.42	Septicemia due to escherichia coli [E. coli]
Sepsis without shock	ICD-9	038.43	Septicemia due to pseudomonas
Sepsis without shock	ICD-9	038.44	Septicemia due to serratia
Sepsis without shock	ICD-9	038.49	Other septicemia due to gram-negative organisms
Sepsis without shock	ICD-9	038.8	Other specified septicemias
Sepsis without shock	ICD-9	038.9	Unspecified septicemia
Sepsis without shock	ICD-9	670.2	Puerperal sepsis
Sepsis without shock	ICD-9	670.20	Puerperal sepsis, unspecified as to episode of care or not applicable
Sepsis without shock	ICD-9	670.22	Puerperal sepsis, delivered, with mention of postpartum complication
Sepsis without shock	ICD-9	670.24	Puerperal sepsis, postpartum condition or complication
Sepsis without shock	ICD-9	790.7	Bacteremia
Sepsis without shock	ICD-9	995.9	Systemic inflammatory response syndrome (SIRS)
Sepsis without shock	ICD-9	995.90	Systemic inflammatory response syndrome, unspecified
Sepsis without shock	ICD-9	995.91	Sepsis
Sepsis without shock	ICD-9	995.92	Severe sepsis

Type of Non-Drug-Related Condition	Code Type	Code	Description
Sepsis without shock	ICD-9	995.93	Systemic inflammatory response syndrome due to noninfectious process without acute organ dysfunction
Sepsis without shock	ICD-9	995.94	Systemic inflammatory response syndrome due to noninfectious process with acute organ dysfunction
Sepsis without shock	ICD-10	A02.1	Salmonella sepsis
Sepsis without shock	ICD-10	A20.7	Septicemic plague
Sepsis without shock	ICD-10	A22.7	Anthrax sepsis
Sepsis without shock	ICD-10	A26.7	Erysipelothrix sepsis
Sepsis without shock	ICD-10	A32.7	Listerial sepsis
Sepsis without shock	ICD-10	A40	Streptococcal sepsis
Sepsis without shock	ICD-10	A40.0	Sepsis due to streptococcus, group A
Sepsis without shock	ICD-10	A40.1	Sepsis due to streptococcus, group B
Sepsis without shock	ICD-10	A40.3	Sepsis due to Streptococcus pneumoniae
Sepsis without shock	ICD-10	A40.8	Other streptococcal sepsis
Sepsis without shock	ICD-10	A40.9	Streptococcal sepsis, unspecified
Sepsis without shock	ICD-10	A41	Other sepsis
Sepsis without shock	ICD-10	A41.0	Sepsis due to Staphylococcus aureus
Sepsis without shock	ICD-10	A41.01	Sepsis due to Methicillin susceptible Staphylococcus aureus
Sepsis without shock	ICD-10	A41.02	Sepsis due to Methicillin resistant Staphylococcus aureus
Sepsis without shock	ICD-10	A41.1	Sepsis due to other specified staphylococcus
Sepsis without shock	ICD-10	A41.2	Sepsis due to unspecified staphylococcus
Sepsis without shock	ICD-10	A41.3	Sepsis due to Hemophilus influenzae
Sepsis without shock	ICD-10	A41.4	Sepsis due to anaerobes
Sepsis without shock	ICD-10	A41.5	Sepsis due to other Gram-negative organisms
Sepsis without shock	ICD-10	A41.50	Gram-negative sepsis, unspecified
Sepsis without shock	ICD-10	A41.51	Sepsis due to Escherichia coli [E. coli]
Sepsis without shock	ICD-10	A41.52	Sepsis due to Pseudomonas
Sepsis without shock	ICD-10	A41.53	Sepsis due to Serratia
Sepsis without shock	ICD-10	A41.59	Other Gram-negative sepsis
Sepsis without shock	ICD-10	A41.8	Other specified sepsis
Sepsis without shock	ICD-10	A41.81	Sepsis due to Enterococcus
Sepsis without shock	ICD-10	A41.89	Other specified sepsis
Sepsis without shock	ICD-10	A41.9	Sepsis, unspecified organism
Sepsis without shock	ICD-10	A42.7	Actinomycotic sepsis
Sepsis without shock	ICD-10	A54.86	Gonococcal sepsis
Sepsis without shock	ICD-10	B37.7	Candidal sepsis

Type of Non-Drug-Related Condition	Code Type	Code	Description
Sepsis without shock	ICD-10	O03.37	Sepsis following incomplete spontaneous abortion
Sepsis without shock	ICD-10	O03.87	Sepsis following complete or unspecified spontaneous abortion
Sepsis without shock	ICD-10	O04.87	Sepsis following (induced) termination of pregnancy
Sepsis without shock	ICD-10	O07.37	Sepsis following failed attempted termination of pregnancy
Sepsis without shock	ICD-10	O08.82	Sepsis following ectopic and molar pregnancy
Sepsis without shock	ICD-10	O85	Puerperal sepsis
Sepsis without shock	ICD-10	R65	Symptoms and signs specifically associated with systemic inflammation and infection
Sepsis without shock	ICD-10	R65.1	Systemic inflammatory response syndrome (SIRS) of non-infectious origin
Sepsis without shock	ICD-10	R65.10	Systemic inflammatory response syndrome (SIRS) of non-infectious origin without acute organ dysfunction
Sepsis without shock	ICD-10	R65.11	Systemic inflammatory response syndrome (SIRS) of non-infectious origin with acute organ dysfunction
Sepsis without shock	ICD-10	R65.20	Severe sepsis without septic shock
Sepsis without shock	ICD-10	R78.81	Bacteremia
Sepsis without shock	ICD-10	T81.44	Sepsis following a procedure
Sepsis without shock	ICD-10	T81.44XA	Sepsis following a procedure initial encounter
Sepsis without shock	ICD-10	T81.44XD	Sepsis following a procedure subsequent encounter
Sepsis without shock	ICD-10	T81.44XS	Sepsis following a procedure sequela
Shock	ICD-9	785.5	Shock without mention of trauma
Shock	ICD-9	785.50	Shock, unspecified
Shock	ICD-9	785.51	Cardiogenic shock
Shock	ICD-9	785.52	Septic shock
Shock	ICD-9	785.59	Other shock without mention of trauma
Shock	ICD-9	998.0	Postoperative shock not elsewhere classified
Shock	ICD-9	998.00	Postoperative shock, unspecified
Shock	ICD-9	998.01	Postoperative shock, cardiogenic
Shock	ICD-9	998.02	Postoperative shock, septic
Shock	ICD-9	998.09	Postoperative shock, other
Shock	ICD-10	R57	Shock, not elsewhere classified
Shock	ICD-10	R57.0	Cardiogenic shock
Shock	ICD-10	R57.1	Hypovolemic shock
Shock	ICD-10	R57.8	Other shock
Shock	ICD-10	R57.9	Shock, unspecified
Shock	ICD-10	R65.21	Severe sepsis with septic shock
Shock	ICD-10	T81.1	Postprocedural shock
Shock	ICD-10	T81.10	Postprocedural shock unspecified

Type of Non-Drug-Related Condition	Code Type	Code	Description
Shock	ICD-10	T81.10XA	Postprocedural shock, unspecified, initial encounter
Shock	ICD-10	T81.10XD	Postprocedural shock, unspecified, subsequent encounter
Shock	ICD-10	T81.10XS	Postprocedural shock, unspecified, sequela
Shock	ICD-10	T81.11	Postprocedural cardiogenic shock
Shock	ICD-10	T81.11XA	Postprocedural cardiogenic shock, unspecified, initial encounter
Shock	ICD-10	T81.11XD	Postprocedural cardiogenic shock, unspecified, subsequent encounter
Shock	ICD-10	T81.11XS	Postprocedural cardiogenic shock, unspecified, sequela
Shock	ICD-10	T81.12	Postprocedural septic shock
Shock	ICD-10	T81.12XA	Postprocedural septic shock, unspecified, initial encounter
Shock	ICD-10	T81.12XD	Postprocedural septic shock, unspecified, subsequent encounter
Shock	ICD-10	T81.12XS	Postprocedural septic shock, unspecified, sequela
Shock	ICD-10	T81.19	Other postprocedural shock
Shock	ICD-10	T81.19XA	Other postprocedural shock, unspecified, initial encounter
Shock	ICD-10	T81.19XD	Other postprocedural shock, unspecified, subsequent encounter
Shock	ICD-10	T81.19XS	Other postprocedural shock, unspecified, sequela
Sickle cell	ICD-9	282.5	Sickle cell trait
Sickle cell	ICD-9	282.6	Sickle cell anemia, unspecified
Sickle cell	ICD-9	282.61	Sickle cell anemia, without crisis
Sickle cell	ICD-9	282.62	Sickle cell anemia, with crisis
Sickle cell	ICD-9	282.63	Sickle cell/Hb-C disease without crisis
Sickle cell	ICD-9	282.64	Sickle cell/Hb-C disease with crisis
Sickle cell	ICD-9	282.68	Other sickle cell disease without crisis
Sickle cell	ICD-9	282.69	Other sickle cell disease with crisis
Sickle cell	ICD-10	D57.0	Sickle-cell anemia with crisis
Sickle cell	ICD-10	D57.00	Hb-SS disease with crisis, unspecified
Sickle cell	ICD-10	D57.01	Hb-SS disease with acute chest syndrome
Sickle cell	ICD-10	D57.02	Hb-SS disease with splenic sequestration
Sickle cell	ICD-10	D57.03	Hb-SS disease with cerebral vascular involvement
Sickle cell	ICD-10	D57.09	Hb-SS disease with crisis with other specified complication
Sickle cell	ICD-10	D57.1	Sickle-cell anemia without crisis
Sickle cell	ICD-10	D57.2	Double heterozygous sickling disorders
Sickle cell	ICD-10	D57.20	Sickle-cell/Hb-C disease without crisis
Sickle cell	ICD-10	D57.21	Sickle-cell/Hb-C disease with crisis
Sickle cell	ICD-10	D57.211	Sickle-cell/Hb-C disease with acute chest syndrome
Sickle cell	ICD-10	D57.212	Sickle-cell/Hb-C disease with splenic sequestration
Sickle cell	ICD-10	D57.213	Sickle-cell/Hb-C disease with cerebral vascular involvement

Type of Non-Drug-Related Condition	Code Type	Code	Description
Sickle cell	ICD-10	D57.218	Sickle-cell/Hb-C disease with crisis with other specified complication
Sickle cell	ICD-10	D57.219	Sickle-cell/Hb-C disease with crisis, unspecified
Sickle cell	ICD-10	D57.3	Sickle-cell trait
Sickle cell	ICD-10	D57.8	Other sickle-cell disorders
Sickle cell	ICD-10	D57.80	Other sickle-cell disorders without crisis
Sickle cell	ICD-10	D57.81	Other sickle-cell disorders with crisis
Sickle cell	ICD-10	D57.811	Other sickle-cell disorders with acute chest syndrome
Sickle cell	ICD-10	D57.812	Other sickle-cell disorders with splenic sequestration
Sickle cell	ICD-10	D57.813	Other sickle-cell disorders with cerebral vascular involvement
Sickle cell	ICD-10	D57.818	Other sickle-cell disorders with crisis with other specified complication
Sickle cell	ICD-10	D57.819	Other sickle-cell disorders with crisis, unspecified
Thalassemia	ICD-9	282.4	Thalassemias
Thalassemia	ICD-9	282.40	Thalassemia, unspecified
Thalassemia	ICD-9	282.41	Sickle-cell thalassemia without crisis
Thalassemia	ICD-9	282.42	Sickle-cell thalassemia with crisis
Thalassemia	ICD-9	282.43	Alpha thalassemia
Thalassemia	ICD-9	282.44	Beta thalassemia
Thalassemia	ICD-9	282.45	Delta-beta thalassemia
Thalassemia	ICD-9	282.46	Thalassemia minor
Thalassemia	ICD-9	282.47	Hemoglobin E-beta thalassemia
Thalassemia	ICD-9	282.49	Other thalassemia
Thalassemia	ICD-10	D56	Thalassemia
Thalassemia	ICD-10	D56.0	Alpha thalassemia
Thalassemia	ICD-10	D56.1	Beta thalassemia
Thalassemia	ICD-10	D56.2	Delta-beta thalassemia
Thalassemia	ICD-10	D56.3	Thalassemia minor
Thalassemia	ICD-10	D56.4	Hereditary persistence of fetal hemoglobin [HPFH]
Thalassemia	ICD-10	D56.5	Hemoglobin E-beta thalassemia
Thalassemia	ICD-10	D56.8	Other thalassemias
Thalassemia	ICD-10	D56.9	Thalassemia, unspecified
Thyrotoxicosis	ICD-9	242	Thyrotoxicosis with or without goiter
Thyrotoxicosis	ICD-9	242.0	Toxic diffuse goiter
Thyrotoxicosis	ICD-9	242.00	Toxic diffuse goiter without mention of thyrotoxic crisis or storm
Thyrotoxicosis	ICD-9	242.01	Toxic diffuse goiter with mention of thyrotoxic crisis or storm
Thyrotoxicosis	ICD-9	242.1	Toxic uninodular goiter
Thyrotoxicosis	ICD-9	242.10	Toxic uninodular goiter without mention of thyrotoxic crisis or storm

Type of Non-Drug-Related Condition	Code Type	Code	Description
Thyrotoxicosis	ICD-9	242.11	Toxic uninodular goiter with mention of thyrotoxic crisis or storm
Thyrotoxicosis	ICD-9	242.2	Toxic multinodular goiter
Thyrotoxicosis	ICD-9	242.20	Toxic multinodular goiter without mention of thyrotoxic crisis or storm
Thyrotoxicosis	ICD-9	242.21	Toxic multinodular goiter with mention of thyrotoxic crisis or storm
Thyrotoxicosis	ICD-9	242.3	Toxic nodular goiter, unspecified
Thyrotoxicosis	ICD-9	242.30	Toxic nodular goiter, unspecified type, without mention of thyrotoxic crisis or storm
Thyrotoxicosis	ICD-9	242.31	Toxic nodular goiter, unspecified type, with mention of thyrotoxic crisis or storm
Thyrotoxicosis	ICD-9	242.4	Thyrotoxicosis from ectopic thyroid nodule
Thyrotoxicosis	ICD-9	242.40	Thyrotoxicosis from ectopic thyroid nodule without mention of thyrotoxic crisis or storm
Thyrotoxicosis	ICD-9	242.41	Thyrotoxicosis from ectopic thyroid nodule with mention of thyrotoxic crisis or storm
Thyrotoxicosis	ICD-9	242.8	Thyrotoxicosis from other specified origin
Thyrotoxicosis	ICD-9	242.80	Thyrotoxicosis of other specified origin without mention of thyrotoxic crisis or storm
Thyrotoxicosis	ICD-9	242.81	Thyrotoxicosis of other specified origin with mention of thyrotoxic crisis or storm
Thyrotoxicosis	ICD-9	242.9	Thyrotoxicosis without mention of goiter or other cause, and without mention of thyrotoxic crisis or storm
Thyrotoxicosis	ICD-9	242.90	Thyrotoxicosis without mention of goiter or other cause, and without mention of thyrotoxic crisis or storm
Thyrotoxicosis	ICD-9	242.91	Thyrotoxicosis without mention of goiter or other cause, with mention of thyrotoxic crisis or storm
Thyrotoxicosis	ICD-10	E05	Thyrotoxicosis [hyperthyroidism]
Thyrotoxicosis	ICD-10	E05.0	Thyrotoxicosis with diffuse goiter
Thyrotoxicosis	ICD-10	E05.00	Thyrotoxicosis with diffuse goiter without thyrotoxic crisis or storm
Thyrotoxicosis	ICD-10	E05.01	Thyrotoxicosis with diffuse goiter with thyrotoxic crisis or storm
Thyrotoxicosis	ICD-10	E05.1	Thyrotoxicosis with toxic single thyroid nodule
Thyrotoxicosis	ICD-10	E05.10	Thyrotoxicosis with toxic single thyroid nodule without thyrotoxic crisis or storm
Thyrotoxicosis	ICD-10	E05.11	Thyrotoxicosis with toxic single thyroid nodule with thyrotoxic crisis or storm
Thyrotoxicosis	ICD-10	E05.2	Thyrotoxicosis with toxic multinodular goiter
Thyrotoxicosis	ICD-10	E05.20	Thyrotoxicosis with toxic multinodular goiter without thyrotoxic crisis or storm
Thyrotoxicosis	ICD-10	E05.21	Thyrotoxicosis with toxic multinodular goiter with thyrotoxic crisis or storm
Thyrotoxicosis	ICD-10	E05.3	Thyrotoxicosis from ectopic thyroid tissue
Thyrotoxicosis	ICD-10	E05.30	Thyrotoxicosis from ectopic thyroid tissue without thyrotoxic crisis or storm
Thyrotoxicosis	ICD-10	E05.31	Thyrotoxicosis from ectopic thyroid tissue with thyrotoxic crisis or storm
Thyrotoxicosis	ICD-10	E05.4	Thyrotoxicosis factitia
Thyrotoxicosis	ICD-10	E05.40	Thyrotoxicosis factitia without thyrotoxic crisis or storm
Thyrotoxicosis	ICD-10	E05.41	Thyrotoxicosis factitia with thyrotoxic crisis or storm
Thyrotoxicosis	ICD-10	E05.5	Thyroid crisis or storm

Type of Non-Drug-Related Condition	Code Type	Code	Description
Thyrotoxicosis	ICD-10	E05.8	Other thyrotoxicosis
Thyrotoxicosis	ICD-10	E05.80	Other thyrotoxicosis without thyrotoxic crisis or storm
Thyrotoxicosis	ICD-10	E05.81	Other thyrotoxicosis with thyrotoxic crisis or storm
Thyrotoxicosis	ICD-10	E05.9	Thyrotoxicosis, unspecified
Thyrotoxicosis	ICD-10	E05.90	Thyrotoxicosis, unspecified without thyrotoxic crisis or storm
Thyrotoxicosis	ICD-10	E05.91	Thyrotoxicosis, unspecified with thyrotoxic crisis or storm

Abbreviations: ICD-9=International Classification of Diseases, Ninth Revision; ICD-10=International Classification of Diseases, Tenth Revision

eTable 4. List of International Classification of Diseases, Ninth Revision (ICD-9) and International Classification of Diseases, Tenth Revision (ICD-10) diagnostic codes used to identify diabetes mellitus and hyperlipidemia.

Condition	Code Type	Code	Description
Diabetes mellitus	ICD-9	250.0x	Diabetes mellitus without mention of complication
Diabetes mellitus	ICD-9	250.1x	Diabetes with ketoacidosis
Diabetes mellitus	ICD-9	250.2x	Diabetes with hyperosmolarity
Diabetes mellitus	ICD-9	250.3x	Diabetes with other coma
Diabetes mellitus	ICD-9	250.4x	Diabetes with renal manifestations
Diabetes mellitus	ICD-9	250.5x	Diabetes with ophthalmic manifestations
Diabetes mellitus	ICD-9	250.6x	Diabetes with neurological manifestations
Diabetes mellitus	ICD-9	250.7x	Diabetes with peripheral circulatory disorders
Diabetes mellitus	ICD-9	250.8x	Diabetes with other specified manifestations
Diabetes mellitus	ICD-9	250.9x	Diabetes with unspecified complication
Diabetes mellitus	ICD-10	E10.1x	Type 1 diabetes mellitus with ketoacidosis
Diabetes mellitus	ICD-10	E10.21	Type 1 diabetes mellitus with diabetic nephropathy
Diabetes mellitus	ICD-10	E10.22	Type 1 diabetes mellitus with diabetic chronic kidney disease
Diabetes mellitus	ICD-10	E10.29	Type 1 diabetes mellitus with other diabetic kidney complication
Diabetes mellitus	ICD-10	E10.3x	Type 1 diabetes mellitus with diabetic retinopathy
Diabetes mellitus	ICD-10	E10.4x	Type 1 diabetes mellitus with diabetic neuropathy
Diabetes mellitus	ICD-10	E10.5x	Type 1 diabetes mellitus with diabetic peripheral angiopathy
Diabetes mellitus	ICD-10	E10.61x	Type 1 diabetes mellitus with diabetic arthropathy
Diabetes mellitus	ICD-10	E10.62x	Type 1 diabetes mellitus with skin complications
Diabetes mellitus	ICD-10	E10.63x	Type 1 diabetes mellitus with oral complications
Diabetes mellitus	ICD-10	E10.64x	Type 1 diabetes mellitus with hypoglycemia
Diabetes mellitus	ICD-10	E10.65	Type 1 diabetes mellitus with hyperglycemia
Diabetes mellitus	ICD-10	E10.69	Type 1 diabetes mellitus with other specified complication
Diabetes mellitus	ICD-10	E10.8	Type 1 diabetes mellitus with unspecified complications
Diabetes mellitus	ICD-10	E10.9	Type 1 diabetes mellitus without complications
Diabetes mellitus	ICD-10	E11.0x	Type 2 diabetes mellitus with hyperosmolarity
Diabetes mellitus	ICD-10	E11.1x	Type 2 diabetes mellitus with ketoacidosis
Diabetes mellitus	ICD-10	E11.21	Type 2 diabetes mellitus with diabetic nephropathy
Diabetes mellitus	ICD-10	E11.22	Type 2 diabetes mellitus with diabetic chronic kidney disease
Diabetes mellitus	ICD-10	E11.29	Type 2 diabetes mellitus with other diabetic kidney complication
Diabetes mellitus	ICD-10	E11.3x	Type 2 diabetes mellitus with diabetic retinopathy
Diabetes mellitus	ICD-10	E11.4x	Type 2 diabetes mellitus with diabetic neuropathy
Diabetes mellitus	ICD-10	E11.5x	Type 2 diabetes mellitus with diabetic peripheral angiopathy

Condition	Code Type	Code	Description
Diabetes mellitus	ICD-10	E11.61x	Type 2 diabetes mellitus with diabetic arthropathy
Diabetes mellitus	ICD-10	E11.62x	Type 2 diabetes mellitus with skin complications
Diabetes mellitus	ICD-10	E11.63x	Type 2 diabetes mellitus with oral complications
Diabetes mellitus	ICD-10	E11.64x	Type 2 diabetes mellitus with hypoglycemia
Diabetes mellitus	ICD-10	E11.65	Type 2 diabetes mellitus with hyperglycemia
Diabetes mellitus	ICD-10	E11.69	Type 2 diabetes mellitus with other specified complication
Diabetes mellitus	ICD-10	E11.8	Type 2 diabetes mellitus with unspecified complications
Diabetes mellitus	ICD-10	E11.9	Type 2 diabetes mellitus without complications
Diabetes mellitus	ICD-10	E13.0x	Other specified diabetes mellitus with hyperosmolarity
Diabetes mellitus	ICD-10	E13.1x	Other specified diabetes mellitus with ketoacidosis
Diabetes mellitus	ICD-10	E13.21	Other specified diabetes mellitus with diabetic nephropathy
Diabetes mellitus	ICD-10	E13.22	Other specified diabetes mellitus with diabetic chronic kidney disease
Diabetes mellitus	ICD-10	E13.29	Other specified diabetes mellitus with other diabetic kidney complication
Diabetes mellitus	ICD-10	E13.3x	Other specified diabetes mellitus with diabetic retinopathy
Diabetes mellitus	ICD-10	E13.4x	Other specified diabetes mellitus with diabetic neuropathy
Diabetes mellitus	ICD-10	E13.5x	Other specified diabetes mellitus with diabetic peripheral angiopathy
Diabetes mellitus	ICD-10	E13.61x	Other specified diabetes mellitus with diabetic arthropathy
Diabetes mellitus	ICD-10	E13.62x	Other specified diabetes mellitus with skin complications
Diabetes mellitus	ICD-10	E13.63x	Other specified diabetes mellitus with oral complications
Diabetes mellitus	ICD-10	E13.64x	Other specified diabetes mellitus with hypoglycemia
Diabetes mellitus	ICD-10	E13.65	Other specified diabetes mellitus with hyperglycemia
Diabetes mellitus	ICD-10	E13.69	Other specified diabetes mellitus with other specified complication
Diabetes mellitus	ICD-10	E13.8	Other specified diabetes mellitus with unspecified complications
Diabetes mellitus	ICD-10	E13.9	Other specified diabetes mellitus without complications
Hyperlipidemia	ICD-9	272.0	Pure hypercholesterolemia
Hyperlipidemia	ICD-9	272.1	Hypertriglyceridemia
Hyperlipidemia	ICD-9	272.2	Mixed hyperlipidemia
Hyperlipidemia	ICD-9	272.3	Hyperchylomicronemia
Hyperlipidemia	ICD-9	272.4	Other and unspecified hyperlipidemia
Hyperlipidemia	ICD-9	272.5	Hyperlipidemia, unspecified
Hyperlipidemia	ICD-10	E78.00	Pure hypercholesterolemia, unspecified
Hyperlipidemia	ICD-10	E78.01	Familial hypercholesterolemia
Hyperlipidemia	ICD-10	E78.1	Pure hyperglyceridemia
Hyperlipidemia	ICD-10	E78.2	Mixed hyperlipidemia
Hyperlipidemia	ICD-10	E78.3	Hyperchylomicronemia
Hyperlipidemia	ICD-10	E78.49	Other hyperlipidemia

Condition	Code Type	Code	Description
Hyperlipidemia	ICD-10	E78.5	Hyperlipidemia, unspecified
Hyperlipidemia	ICD-10	E78.6	Lipoprotein deficiency
Hyperlipidemia	ICD-10	E78.9	Disorder of lipoprotein metabolism, unspecified

Abbreviations: ICD-9=International Classification of Diseases, Ninth Revision; ICD-10=International Classification of Diseases, Tenth Revision

eTable 5. List of International Classification of Diseases, Ninth Revision (ICD-9) and International Classification of Diseases, Tenth Revision (ICD-10) diagnostic codes and Current Procedural Terminology (CPT) procedure codes used to identify liver transplantation.

Code Type	Code	Description
ICD-9	996.82	Complications of transplanted liver
ICD-9	50.5x	Liver transplant
ICD-9	V42.7	Liver replaced by transplant
ICD-10	T86.40	Unspecified complication of liver transplant
ICD-10	T86.41	Liver transplant rejection
ICD-10	T86.42	Liver transplant failure
ICD-10	T86.43	Liver transplant infection
ICD-10	T86.49	Other complications of liver transplant
ICD-10	Z48.23	Encounter for aftercare following liver transplant
ICD-10	Z94.4	Liver transplant status
CPT	47133	Donor hepatectomy, from cadaver donor
CPT	47135	Liver allotransplantation; orthotopic, partial or whole
CPT	47136	Liver allotransplantation; heterotopic, partial or whole, from cadaver or living donor, any age
CPT	47143	Preparation of cadaver donor whole liver graft prior to allotransplantation; without trisegment or lobe split
CPT	47144	Preparation of cadaver donor whole liver graft prior to allotransplantation; with trisegment split of whole liver graft into two partial liver grafts
CPT	47145	Preparation of cadaver donor whole liver graft prior to allotransplantation; with lobe split into two partial liver grafts
CPT	47146	Backbench reconstruction of cadaver or living donor liver graft prior to allotransplantation; venous anastomosis
CPT	47147	Backbench reconstruction of cadaver or living donor liver graft prior to allotransplantation; arterial anastomosis

Abbreviations: CPT=Current Procedural Terminology; ICD-9=International Classification of Diseases, Ninth Revision; ICD-10=International Classification of Diseases, Tenth Revision

eTable 6. Reasons for exclusion of patients within each of the 194 medication initiator cohorts.

Medication	Original Category[a]	No. Patients	<1 Year of Baseline	Baseline Anti-Coagulant Use	Baseline Severe Acute Liver Injury[b]	Pre-Existing Liver/Biliary Disease[c]	Other Baseline Evidence of Liver Injury[d]	Final Sample
ALLOPURINOL	A	612,366	152,146 (24.8%)	49,693 (8.1%)	918 (0.1%)	62,759 (10.2%)	21,906 (3.6%)	324,944
AMIODARONE	A	267,031	48,421 (18.1%)	93,500 (35.0%)	671 (0.3%)	22,330 (8.4%)	5,301 (2.0%)	96,808
AMOXICILLIN WITH CLAVULANATE	A	1,998,069	256,264 (12.8%)	130,020 (6.5%)	7,427 (0.4%)	299,773 (15.0%)	69,442 (3.5%)	1,235,143
ANDROGENIC STEROIDS[e]	A	195,051	27,936 (14.3%)	7,219 (3.7%)	186 (0.1%)	20,192 (10.4%)	11,581 (5.9%)	127,937
ATORVASTATIN	A	2,328,287	291,845 (12.5%)	143,771 (6.2%)	1,875 (0.1%)	258,228 (11.1%)	88,797 (3.8%)	1,543,771
AURANOFIN OR GOLD PRODUCTS[e]	A	299	79 (26.4%)	13 (4.3%)	0 (0.0%)	42 (14.0%)	16 (5.4%)	149
AZATHIOPRINE OR MERCAPTOPURINE	A	31,240	7,067 (22.6%)	1,827 (5.8%)	241 (0.8%)	4,433 (14.2%)	1,639 (5.2%)	16,033
BUSULFAN	A	144	26 (18.1%)	21 (14.6%)	0 (0.0%)	28 (19.4%)	9 (6.3%)	60
CARBAMAZEPINE	A	120,184	22,278 (18.5%)	3,695 (3.1%)	196 (0.2%)	21,106 (17.6%)	4,409 (3.7%)	68,500
CHLORPROMAZINE	A	36,637	4,547 (12.4%)	1,991 (5.4%)	390 (1.1%)	10,691 (29.2%)	1,569 (4.3%)	17,449
DANTROLENE	A	3,361	687 (20.4%)	285 (8.5%)	3 (0.1%)	512 (15.2%)	130 (3.9%)	1,744
DICLOFENAC	A	865,043	155,285 (18.0%)	10,671 (1.2%)	621 (0.1%)	102,037 (11.8%)	29,028 (3.4%)	567,401
DIDANOSINE	A	2,594	704 (27.1%)	35 (1.3%)	14 (0.5%)	755 (29.1%)	196 (7.6%)	890
EFAVIRENZ	A	13,762	3,948 (28.7%)	213 (1.5%)	53 (0.4%)	3,870 (28.1%)	718 (5.2%)	4,960
ERYTHROMYCIN	A	128,569	22,192 (17.3%)	5,246 (4.1%)	389 (0.3%)	19,101 (14.9%)	4,543 (3.5%)	77,098
ESTROGENS OR PROGESTINS[e]	A	354,603	88,487 (25.0%)	18,187 (5.1%)	2,038 (0.6%)	55,282 (15.6%)	10,928 (3.1%)	179,681
FLUTAMIDE	A	16,207	3,793 (23.4%)	1,022 (6.3%)	12 (0.1%)	1,155 (7.1%)	992 (6.1%)	9,233
HYDRALAZINE OR ISOSORBIDE DINITRATE	A	296,091	35,148 (11.9%)	36,839 (12.4%)	1,019 (0.3%)	54,641 (18.5%)	9,770 (3.3%)	158,674
IBUPROFEN	A	2,601,823	747,227 (28.7%)	35,300 (1.4%)	3,198 (0.1%)	271,319 (10.4%)	64,959 (2.5%)	1,479,820
INFLIXIMAB	A	1,837	233 (12.7%)	71 (3.9%)	4 (0.2%)	254 (13.8%)	161 (8.8%)	1,114
INTERFERON-ß	A	6,650	2,327 (35.0%)	80 (1.2%)	2 (0.0%)	333 (5.0%)	235 (3.5%)	3,673
ISONIAZID	A	45,772	9,214 (20.1%)	1,892 (4.1%)	116 (0.3%)	12,550 (27.4%)	1,524 (3.3%)	20,476
KETOCONAZOLE	A	47,909	8,808 (18.4%)	1,549 (3.2%)	57 (0.1%)	4,956 (10.3%)	2,563 (5.3%)	29,976
METHOTREXATE	A	91,560	19,520 (21.3%)	4,467 (4.9%)	51 (0.1%)	7,802 (8.5%)	3,605 (3.9%)	56,115
METHYLDOPA	A	5,009	1,570 (31.3%)	169 (3.4%)	2 (0.0%)	341 (6.8%)	84 (1.7%)	2,843
MINOCYCLINE	A	156,877	17,305 (11.0%)	11,427 (7.3%)	368 (0.2%)	24,735 (15.8%)	5,516 (3.5%)	97,526
NEVIRAPINE	A	2,829	849 (30.0%)	39 (1.4%)	5 (0.2%)	751 (26.5%)	182 (6.4%)	1,003
NITROFURANTOIN	A	359,882	33,456 (9.3%)	41,134 (11.4%)	881 (0.2%)	54,048 (15.0%)	10,412 (2.9%)	219,951
PHENYTOIN OR FOSPHENYTOIN	A	80,150	27,883 (34.8%)	4,646 (5.8%)	260 (0.3%)	12,704 (15.9%)	2,813 (3.5%)	31,844
PROPYLTHIOURACIL	A	4,821	1,378 (28.6%)	599 (12.4%)	8 (0.2%)	679 (14.1%)	215 (4.5%)	1,942
PYRAZINAMIDE	A	4,748	879 (18.5%)	235 (4.9%)	27 (0.6%)	1,409 (29.7%)	264 (5.6%)	1,934
QUINIDINE	A	7,075	3,026 (42.8%)	518 (7.3%)	14 (0.2%)	438 (6.2%)	153 (2.2%)	2,926
RIFAMPIN	A	51,221	5,930 (11.6%)	4,293 (8.4%)	277 (0.5%)	13,255 (25.9%)	2,139 (4.2%)	25,327
SIMVASTATIN	A	3,248,110	1,322,419 (40.7%)	92,170 (2.8%)	1,392 (0.0%)	186,302 (5.7%)	78,158 (2.4%)	1,567,669
SULFAMETHOXAZOLE WITH TRIMETHOPRIM	A	1,640,476	230,362 (14.0%)	82,178 (5.0%)	5,628 (0.3%)	242,264 (14.8%)	54,921 (3.3%)	1,025,123
SULFASALAZINE	A	46,041	9,684 (21.0%)	2,068 (4.5%)	52 (0.1%)	5,527 (12.0%)	2,375 (5.2%)	26,335
SULINDAC	A	238,832	42,148 (17.6%)	3,211 (1.3%)	214 (0.1%)	29,708 (12.4%)	8,032 (3.4%)	155,519
TELITHROMYCIN	A	32	2 (6.3%)	8 (25.0%)	0 (0.0%)	4 (12.5%)	1 (3.1%)	17
THIOGUANINE	A	123	35 (28.5%)	7 (5.7%)	2 (1.6%)	32 (26.0%)	9 (7.3%)	38

© 2024 Torgersen J et al. *JAMA Internal Medicine.*

Medication	Original Category[a]	No. Patients	<1 Year of Baseline	Baseline Anti-Coagulant Use	Baseline Severe Acute Liver Injury[b]	Pre-Existing Liver/Biliary Disease[c]	Other Baseline Evidence of Liver Injury[d]	Final Sample
TICLOPIDINE	A	4,077	1,466 (36.0%)	161 (3.9%)	5 (0.1%)	204 (5.0%)	104 (2.6%)	2,137
VALPROATE	A	439,141	82,807 (18.9%)	12,788 (2.9%)	437 (0.1%)	85,898 (19.6%)	13,164 (3.0%)	244,047
ACARBOSE	B	73,908	7,042 (9.5%)	4,676 (6.3%)	68 (0.1%)	6,859 (9.3%)	4,863 (6.6%)	50,400
AMITRIPTYLINE	B	642,731	142,248 (22.1%)	21,151 (3.3%)	838 (0.1%)	77,118 (12.0%)	21,689 (3.4%)	379,687
AMOXICILLIN	B	1,741,588	254,466 (14.6%)	98,107 (5.6%)	3,535 (0.2%)	238,460 (13.7%)	54,827 (3.1%)	1,092,193
ASPARAGINASE OR PEGASPARGASE	B	17	0 (0.0%)	2 (11.8%)	0 (0.0%)	4 (23.5%)	2 (11.8%)	9
AZITHROMYCIN	B	2,038,855	296,212 (14.5%)	100,902 (4.9%)	2,964 (0.1%)	265,326 (13.0%)	69,491 (3.4%)	1,303,960
CAPTOPRIL	B	55,099	25,686 (46.6%)	4,502 (8.2%)	181 (0.3%)	4,600 (8.3%)	1,267 (2.3%)	18,863
CEFTRIAXONE	B	36,092	6,005 (16.6%)	987 (2.7%)	63 (0.2%)	5,925 (16.4%)	831 (2.3%)	22,281
CELECOXIB	B	218,437	33,972 (15.6%)	13,464 (6.2%)	172 (0.1%)	23,207 (10.6%)	7,114 (3.3%)	140,508
CHLORPROPAMIDE	B	480	336 (70.0%)	9 (1.9%)	0 (0.0%)	16 (3.3%)	8 (1.7%)	111
CHLORZOXAZONE	B	36,256	6,656 (18.4%)	1,002 (2.8%)	21 (0.1%)	4,257 (11.7%)	992 (2.7%)	23,328
CIMETIDINE	B	43,074	14,385 (33.4%)	898 (2.1%)	65 (0.2%)	4,451 (10.3%)	1,160 (2.7%)	22,115
CIPROFLOXACIN	B	1,816,459	221,701 (12.2%)	110,508 (6.1%)	12,945 (0.7%)	282,180 (15.5%)	63,665 (3.5%)	1,125,460
CLARITHROMYCIN	B	341,383	47,787 (14.0%)	13,291 (3.9%)	1,026 (0.3%)	57,180 (16.7%)	11,743 (3.4%)	210,356
CLINDAMYCIN	B	787,990	71,376 (9.1%)	66,005 (8.4%)	2,377 (0.3%)	139,949 (17.8%)	27,388 (3.5%)	480,895
CLOPIDOGREL	B	1,066,528	229,886 (21.6%)	70,450 (6.6%)	1,176 (0.1%)	101,255 (9.5%)	27,528 (2.6%)	636,233
CLOXACILLIN	B	75	21 (28.0%)	5 (6.7%)	0 (0.0%)	11 (14.7%)	2 (2.7%)	36
CLOZAPINE	B	4,684	393 (8.4%)	120 (2.6%)	6 (0.1%)	1,512 (32.3%)	235 (5.0%)	2,418
CYCLOPHOSPHAMIDE OR IFOSPHAMIDE	B	6,954	1,085 (15.6%)	867 (12.5%)	25 (0.4%)	1,565 (22.5%)	428 (6.2%)	2,984
DICLOXACILLIN	B	135,905	18,326 (13.5%)	10,250 (7.5%)	504 (0.4%)	22,998 (16.9%)	5,056 (3.7%)	78,771
DOXORUBICIN	B	371	71 (19.1%)	25 (6.7%)	4 (1.1%)	108 (29.1%)	11 (3.0%)	152
DULOXETINE	B	558,051	49,749 (8.9%)	30,744 (5.5%)	598 (0.1%)	85,794 (15.4%)	19,302 (3.5%)	371,864
ENALAPRIL	B	189,514	59,276 (31.3%)	10,632 (5.6%)	226 (0.1%)	13,751 (7.3%)	4,458 (2.4%)	101,171
ETANERCEPT	B	30,487	4,334 (14.2%)	1,284 (4.2%)	29 (0.1%)	4,185 (13.7%)	2,432 (8.0%)	18,223
ETHIONAMIDE	B	28	5 (17.9%)	2 (7.1%)	0 (0.0%)	6 (21.4%)	3 (10.7%)	12
FELBAMATE	B	55	15 (27.3%)	1 (1.8%)	0 (0.0%)	5 (9.1%)	1 (1.8%)	33
FENOFIBRATE	B	130,075	14,331 (11.0%)	7,683 (5.9%)	85 (0.1%)	12,888 (9.9%)	10,151 (7.8%)	84,937
FLUCONAZOLE	B	475,801	70,384 (14.8%)	24,317 (5.1%)	2,321 (0.5%)	74,979 (15.8%)	16,154 (3.4%)	287,646
FLUVASTATIN	B	122,739	17,177 (14.0%)	6,718 (5.5%)	73 (0.1%)	8,185 (6.7%)	6,911 (5.6%)	83,675
GLYBURIDE (GLIBENCLAMIDE)	B	379,651	157,869 (41.6%)	15,058 (4.0%)	577 (0.2%)	23,316 (6.1%)	16,622 (4.4%)	166,209
HALOPERIDOL	B	92,452	15,132 (16.4%)	5,757 (6.2%)	931 (1.0%)	25,705 (27.8%)	3,171 (3.4%)	41,756
IMATINIB	B	6,848	1,462 (21.3%)	496 (7.2%)	23 (0.3%)	1,202 (17.6%)	299 (4.4%)	3,366
IMIPRAMINE	B	35,014	6,728 (19.2%)	1,383 (3.9%)	28 (0.1%)	4,365 (12.5%)	959 (2.7%)	21,551
IRINOTECAN	B	201	46 (22.9%)	23 (11.4%)	2 (1.0%)	76 (37.8%)	12 (6.0%)	42
ITRACONAZOLE	B	24,490	4,290 (17.5%)	887 (3.6%)	48 (0.2%)	2,954 (12.1%)	934 (3.8%)	15,377
LAMOTRIGINE	B	193,391	28,502 (14.7%)	5,223 (2.7%)	220 (0.1%)	34,705 (17.9%)	6,799 (3.5%)	117,942
LEFLUNOMIDE	B	20,191	2,533 (12.5%)	1,369 (6.8%)	9 (0.0%)	2,120 (10.5%)	1,201 (5.9%)	12,959
LEVOFLOXACIN OR OFLOXACIN	B	1,026,045	157,279 (15.3%)	77,460 (7.5%)	5,669 (0.6%)	167,378 (16.3%)	38,049 (3.7%)	580,210
LISINOPRIL	B	3,136,396	1,142,174 (36.4%)	111,659 (3.6%)	2,736 (0.1%)	228,030 (7.3%)	86,788 (2.8%)	1,565,009
LOVASTATIN	B	506,415	161,420 (31.9%)	20,149 (4.0%)	196 (0.0%)	26,655 (5.3%)	16,292 (3.2%)	281,703
MELPHALAN	B	3,818	611 (16.0%)	525 (13.8%)	10 (0.3%)	731 (19.1%)	174 (4.6%)	1,767

Medication	Original Category[a]	No. Patients	<1 Year of Baseline	Baseline Anti-Coagulant Use	Baseline Severe Acute Liver Injury[b]	Pre-Existing Liver/Biliary Disease[c]	Other Baseline Evidence of Liver Injury[d]	Final Sample
METFORMIN	B	1,505,207	450,240 (29.9%)	59,320 (3.9%)	1,234 (0.1%)	114,232 (7.6%)	81,263 (5.4%)	798,918
METHIMAZOLE (THIAMAZOLE)	B	29,508	5,273 (17.9%)	3,850 (13.0%)	43 (0.1%)	7,421 (25.1%)	1,056 (3.6%)	11,865
MOXIFLOXACIN	B	616,421	49,684 (8.1%)	47,399 (7.7%)	3,069 (0.5%)	115,145 (18.7%)	24,757 (4.0%)	376,367
NAPROXEN	B	2,316,826	577,820 (24.9%)	29,932 (1.3%)	1,875 (0.1%)	239,482 (10.3%)	63,126 (2.7%)	1,404,591
NIFEDIPINE	B	306,884	85,315 (27.8%)	13,920 (4.5%)	461 (0.2%)	36,760 (12.0%)	8,095 (2.6%)	162,333
OLANZAPINE	B	199,009	40,365 (20.3%)	7,716 (3.9%)	653 (0.3%)	46,631 (23.4%)	5,319 (2.7%)	98,325
OMEPRAZOLE OR ESOMEPRAZOLE	B	3,514,693	716,415 (20.4%)	167,100 (4.8%)	10,182 (0.3%)	406,436 (11.6%)	110,199 (3.1%)	2,104,361
OXACILLIN	B	30	6 (20.0%)	3 (10.0%)	0 (0.0%)	4 (13.3%)	1 (3.3%)	16
OXALIPLATIN	B	227	35 (15.4%)	20 (8.8%)	5 (2.2%)	87 (38.3%)	15 (6.6%)	65
PAROXETINE	B	462,996	127,546 (27.5%)	12,966 (2.8%)	671 (0.1%)	60,259 (13.0%)	12,298 (2.7%)	249,256
PENICILLAMINE	B	754	179 (23.7%)	21 (2.8%)	2 (0.3%)	125 (16.6%)	26 (3.4%)	401
PHENOBARBITAL	B	10,693	3,892 (36.4%)	354 (3.3%)	79 (0.7%)	2,119 (19.8%)	320 (3.0%)	3,929
PIROXICAM	B	218,154	50,727 (23.3%)	2,649 (1.2%)	142 (0.1%)	23,061 (10.6%)	6,636 (3.0%)	134,939
PROPAFENONE	B	14,909	4,342 (29.1%)	4,468 (30.0%)	7 (0.0%)	562 (3.8%)	180 (1.2%)	5,350
QUININE	B	83,432	19,499 (23.4%)	4,677 (5.6%)	228 (0.3%)	6,995 (8.4%)	2,795 (3.4%)	49,238
RANITIDINE	B	1,658,068	434,338 (26.2%)	66,015 (4.0%)	3,646 (0.2%)	189,216 (11.4%)	52,829 (3.2%)	912,024
ROSUVASTATIN	B	853,733	74,071 (8.7%)	58,391 (6.8%)	482 (0.1%)	79,442 (9.3%)	41,639 (4.9%)	599,708
SERTRALINE	B	1,472,779	356,480 (24.2%)	54,769 (3.7%)	2,232 (0.2%)	192,861 (13.1%)	38,218 (2.6%)	828,219
STAVUDINE	B	2,960	1,320 (44.6%)	37 (1.3%)	14 (0.5%)	702 (23.7%)	137 (4.6%)	750
TAMOXIFEN	B	9,454	2,069 (21.9%)	409 (4.3%)	17 (0.2%)	1,065 (11.3%)	355 (3.8%)	5,539
TERBINAFINE	B	404,848	54,941 (13.6%)	16,270 (4.0%)	197 (0.0%)	36,835 (9.1%)	13,418 (3.3%)	283,187
THIABENDAZOLE	B	485	79 (16.3%)	20 (4.1%)	2 (0.4%)	57 (11.8%)	15 (3.1%)	312
THIORIDAZINE	B	2,616	779 (29.8%)	55 (2.1%)	4 (0.2%)	522 (20.0%)	68 (2.6%)	1,188
VENLAFAXINE OR DEXVENLAFAXINE	B	630,606	117,725 (18.7%)	19,278 (3.1%)	900 (0.1%)	97,237 (15.4%)	19,623 (3.1%)	375,843
VORICONAZOLE	B	6,476	647 (10.0%)	729 (11.3%)	98 (1.5%)	2,006 (31.0%)	378 (5.8%)	2,618
ZIDOVUDINE	B	12,800	7,542 (58.9%)	92 (0.7%)	37 (0.3%)	1,961 (15.3%)	385 (3.0%)	2,783
ABACAVIR	C	11,239	2,162 (19.2%)	296 (2.6%)	59 (0.5%)	3,670 (32.7%)	705 (6.3%)	4,347
ACEBUTOLOL	C	869	367 (42.2%)	70 (8.1%)	0 (0.0%)	48 (5.5%)	14 (1.6%)	370
ACITRETIN	C	14,722	1,678 (11.4%)	801 (5.4%)	14 (0.1%)	1,905 (12.9%)	681 (4.6%)	9,643
ADALIMUMAB	C	47,907	5,331 (11.1%)	1,942 (4.1%)	42 (0.1%)	6,667 (13.9%)	3,585 (7.5%)	30,340
ALBENDAZOLE	C	4,990	441 (8.8%)	207 (4.1%)	7 (0.1%)	730 (14.6%)	162 (3.2%)	3,443
ALFUZOSIN	C	137,641	7,463 (5.4%)	11,041 (8.0%)	198 (0.1%)	18,716 (13.6%)	3,707 (2.7%)	96,516
AMLODIPINE	C	1,870,558	325,133 (17.4%)	85,559 (4.6%)	2,589 (0.1%)	214,472 (11.5%)	56,628 (3.0%)	1,186,177
AMPHETAMINE[f]	C	111,061	28,032 (25.2%)	999 (0.9%)	45 (0.0%)	9,673 (8.7%)	2,931 (2.6%)	69,381
ANAKINRA	C	939	60 (6.4%)	151 (16.1%)	1 (0.1%)	220 (23.4%)	83 (8.8%)	424
ATOMOXETINE	C	30,506	4,307 (14.1%)	360 (1.2%)	25 (0.1%)	6,269 (20.6%)	856 (2.8%)	18,689
BOSENTAN	C	475	44 (9.3%)	162 (34.1%)	1 (0.2%)	52 (10.9%)	17 (3.6%)	199
BUPROPION	C	1,396,975	315,431 (22.6%)	33,882 (2.4%)	1,254 (0.1%)	189,846 (13.6%)	37,971 (2.7%)	818,591
CANDESARTAN	C	37,949	14,182 (37.4%)	2,549 (6.7%)	24 (0.1%)	1,758 (4.6%)	1,170 (3.1%)	18,266
CEPHALEXIN	C	1,849,301	233,375 (12.6%)	140,475 (7.6%)	4,467 (0.2%)	261,521 (14.1%)	62,085 (3.4%)	1,147,378
CITALOPRAM OR ESCITALOPRAM	C	1,521,239	381,250 (25.1%)	56,085 (3.7%)	2,616 (0.2%)	197,205 (13.0%)	39,607 (2.6%)	844,476
CLOMIPHENE	C	9,399	967 (10.3%)	161 (1.7%)	5 (0.1%)	847 (9.0%)	436 (4.6%)	6,983

Medication	Original Category[a]	No. Patients	<1 Year of Baseline	Baseline Anti-Coagulant Use	Baseline Severe Acute Liver Injury[b]	Pre-Existing Liver/Biliary Disease[c]	Other Baseline Evidence of Liver Injury[d]	Final Sample
CYPROHEPTADINE	C	113,968	15,139 (13.3%)	4,963 (4.4%)	392 (0.3%)	24,235 (21.3%)	4,000 (3.5%)	65,239
CYTARABINE	C	90	15 (16.7%)	7 (7.8%)	1 (1.1%)	28 (31.1%)	10 (11.1%)	29
DIFLUNISAL	C	7,148	1,462 (20.5%)	110 (1.5%)	10 (0.1%)	1,091 (15.3%)	238 (3.3%)	4,237
DILTIAZEM	C	464,582	140,534 (30.2%)	73,377 (15.8%)	754 (0.2%)	39,653 (8.5%)	10,470 (2.3%)	199,794
DISOPYRAMIDE	C	1,290	638 (49.5%)	148 (11.5%)	0 (0.0%)	54 (4.2%)	18 (1.4%)	432
DOXYCYCLINE	C	1,600,292	169,947 (10.6%)	111,376 (7.0%)	3,075 (0.2%)	238,256 (14.9%)	53,738 (3.4%)	1,023,900
ERLOTINIB	C	10,846	1,276 (11.8%)	1,118 (10.3%)	169 (1.6%)	3,265 (30.1%)	662 (6.1%)	4,356
ETODOLAC	C	969,852	213,911 (22.1%)	14,468 (1.5%)	825 (0.1%)	111,712 (11.5%)	29,788 (3.1%)	599,148
ETOPOSIDE	C	8,631	1,041 (12.1%)	920 (10.7%)	75 (0.9%)	2,724 (31.6%)	518 (6.0%)	3,353
EZETIMIBE	C	235,425	17,380 (7.4%)	19,307 (8.2%)	155 (0.1%)	20,429 (8.7%)	13,552 (5.8%)	164,602
FAMOTIDINE	C	419,185	25,977 (6.2%)	34,578 (8.2%)	934 (0.2%)	68,191 (16.3%)	13,156 (3.1%)	276,349
FLAVOCOXID	C	7	0 (0.0%)	0 (0.0%)	0 (0.0%)	0 (0.0%)	0 (0.0%)	7
FLUOROURACIL	C	3,391	440 (13.0%)	269 (7.9%)	57 (1.7%)	981 (28.9%)	128 (3.8%)	1,516
FLUOXETINE	C	739,640	176,154 (23.8%)	19,878 (2.7%)	979 (0.1%)	101,688 (13.7%)	20,344 (2.8%)	420,597
GABAPENTIN	C	2,252,034	342,175 (15.2%)	115,491 (5.1%)	4,056 (0.2%)	319,421 (14.2%)	76,793 (3.4%)	1,394,098
GEFITINIB	C	1,557	337 (21.6%)	168 (10.8%)	6 (0.4%)	323 (20.7%)	122 (7.8%)	601
GEMCITABINE	C	494	50 (10.1%)	55 (11.1%)	24 (4.9%)	121 (24.5%)	26 (5.3%)	218
GEMFIBROZIL	C	587,462	157,106 (26.7%)	22,746 (3.9%)	307 (0.1%)	43,693 (7.4%)	34,543 (5.9%)	329,067
GLIMEPIRIDE	C	62,092	9,203 (14.8%)	4,783 (7.7%)	36 (0.1%)	6,366 (10.3%)	3,387 (5.5%)	38,317
GLIPIZIDE	C	898,704	214,707 (23.9%)	49,644 (5.5%)	1,307 (0.1%)	80,959 (9.0%)	51,564 (5.7%)	500,523
HYDROXYUREA	C	15,271	3,239 (21.2%)	1,331 (8.7%)	37 (0.2%)	2,215 (14.5%)	623 (4.1%)	7,826
INDOMETHACIN	C	473,544	80,568 (17.0%)	14,685 (3.1%)	558 (0.1%)	57,891 (12.2%)	17,383 (3.7%)	302,459
IRBESARTAN	C	160,548	60,609 (37.8%)	8,741 (5.4%)	75 (0.0%)	7,484 (4.7%)	4,300 (2.7%)	79,339
ISOTRETINOIN	C	6,957	1,325 (19.0%)	152 (2.2%)	1 (0.0%)	649 (9.3%)	263 (3.8%)	4,567
KETOPROFEN	C	3,071	575 (18.7%)	39 (1.3%)	5 (0.2%)	482 (15.7%)	103 (3.4%)	1,867
LABETALOL	C	39,597	8,930 (22.6%)	2,229 (5.6%)	126 (0.3%)	7,951 (20.1%)	1,103 (2.8%)	19,258
LANSOPRAZOLE OR DEXLANSOPRAZOLE	C	300,473	113,878 (37.9%)	13,738 (4.6%)	942 (0.3%)	29,051 (9.7%)	8,372 (2.8%)	134,492
LENALIDOMIDE OR THALIDOMIDE	C	18,282	2,568 (14.0%)	2,448 (13.4%)	65 (0.4%)	4,090 (22.4%)	920 (5.0%)	8,191
LEVETIRACETAM	C	122,058	15,834 (13.0%)	12,152 (10.0%)	677 (0.6%)	25,650 (21.0%)	4,477 (3.7%)	63,268
LEVOCETIRIZINE OR CETIRIZINE	C	1,192,714	145,266 (12.2%)	54,860 (4.6%)	1,265 (0.1%)	141,075 (11.8%)	41,787 (3.5%)	808,461
LINEZOLID	C	43,911	3,078 (7.0%)	7,806 (17.8%)	734 (1.7%)	14,578 (33.2%)	1,912 (4.4%)	15,803
LOSARTAN	C	911,311	147,112 (16.1%)	58,968 (6.5%)	683 (0.1%)	88,238 (9.7%)	31,734 (3.5%)	584,576
MELOXICAM	C	1,460,153	201,441 (13.8%)	26,555 (1.8%)	1,074 (0.1%)	174,849 (12.0%)	49,583 (3.4%)	1,006,651
MESALAMINE[g]	C	51,216	14,544 (28.4%)	2,073 (4.0%)	59 (0.1%)	5,146 (10.0%)	1,453 (2.8%)	27,941
METHOXSALEN	C	1,655	316 (19.1%)	55 (3.3%)	2 (0.1%)	215 (13.0%)	81 (4.9%)	986
METHYLPHENIDATE OR DEXMETHYLPHENIDATE	C	133,323	27,643 (20.7%)	3,298 (2.5%)	234 (0.2%)	16,056 (12.0%)	4,506 (3.4%)	81,586
METRONIDAZOLE	C	744,282	97,625 (13.1%)	43,180 (5.8%)	8,057 (1.1%)	144,301 (19.4%)	27,453 (3.7%)	423,666
MEXILETINE	C	8,149	1,272 (15.6%)	2,265 (27.8%)	31 (0.4%)	894 (11.0%)	264 (3.2%)	3,423
MIRTAZAPINE	C	923,157	144,052 (15.6%)	45,156 (4.9%)	2,856 (0.3%)	193,171 (20.9%)	25,519 (2.8%)	512,403
MITOMYCIN	C	3,382	149 (4.4%)	307 (9.1%)	9 (0.3%)	519 (15.3%)	88 (2.6%)	2,310
MONTELUKAST	C	370,563	60,782 (16.4%)	18,331 (4.9%)	155 (0.0%)	31,936 (8.6%)	11,584 (3.1%)	247,775
NATALIZUMAB	C	85	3 (3.5%)	3 (3.5%)	0 (0.0%)	4 (4.7%)	8 (9.4%)	67

Medication	Original Category[a]	No. Patients	<1 Year of Baseline	Baseline Anti-Coagulant Use	Baseline Severe Acute Liver Injury[b]	Pre-Existing Liver/Biliary Disease[c]	Other Baseline Evidence of Liver Injury[d]	Final Sample
NEFAZODONE	C	33,492	11,519 (34.4%)	511 (1.5%)	49 (0.1%)	5,585 (16.7%)	762 (2.3%)	15,066
NILUTAMIDE	C	5,249	754 (14.4%)	339 (6.5%)	6 (0.1%)	540 (10.3%)	373 (7.1%)	3,237
NORFLOXACIN	C	1,472	296 (20.1%)	93 (6.3%)	158 (10.7%)	306 (20.8%)	22 (1.5%)	597
ORLISTAT	C	29,945	2,335 (7.8%)	1,340 (4.5%)	13 (0.0%)	3,193 (10.7%)	1,872 (6.3%)	21,192
OXAPROZIN	C	28,023	7,594 (27.1%)	311 (1.1%)	24 (0.1%)	2,836 (10.1%)	805 (2.9%)	16,453
PANTOPRAZOLE	C	919,491	96,858 (10.5%)	77,955 (8.5%)	4,924 (0.5%)	157,557 (17.1%)	31,925 (3.5%)	550,272
PENICILLIN G	C	403,922	54,329 (13.5%)	16,449 (4.1%)	761 (0.2%)	71,602 (17.7%)	12,322 (3.1%)	248,459
PHENELZINE	C	849	190 (22.4%)	18 (2.1%)	0 (0.0%)	147 (17.3%)	24 (2.8%)	470
PIOGLITAZONE	C	186,247	24,799 (13.3%)	8,928 (4.8%)	67 (0.0%)	15,368 (8.3%)	10,883 (5.8%)	126,202
PRAVASTATIN	C	844,799	112,631 (13.3%)	47,043 (5.6%)	618 (0.1%)	84,087 (10.0%)	37,614 (4.5%)	562,806
PREGABALIN	C	257,043	14,819 (5.8%)	20,611 (8.0%)	458 (0.2%)	45,372 (17.7%)	10,432 (4.1%)	165,351
PROCAINAMIDE	C	2,625	1,089 (41.5%)	484 (18.4%)	5 (0.2%)	118 (4.5%)	50 (1.9%)	879
PROCHLORPERAZINE	C	348,765	49,764 (14.3%)	25,088 (7.2%)	3,639 (1.0%)	85,303 (24.5%)	17,192 (4.9%)	167,779
PYRIMETHAMINE	C	658	132 (20.1%)	30 (4.6%)	4 (0.6%)	210 (31.9%)	44 (6.7%)	238
QUETIAPINE	C	608,240	104,880 (17.2%)	21,821 (3.6%)	1,439 (0.2%)	129,971 (21.4%)	16,944 (2.8%)	333,185
RAMIPRIL	C	48,531	15,319 (31.6%)	3,408 (7.0%)	27 (0.1%)	2,550 (5.3%)	1,143 (2.4%)	26,084
RISPERIDONE	C	372,844	69,691 (18.7%)	13,362 (3.6%)	943 (0.3%)	78,623 (21.1%)	10,844 (2.9%)	199,381
RITONAVIR	C	22,023	4,219 (19.2%)	362 (1.6%)	88 (0.4%)	12,116 (55.0%)	714 (3.2%)	4,524
ROSIGLITAZONE	C	218,052	53,118 (24.4%)	11,085 (5.1%)	101 (0.0%)	10,790 (4.9%)	9,819 (4.5%)	133,139
SORAFENIB	C	9,781	730 (7.5%)	633 (6.5%)	439 (4.5%)	6,995 (71.5%)	134 (1.4%)	850
TEMOZOLOMIDE	C	6,185	969 (15.7%)	436 (7.0%)	20 (0.3%)	1,133 (18.3%)	442 (7.1%)	3,185
TOLCAPONE	C	179	62 (34.6%)	6 (3.4%)	0 (0.0%)	7 (3.9%)	1 (0.6%)	103
TOPIRAMATE	C	348,245	50,140 (14.4%)	10,868 (3.1%)	407 (0.1%)	55,179 (15.8%)	13,187 (3.8%)	218,464
TRASTUZUMAB	C	18	5 (27.8%)	1 (5.6%)	0 (0.0%)	5 (27.8%)	0 (0.0%)	7
TRAZODONE	C	1,800,672	407,636 (22.6%)	71,004 (3.9%)	4,169 (0.2%)	272,567 (15.1%)	51,677 (2.9%)	993,619
VANCOMYCIN	C	55,603	3,380 (6.1%)	10,622 (19.1%)	1,172 (2.1%)	19,382 (34.9%)	2,003 (3.6%)	19,044
VERAPAMIL	C	165,822	67,832 (40.9%)	7,196 (4.3%)	95 (0.1%)	11,564 (7.0%)	3,715 (2.2%)	75,420
VINCRISTINE	C	336	64 (19.0%)	38 (11.3%)	3 (0.9%)	85 (25.3%)	13 (3.9%)	133
ZAFIRLUKAST	C	9,175	2,936 (32.0%)	274 (3.0%)	2 (0.0%)	492 (5.4%)	211 (2.3%)	5,260

[a] Original category as defined by Björnsson and Hoofnagle, *Hepatology* 2016;63:590-603. Medications listed in the National Institutes of Health LiverTox database were classified into categories of likelihood for causing acute liver injury based on the number of published reports of hepatotoxicity (category A, ≥50 cases; category B, 12-49 cases; category C, 4-11 cases).
[b] Baseline severe acute liver injury defined as total bilirubin >2.0 mg/dL plus either alanine aminotransferase >120 U/L or international normalized ratio ≥1.5 prior to the index date.
[c] Pre-existing liver/biliary disease defined as 1) a laboratory diagnosis of hepatitis B or hepatitis C viral infection, or 2) a liver/biliary diagnostic code (see Supplement Table 2) prior to the index date.
[d] Other baseline evidence of liver injury defined as any of the following during the baseline period: 1) two alanine aminotransferases ≥40 U/L separated by at least six months, 2) one elevated alanine aminotransferase >100 U/L, or 3) one alkaline phosphatase >172 mg/dL.
[e] Represents groups of medications.
[f] Includes amphetamine, dextroamphetamine, lisdexamfetamine, and methamphetamine.
[g] Includes mesalamine, balsalazide, and olsalazine.

eTable 7. Age-/sex-adjusted incidence rates of severe acute liver injury among initiators of 91 medications that were not included in the primary analysis because either the width of the 95% confidence interval for the severe acute liver injury incidence rate was ≥3 times the point estimate or there was <10,000 person-years of follow-up present.

Medication	Original Category[a]	No. Initiators	Age (y), Median (IQR)	Male, n (%)	No. Person-Years	No. Events	Adjusted IR[b] (95% CI), Events/10,000 Person-Years
DIDANOSINE	A	890	47.6 (41.2-54.9)	865 (97.2%)	410	2	43.4 (10.8-174.4)
NEVIRAPINE	A	1,003	49.3 (42.1-56.6)	975 (97.2%)	435	2	38.8 (9.6-156.0)
PYRAZINAMIDE	A	1,934	64.1 (55.9-74.2)	1,869 (96.6%)	252	1	24.7 (3.5-176.1)
LINEZOLID	C	15,803	65.9 (57.2-75.3)	14,965 (94.7%)	651	1	9.7 (1.4-69.2)
CYCLOPHOSPHAMIDE OR IFOSPHAMIDE	B	2,984	69.9 (62.6-76.6)	2,822 (94.6%)	812	1	7.3 (1.0-52.0)
TICLOPIDINE	A	2,137	71.8 (63.1-78.5)	2,087 (97.7%)	831	1	6.7 (0.9-47.9)
QUINIDINE	A	2,926	70.2 (61.1-77.1)	2,793 (95.5%)	963	1	6.1 (0.9-43.4)
RITONAVIR	C	4,524	50.3 (42.6-57.9)	4,279 (94.6%)	1,974	1	4.3 (0.6-30.5)
ERYTHROMYCIN	A	77,098	61.1 (51.2-71.0)	68,911 (89.4%)	3,454	2	4.1 (1.0-16.6)
NEFAZODONE	C	15,066	53.5 (46.4-59.3)	13,550 (89.9%)	4,354	2	3.7 (0.9-14.9)
ABACAVIR	C	4,347	52.8 (43.7-60.7)	4,191 (96.4%)	2,206	1	3.6 (0.5-26.0)
TAMOXIFEN	B	5,539	59.3 (48.7-70.8)	2,412 (43.5%)	2,543	1	3.6 (0.5-25.6)
RIFAMPIN	A	25,327	61.6 (51.8-70.5)	23,568 (93.1%)	3,744	2	3.4 (0.9-13.8)
ITRACONAZOLE	B	15,377	59.8 (48.5-69.3)	14,220 (92.5%)	2,169	1	3.2 (0.5-22.9)
CHLORZOXAZONE	B	23,328	57.4 (47.6-69.2)	21,242 (91.1%)	2,425	1	3.1 (0.4-21.9)
DICLOXACILLIN	B	78,771	61.2 (51.7-71.4)	73,116 (92.8%)	2,682	1	2.5 (0.4-18.1)
PENICILLIN G	C	248,459	56.2 (45.5-65.2)	222,982 (89.7%)	7,447	2	2.0 (0.5-8.2)
HYDROXYUREA	C	7,826	74.0 (66.5-81.0)	7,555 (96.5%)	3,286	1	1.6 (0.2-11.6)
HALOPERIDOL	B	41,756	64.6 (50.4-79.6)	38,154 (91.4%)	8,415	2	1.6 (0.4-6.3)
SULFASALAZINE	A	26,335	62.6 (52.9-70.7)	23,409 (88.9%)	8,732	2	1.6 (0.4-6.2)
CIMETIDINE	B	22,115	61.7 (52.1-72.1)	20,141 (91.1%)	5,574	1	1.2 (0.2-8.4)
LABETALOL	C	19,258	64.5 (54.3-74.2)	16,926 (87.9%)	7,620	1	0.8 (0.1-5.9)
ETANERCEPT	B	18,223	60.6 (50.9-68.1)	15,958 (87.6%)	9,254	1	0.8 (0.1-5.6)
AURANOFIN OR GOLD PRODUCTS[c]	A	149	63.6 (56.6-72.0)	136 (91.3%)	46	0	..[d]
BUSULFAN	A	60	73.9 (68.4-80.4)	57 (95.0%)	17	0	..[d]
DANTROLENE	A	1,744	57.1 (47.2-66.7)	1,530 (87.7%)	451	0	..[d]
EFAVIRENZ	A	9,233	76.9 (70.3-82.0)	9,216 (99.8%)	2,453	0	..[d]
FLUTAMIDE	A	1,114	55.4 (43.4-64.0)	992 (89.0%)	151	0	..[d]
INFLIXIMAB	A	3,673	49.7 (40.8-57.9)	2,790 (76.0%)	1,858	0	..[d]
INTERFERON-ß	A	2,843	62.0 (38.5-74.6)	1,772 (62.3%)	1,065	0	..[d]
METHYLDOPA	A	1,942	64.0 (51.4-75.1)	1,581 (81.4%)	607	0	..[d]
PROPYLTHIOURACIL	A	17	61.8 (58.2-74.8)	14 (82.4%)	0	0	..[d]
TELITHROMYCIN	A	38	67.8 (56.9-72.9)	35 (92.1%)	6	0	..[d]

Medication	Original Category[a]	No. Initiators	Age (y), Median (IQR)	Male, n (%)	No. Person-Years	No. Events	Adjusted IR[b] (95% CI), Events/10,000 Person-Years
THIOGUANINE	A	9	62.2 (39.7-68.0)	9 (100.0%)	1	0	..[d]
ASPARAGINASE OR PEGASPARGASE	B	22,281	43.0 (31.7-57.5)	18,973 (85.2%)	214	0	..[d]
CEFTRIAXONE	B	111	70.3 (58.3-78.1)	105 (94.6%)	45	0	..[d]
CHLORPROPAMIDE	B	36	56.8 (49.6-69.0)	36 (100.0%)	1	0	..[d]
CLOXACILLIN	B	2,418	53.7 (44.3-61.1)	2,143 (88.6%)	1,061	0	..[d]
CLOZAPINE	B	152	69.8 (59.2-76.2)	133 (87.5%)	18	0	..[d]
DOXORUBICIN	B	12	66.8 (60.0-76.1)	12 (100.0%)	2	0	..[d]
ETHIONAMIDE	B	33	53.4 (45.4-67.9)	31 (93.9%)	8	0	..[d]
FELBAMATE	B	3,366	70.2 (62.6-77.9)	3,242 (96.3%)	1,770	0	..[d]
IMATINIB	B	21,551	62.8 (51.0-73.7)	18,559 (86.1%)	6,669	0	..[d]
IMIPRAMINE	B	42	69.8 (58.4-77.3)	39 (92.9%)	9	0	..[d]
IRINOTECAN	B	12,959	64.8 (56.4-71.8)	10,968 (84.6%)	5,535	0	..[d]
LEFLUNOMIDE	B	1,767	76.3 (70.7-81.3)	1,741 (98.5%)	214	0	..[d]
MELPHALAN	B	16	68.6 (58.7-77.8)	16 (100.0%)	0	0	..[d]
OXACILLIN	B	65	68.0 (60.9-75.0)	64 (98.5%)	11	0	..[d]
OXALIPLATIN	B	401	57.5 (48.0-67.9)	362 (90.3%)	48	0	..[d]
PENICILLAMINE	B	3,929	61.2 (51.0-72.5)	3,554 (90.5%)	1,095	0	..[d]
PHENOBARBITAL	B	5,350	73.5 (66.2-80.0)	5,185 (96.9%)	2,302	0	..[d]
PROPAFENONE	B	312	62.8 (54.0-71.3)	296 (94.9%)	8	0	..[d]
THIABENDAZOLE	B	1,188	58.3 (51.4-71.0)	1,100 (92.6%)	312	0	..[d]
THIORIDAZINE	B	2,618	67.2 (60.4-73.0)	2,503 (95.6%)	602	0	..[d]
VORICONAZOLE	B	2,783	48.7 (40.7-55.8)	2,409 (86.6%)	765	0	..[d]
ZIDOVUDINE	B	370	71.3 (62.9-78.2)	336 (90.8%)	183	0	..[d]
ACEBUTOLOL	C	9,643	64.2 (55.0-71.6)	9,047 (93.8%)	2,935	0	..[d]
ACITRETIN	C	3,443	54.6 (39.0-66.1)	2,831 (82.2%)	100	0	..[d]
ALBENDAZOLE	C	424	61.7 (51.8-70.8)	384 (90.6%)	108	0	..[d]
ANAKINRA	C	18,689	37.9 (31.6-48.9)	14,851 (79.5%)	4,876	0	..[d]
ATOMOXETINE	C	199	69.9 (63.6-76.8)	185 (93.0%)	71	0	..[d]
BOSENTAN	C	6,983	36.8 (31.6-45.8)	3,795 (54.3%)	1,817	0	..[d]
CLOMIPHENE	C	29	71.2 (59.3-76.1)	28 (96.6%)	4	0	..[d]
CYTARABINE	C	4,237	59.1 (51.1-69.9)	3,912 (92.3%)	880	0	..[d]
DIFLUNISAL	C	432	69.6 (59.6-77.5)	404 (93.5%)	181	0	..[d]
DISOPYRAMIDE	C	3,353	67.7 (62.1-73.7)	3,228 (96.3%)	376	0	..[d]
ETOPOSIDE	C	7	69.2 (42.5-79.6)	4 (57.1%)	1	0	..[d]
FLAVOCOXID	C	1,516	67.8 (61.2-75.1)	1,464 (96.6%)	156	0	..[d]
FLUOROURACIL	C	601	70.8 (63.5-76.7)	595 (99.0%)	150	0	..[d]
GEFITINIB	C	218	71.5 (64.2-76.9)	218 (100.0%)	18	0	..[d]
GEMCITABINE	C	4,567	36.9 (29.8-51.7)	3,267 (71.5%)	1,445	0	..[d]

Medication	Original Category[a]	No. Initiators	Age (y), Median (IQR)	Male, n (%)	No. Person-Years	No. Events	Adjusted IR[b] (95% CI), Events/10,000 Person-Years
ISOTRETINOIN	C	1,867	55.9 (46.6-65.5)	1,647 (88.2%)	324	0	..[d]
KETOPROFEN	C	986	63.7 (54.7-73.0)	926 (93.9%)	203	0	..[d]
METHOXSALEN	C	3,423	70.0 (61.2-77.0)	3,314 (96.8%)	1,265	0	..[d]
MEXILETINE	C	2,310	71.9 (64.9-79.2)	2,270 (98.3%)	75	0	..[d]
MITOMYCIN	C	67	45.9 (39.4-52.8)	46 (68.7%)	9	0	..[d]
NATALIZUMAB	C	3,237	77.0 (70.1-82.6)	3,237 (100.0%)	1,039	0	..[d]
NILUTAMIDE	C	597	68.3 (58.4-77.0)	559 (93.6%)	26	0	..[d]
NORFLOXACIN	C	21,192	55.2 (46.2-62.4)	15,529 (73.3%)	5,373	0	..[d]
ORLISTAT	C	16,453	57.2 (48.5-67.7)	15,088 (91.7%)	4,396	0	..[d]
OXAPROZIN	C	470	55.5 (44.9-63.9)	404 (86.0%)	130	0	..[d]
PHENELZINE	C	879	73.3 (66.7-78.1)	873 (99.3%)	251	0	..[d]
PROCAINAMIDE	C	238	55.7 (44.9-65.0)	217 (91.2%)	40	0	..[d]
PYRIMETHAMINE	C	850	68.1 (62.2-76.4)	829 (97.5%)	224	0	..[d]
SORAFENIB	C	3,185	66.0 (58.3-73.0)	3,070 (96.4%)	549	0	..[d]
TEMOZOLOMIDE	C	103	70.2 (64.8-75.8)	102 (99.0%)	44	0	..[d]
TOLCAPONE	C	7	53.3 (49.2-61.5)	1 (14.3%)	1	0	..[d]
TRASTUZUMAB	C	19,044	69.7 (60.0-78.6)	17,122 (89.9%)	1,166	0	..[d]
VANCOMYCIN	C	133	69.9 (59.3-77.2)	130 (97.7%)	18	0	..[d]
VINCRISTINE	C	5,260	66.3 (55.0-75.5)	4,611 (87.7%)	2,017	0	..[d]
ZAFIRLUKAST	C	149	63.6 (56.6-72.0)	136 (91.3%)	46	0	..[d]

Abbreviations: CI=confidence interval; IQR=interquartile range; IR=incidence rate

[a] Original category as defined by Björnsson and Hoofnagle, *Hepatology* 2016;63:590-603. Medications listed in the National Institutes of Health LiverTox database were classified into categories of likelihood for causing acute liver injury based on the number of published reports of hepatotoxicity (category A, ≥50 cases; category B, 12-49 cases; category C, 4-11 cases).

[b] Incidence rates adjusted for age and sex.

[c] Represents groups of medications.

[d] No events were observed among initiators of this medication. Thus, no unadjusted or adjusted incidence rates were calculated.

eTable 8. Incidence rates of severe acute liver injury for Group 3-5 medications with ≥4 published reports of hepatotoxicity (categories A, B, C).

Medication	Original Category[a]	No. Initiators	Age (y), Median (IQR)	Male, n (%)	No. Person-Years	No. Events	Adjusted IR[b] (95% CI), Events/10,000 Person-Years
**GROUP 3 (3.0-4.9 events/10,000 person-years)**							
CYPROHEPTADINE	C	65,239	58.3 (44.9-70.6)	57,982 (88.9%)	14,625	10	4.96 (2.6-9.3)
ESTROGENS OR PROGESTINS[c]	A	179,681	56.8 (37.7-75.7)	91,232 (50.8%)	48,270	24	4.9 (3.3-7.4)
METHIMAZOLE (THIAMAZOLE)	B	11,865	64.4 (53.1-73.8)	9,884 (83.3%)	4,546	3	4.4 (1.4-13.6)
AMIODARONE	A	96,808	73.9 (67.0-80.7)	95,618 (98.8%)	32,303	25	4.0 (2.6-6.0)
AZITHROMYCIN	B	1,303,960	60.9 (48.9-70.3)	1,163,672 (89.2%)	24,105	14	4.0 (2.3-6.8)
HYDRALAZINE OR ISOSORBIDE DINITRATE	A	158,674	70.6 (62.9-78.7)	153,010 (96.4%)	63,745	43	3.8 (2.7-5.2)
CEPHALEXIN	C	1,147,378	62.5 (51.9-71.9)	1,053,891 (91.9%)	33,037	18	3.7 (2.3-5.9)
**GROUP 4 (1.0-2.9 events/10,000 person-years)**							
DOXYCYCLINE	C	1,023,900	62.4 (50.5-71.3)	922,978 (90.1%)	58,035	23	2.7 (1.8-4.2)
CARBAMAZEPINE	A	68,500	58.0 (46.8-68.9)	60,661 (88.6%)	19,733	7	2.6 (1.2-5.5)
LANSOPRAZOLE OR DEXLANSOPRAZOLE	C	134,492	65.9 (54.4-74.9)	125,021 (93.0%)	44,590	18	2.6 (1.6-4.2)
CLINDAMYCIN	B	480,895	60.6 (50.2-69.3)	429,305 (89.3%)	14,492	5	2.5 (1.0-5.9)
NITROFURANTOIN	A	219,951	67.0 (54.8-76.0)	152,562 (69.4%)	10,896	4	2.3 (0.9-6.1)
PHENYTOIN OR FOSPHENYTOIN	A	31,844	66.0 (55.6-76.3)	30,452 (95.6%)	11,500	4	2.1 (0.8-5.7)
QUININE	B	49,238	70.4 (60.0-77.6)	47,261 (96.0%)	16,697	6	2.1 (0.9-4.7)
IRBESARTAN	C	79,339	69.9 (60.0-76.8)	76,544 (96.5%)	45,193	16	2.1 (1.3-3.5)
RAMIPRIL	C	26,084	70.5 (61.4-77.5)	25,478 (97.7%)	14,030	5	2.1 (0.8-5.0)
ADALIMUMAB	C	30,340	59.1 (46.8-67.7)	26,295 (86.7%)	15,390	4	2.0 (0.7-5.3)
RANITIDINE	B	912,024	61.7 (50.2-71.8)	826,157 (90.6%)	287,636	84	1.9 (1.5-2.5)
AMOXICILLIN	B	1,092,193	60.4 (49.3-69.8)	984,698 (90.2%)	33,475	9	1.9 (1.0-3.7)
ENALAPRIL	B	101,171	69.3 (60.1-77.5)	98,353 (97.2%)	53,213	17	1.9 (1.1-3.0)
ALLOPURINOL	A	324,944	67.7 (59.4-75.6)	319,514 (98.3%)	158,458	49	1.8 (1.4-2.5)
PIROXICAM	B	134,939	57.6 (48.2-67.0)	121,920 (90.4%)	40,917	9	1.6 (0.8-3.1)
RISPERIDONE	C	199,381	56.6 (44.6-70.3)	179,219 (89.9%)	60,785	13	1.5 (0.9-2.7)
AMITRIPTYLINE	B	379,687	56.2 (44.7-66.1)	327,028 (86.1%)	105,547	21	1.5 (1.0-2.4)
TRAZODONE	C	993,619	56.9 (43.2-67.7)	877,287 (88.3%)	275,521	53	1.4 (1.1-1.9)
OMEPRAZOLE OR ESOMEPRAZOLE	B	2,104,361	63.8 (52.8-73.9)	1,941,156 (92.2%)	892,075	198	1.4 (1.2-1.7)
FAMOTIDINE	C	276,349	64.0 (50.5-72.7)	238,542 (86.3%)	81,739	17	1.3 (0.8-2.2)
LEVETIRACETAM	C	63,268	66.2 (55.4-75.4)	58,253 (92.1%)	23,307	5	1.3 (0.6-3.2)
SULINDAC	A	155,519	58.8 (48.6-68.8)	141,261 (90.8%)	33,910	6	1.3 (0.6-2.8)
MESALAMINE[d]	C	27,941	64.0 (50.6-73.2)	25,374 (90.8%)	10,441	2	1.2 (0.3-5.0)
OLANZAPINE	B	98,325	55.7 (43.8-69.4)	87,821 (89.3%)	29,886	5	1.2 (0.5-3.0)
INDOMETHACIN	C	302,459	59.9 (49.6-68.7)	282,558 (93.4%)	29,322	5	1.2 (0.5-3.0)
GLYBURIDE (GLIBENCLAMIDE)	B	166,209	65.1 (57.7-74.2)	161,212 (97.0%)	89,497	17	1.2 (0.7-1.9)

Medication	Original Category[a]	No. Initiators	Age (y), Median (IQR)	Male, n (%)	No. Person-Years	No. Events	Adjusted IR[b] (95% CI), Events/10,000 Person-Years
CLOPIDOGREL	B	636,233	71.4 (64.0-78.6)	623,472 (98.0%)	350,863	74	1.2 (0.9-1.5)
PREGABALIN	C	165,351	62.0 (51.3-70.0)	142,408 (86.1%)	61,718	10	1.1 (0.6-2.1)
MIRTAZAPINE	C	512,403	58.1 (44.0-69.7)	461,055 (90.0%)	155,849	25	1.1 (0.8-1.7)
NAPROXEN	B	1,404,591	56.3 (43.8-66.4)	1,254,033 (89.3%)	245,252	37	1.1 (0.8-1.6)
PAROXETINE	B	249,256	56.3 (43.0-67.7)	218,123 (87.5%)	88,672	13	1.1 (0.6-1.9)
METHYLPHENIDATE OR DEXMETHYLPHENIDATE	C	81,586	43.9 (32.7-58.2)	66,950 (82.1%)	27,650	3	1.1 (0.3-3.3)
GLIPIZIDE	C	500,523	66.5 (59.2-74.3)	481,992 (96.3%)	272,608	47	1.0 (0.8-1.4)
SERTRALINE	B	828,219	56.9 (41.4-68.6)	729,176 (88.0%)	313,499	44	1.0 (0.8-1.4)
LAMOTRIGINE	B	117,942	48.8 (36.5-60.2)	90,808 (77.0%)	45,088	5	1.0 (0.4-2.5)
CITALOPRAM OR ESCITALOPRAM	C	844,476	57.5 (43.5-69.3)	742,613 (87.9%)	314,401	44	1.0 (0.7-1.4)
DILTIAZEM	C	199,794	69.8 (60.7-78.0)	190,111 (95.2%)	98,831	17	1.0 (0.6-1.6)
**GROUP 5 (<1.0 event/10,000 person-years)**							
ATORVASTATIN	A	1,543,771	65.8 (57.3-72.0)	1,457,503 (94.4%)	789,334	124	0.99 (0.8-1.2)
FLUVASTATIN	B	83,675	65.7 (57.9-74.2)	80,025 (95.6%)	37,870	6	0.99 (0.4-2.2)
LISINOPRIL	B	1,565,009	65.1 (56.5-74.1)	1,492,448 (95.4%)	829,033	128	0.97 (0.8-1.2)
LOVASTATIN	B	281,703	65.6 (57.0-74.3)	269,335 (95.6%)	141,138	22	0.97 (0.6-1.5)
PANTOPRAZOLE	C	550,272	65.1 (53.2-73.5)	492,381 (89.5%)	231,547	35	0.97 (0.7-1.4)
GLIMEPIRIDE	C	38,317	68.9 (60.1-74.1)	36,400 (95.0%)	19,176	3	0.9 (0.3-3.0)
IBUPROFEN	A	1,479,820	54.6 (41.1-65.1)	1,302,237 (88.0%)	191,047	21	0.9 (0.6-1.3)
SIMVASTATIN	A	1,567,669	63.2 (54.9-72.8)	1,488,844 (95.0%)	834,140	111	0.9 (0.7-1.1)
VERAPAMIL	C	75,420	59.2 (46.5-70.5)	65,035 (86.2%)	33,473	4	0.8 (0.3-2.2)
DICLOFENAC	A	567,401	56.4 (44.7-65.8)	500,659 (88.2%)	122,557	13	0.8 (0.5-1.4)
ETODOLAC	C	599,148	57.4 (47.0-67.0)	543,326 (90.7%)	127,177	14	0.8 (0.5-1.4)
GABAPENTIN	C	1,394,098	61.9 (50.6-71.1)	1,260,443 (90.4%)	419,066	48	0.8 (0.6-1.0)
ROSIGLITAZONE	C	133,139	66.6 (58.2-74.4)	130,183 (97.8%)	75,718	9	0.7 (0.4-1.4)
CELECOXIB	B	140,508	60.6 (49.4-70.8)	121,732 (86.6%)	39,466	4	0.7 (0.3-1.9)
FLUOXETINE	C	420,597	54.2 (39.6-65.1)	354,870 (84.4%)	159,871	14	0.7 (0.4-1.2)
LOSARTAN	C	584,576	68.0 (60.0-75.3)	551,935 (94.4%)	334,509	39	0.7 (0.5-1.0)
LEVOCETIRIZINE OR CETIRIZINE	C	808,461	60.2 (46.4-69.4)	686,286 (84.9%)	229,431	22	0.7 (0.5-1.1)
QUETIAPINE	C	333,185	56.1 (43.0-68.2)	297,253 (89.2%)	105,147	10	0.7 (0.4-1.3)
MONTELUKAST	C	247,775	62.3 (49.0-71.7)	207,118 (83.6%)	103,942	10	0.7 (0.4-1.2)
DULOXETINE	B	371,864	57.5 (44.4-68.1)	310,439 (83.5%)	141,362	12	0.7 (0.4-1.2)
VENLAFAXINE OR DEXVENLAFAXINE	B	375,843	53.5 (40.0-64.0)	311,482 (82.9%)	137,264	11	0.7 (0.4-1.2)
BUPROPION	C	818,591	53.9 (40.8-62.8)	714,446 (87.3%)	237,022	18	0.6 (0.4-1.0)
ALFUZOSIN	C	96,516	70.1 (64.9-77.5)	96,200 (99.7%)	43,850	5	0.6 (0.3-1.5)
VALPROATE	A	230,237	52.1 (38.7-63.3)	215,936 (88.5%)	76,549	6	0.6 (0.3-1.4)
METFORMIN	B	798,918	64.1 (56.1-71.4)	750,342 (93.9%)	415,935	40	0.6 (0.4-0.9)
ROSUVASTATIN	B	599,708	65.5 (59.1-73.0)	568,282 (94.8%)	320,330	32	0.6 (0.4-0.9)

Medication	Original Category[a]	No. Initiators	Age (y), Median (IQR)	Male, n (%)	No. Person-Years	No. Events	Adjusted IR[b] (95% CI), Events/10,000 Person-Years
ANDROGENIC STEROIDS[c]	A	127,937	59.9 (50.4-66.6)	126,880 (99.2%)	57,806	5	0.6 (0.3-1.5)
PRAVASTATIN	C	562,806	65.5 (58.5-73.7)	529,419 (94.1%)	279,100	28	0.6 (0.4-0.9)
AMLODIPINE	C	1,186,177	67.6 (59.5-76.3)	1,128,097 (95.1%)	662,083	68	0.6 (0.5-0.8)
METHOTREXATE	A	56,115	63.4 (53.5-71.4)	48,821 (87.0%)	25,134	2	0.5 (0.1-2.2)
GEMFIBROZIL	C	329,067	60.9 (53.0-69.5)	317,133 (96.4%)	150,507	11	0.5 (0.3-0.9)
NIFEDIPINE	B	162,333	67.6 (58.3-75.9)	153,224 (94.4%)	80,493	6	0.4 (0.2-1.0)
FENOFIBRATE	B	81,251	62.4 (55.3-69.2)	82,031 (96.6%)	45,060	3	0.4 (0.1-1.4)
MELOXICAM	C	1,006,651	58.4 (45.8-67.5)	886,004 (88.0%)	266,967	16	0.4 (0.3-0.7)
EZETIMIBE	C	164,602	67.3 (59.5-73.9)	157,046 (95.4%)	84,786	6	0.4 (0.2-1.0)
TOPIRAMATE	C	218,464	48.5 (37.3-59.5)	160,502 (73.5%)	67,429	3	0.4 (0.1-1.3)
TERBINAFINE	B	283,187	60.9 (50.5-69.5)	262,266 (92.6%)	54,510	3	0.4 (0.1-1.2)
AMPHETAMINE[e]	C	69,381	37.1 (30.8-48.3)	54,758 (78.9%)	32,364	1	0.4 (0.0-2.5)
PIOGLITAZONE	C	126,202	66.3 (59.6-73.2)	121,842 (96.5%)	69,919	4	0.4 (0.1-0.9)
ACARBOSE	B	50,400	65.8 (59.5-73.1)	48,778 (96.8%)	20,253	1	0.3 (0.0-2.2)
MINOCYCLINE	A	97,526	61.4 (47.4-69.9)	84,792 (86.9%)	14,161	0	..[f]
CANDESARTAN	C	18,266	70.8 (61.4-77.3)	17,433 (95.4%)	11,037	0	..[f]

Abbreviations: CI=confidence interval; IQR=interquartile range; IR=incidence rate

[a] Original category as defined by Björnsson and Hoofnagle, *Hepatology* 2016;63:590-603. Medications listed in the National Institutes of Health LiverTox database were classified into categories of likelihood for causing acute liver injury based on the number of published reports of hepatotoxicity (category A, ≥50 cases; category B, 12-49 cases; category C, 4-11 cases).

[b] Incidence rates adjusted for age and sex.

[c] Represents a group of medications.

[d] Includes mesalamine, balsalazide, and olsalazine.

[e] Includes amphetamine, dextroamphetamine, lisdexamfetamine, and methamphetamine.

[f] No events were observed among initiators of this medication. Thus, no unadjusted or adjusted incidence rates were calculated.

eTable 9. Incidence rates of severe acute liver injury, by sex, for medications with rates that could be estimated with sufficient precision.

Medication	Group in Primary Analysis	Males					Females				
		No. Initiators	No. Person-Years	No. Events	Adjusted IR[a] (95% CI), Events/10,000 Person-Years	Group	No. Initiators	No. Person-Years	No. Events	Adjusted IR[a] (95% CI), Events/10,000 Person-Years	Group
ESTROGENS OR PROGESTINS[b]	3	91,232	18,018	24	10.4 (6.9-15.5)	1	88,449	30,252	0	..[c]	5
RANITIDINE	4	826,157	265,716	83	2.9 (2.3-3.6)	4	85,867	21,920	1	0.4 (0.1-2.9)	5
AMITRIPTYLINE	4	327,028	91,956	20	2.2 (1.4-3.5)	4	52,659	13,591	1	0.7 (0.1-5.2)	5
OMEPRAZOLE OR ESOMEPRAZOLE	4	1,941,156	837,894	193	2.1 (1.8-2.4)	4	163,205	54,181	5	0.8 (0.3-1.9)	5
TRAZODONE	4	877,287	245,666	50	2.1 (1.6-2.7)	4	116,332	29,855	3	0.98 (0.3-3.1)	5
PAROXETINE	4	218,123	78,319	13	1.7 (1.0-2.9)	4	31,133	10,352	0	..[c]	5
CLOPIDOGREL	4	623,472	344,556	71	1.7 (1.3-2.1)	4	12,761	6,307	3	2.8 (0.8-9.2)	4
NAPROXEN	4	1,254,033	222,983	34	1.6 (1.1-2.2)	4	150,558	22,269	3	1.3 (0.4-4.1)	4
MIRTAZAPINE	4	461,055	141,992	22	1.5 (1.0-2.3)	4	51,348	13,857	3	1.9 (0.6-6.1)	4
CITALOPRAM OR ESCITALOPRAM	4	742,613	278,449	42	1.5 (1.1-2.0)	4	101,863	35,952	2	0.5 (0.1-2.2)	5
SERTRALINE	4	729,176	278,073	41	1.5 (1.1-2.0)	4	99,043	35,426	3	0.8 (0.3-2.6)	5
ATORVASTATIN	5	1,457,503	749,553	119	1.4 (1.2-1.7)	4	86,268	39,781	5	0.9 (0.4-2.3)	5
LISINOPRIL	5	1,492,448	794,836	125	1.4 (1.2-1.7)	4	72,561	34,197	3	0.7 (0.2-2.1)	5
PANTOPRAZOLE	5	492,381	210,433	32	1.4 (1.0-1.9)	4	57,891	21,115	3	1.2 (0.4-3.7)	4
LAMOTRIGINE	4	90,808	35,008	4	1.3 (0.5-3.6)	4	27,134	10,080	1	1.1 (0.1-7.5)	4
SIMVASTATIN	5	1,488,844	796,535	109	1.3 (1.0-1.5)	4	78,825	37,605	2	0.4 (0.1-1.7)	5
IBUPROFEN	5	1,302,237	170,620	19	1.2 (0.8-1.9)	4	177,583	20,427	2	1.0 (0.3-4.0)	4
GABAPENTIN	5	1,260,443	384,095	48	1.2 (0.9-1.6)	4	133,655	34,971	0	..[c]	5
DICLOFENAC	5	500,659	110,353	12	1.1 (0.6-2.0)	4	66,742	12,204	1	0.8 (0.1-5.5)	5
LEVOCETIRIZINE OR CETIRIZINE	5	686,286	195,897	22	1.1 (0.7-1.7)	4	122,175	33,534	0	..[c]	5
LOSARTAN	5	551,935	317,594	39	1.1 (0.8-1.4)	4	32,641	16,915	0	..[c]	5
FLUOXETINE	5	354,870	135,746	13	1.0 (0.6-1.8)	4	65,727	24,125	1	0.4 (0.1-3.1)	5
QUETIAPINE	5	297,253	94,321	9	0.96 (0.5-1.8)	5	35,932	10,826	1	0.9 (0.1-6.4)	5
DULOXETINE	5	310,439	117,976	11	0.96 (0.5-1.7)	5	61,425	23,386	1	0.4 (0.1-2.8)	5
VENLAFAXINE OR DEXVENLAFAXINE	5	311,482	114,536	10	0.9 (0.5-1.8)	5	64,361	22,728	1	0.4 (0.1-3.1)	5
METFORMIN	5	750,342	394,222	40	0.9 (0.7-1.3)	5	48,576	21,713	0	..[c]	5
BUPROPION	5	714,446	204,314	17	0.9 (0.6-1.5)	5	104,145	32,708	1	0.3 (0.0-2.2)	5
PRAVASTATIN	5	529,419	264,211	28	0.9 (0.6-1.4)	5	33,387	14,889	0	..[c]	5
MONTELUKAST	5	207,118	88,836	9	0.9 (0.5-1.8)	5	40,657	15,105	1	0.6 (0.1-4.2)	5
ROSUVASTATIN	5	568,282	305,723	32	0.9 (0.7-1.3)	5	31,426	14,607	0	..[c]	5
AMLODIPINE	5	1,128,097	633,967	66	0.9 (0.7-1.1)	5	58,080	28,115	2	0.5 (0.1-2.0)	5
MELOXICAM	5	886,004	238,328	15	0.6 (0.4-1.0)	5	120,647	28,638	1	0.3 (0.0-2.2)	5
TOPIRAMATE	5	160,502	49,193	2	0.5 (0.1-1.9)	5	57,962	18,236	1	0.6 (0.1-4.2)	5

Abbreviations: CI=confidence interval; IQR=interquartile range; IR=incidence rate

[a] Incidence rates adjusted for age.

[b] Represents a group of medications.

[c] No events were observed among initiators of this medication. Thus, no unadjusted or adjusted incidence rates were calculated.

eTable 10. Most common Group 1 and Group 2 medications that were additionally dispensed within 90 days of initiation of each medication of interest.

Medication	No. (%) with Additional Group 1-2 Medications	No. (%) with Top Co-Medication #1	No. (%) with Top Co-Medication #2	No. (%) with Top Co-Medication #3
Stavudine	350/750 (46.7%)	Sulfamethoxazole: 261/750 (34.8%)	Fluconazole: 115/750 (15.3%)	Levofloxacin: 31/750 (4.1%)
Erlotinib	1,275/4,356 (29.3%)	Prochlorperazine: 648/4,356 (14.9%)	Moxifloxacin: 271/4,356 (6.2%)	Clavulanate: 190/4,356 (4.4%)
Lenalidomide	1,724/8,191 (21.0%)	Prochlorperazine: 640/8,191 (7.8%)	Sulfamethoxazole: 527/8,191 (6.4%)	Clavulanate: 195/8,191 (2.4%)
Chlorpromazine	3,021/17,449 (17.3%)	Prochlorperazine: 945/17,449 (5.4%)	Clavulanate: 568/17,449 (3.3%)	Ciprofloxacin: 543/17,449 (3.1%)
Metronidazole	196,118/423,666 (46.3%)	Ciprofloxacin: 99,401/423,666 (23.5%)	Clarithromycin: 23,462/423,666 (5.5%)	Sulfamethoxazole: 21,775/423,666 (5.1%)
Prochlorperazine	34,723/167,779 (20.7%)	Ciprofloxacin: 8,525/167,779 (5.1%)	Clavulanate: 7,723/167,779 (4.6%)	Sulfamethoxazole: 7,532/167,779 (4.5%)
Isoniazid	2,716/20,476 (13.3%)	Ciprofloxacin: 586/20,476 (2.9%)	Sulfamethoxazole: 577/20,476 (2.8%)	Clavulanate: 559/20,476 (2.7%)
Moxifloxacin	55,969/376,367 (14.9%)	Clavulanate: 19,892/376,367 (5.3%)	Sulfamethoxazole: 12,134/376,367 (3.2%)	Ciprofloxacin: 11,972/376,367 (3.2%)
Azathioprine	2,612/16,033 (16.3%)	Sulfamethoxazole: 988/16,033 (6.2%)	Metronidazole: 719/16,033 (4.5%)	Ciprofloxacin: 538/16,033 (3.4%)
Levofloxacin	96,843/580,210 (16.7%)	Clavulanate: 27,289/580,210 (4.7%)	Sulfamethoxazole: 25,671/580,210 (4.4%)	Ciprofloxacin: 17,291/580,210 (3.0%)
Clarithromycin	44,076/210,356 (21.0%)	Metronidazole: 23,422/210,356 (11.1%)	Clavulanate: 6,696/210,356 (3.2%)	Ciprofloxacin: 4,327/210,356 (2.1%)
Ketoconazole	3,701/29,976 (12.3%)	Sulfamethoxazole: 793/29,976 (2.6%)	Clavulanate: 785/29,976 (2.6%)	Ciprofloxacin: 753/29,976 (2.5%)
Fluconazole	81,378/287,646 (28.3%)	Metronidazole: 19,932/287,646 (6.9%)	Sulfamethoxazole: 19,207/287,646 (6.7%)	Clavulanate: 19,187/287,646 (6.7%)
Captopril	1,687/18,863 (8.9%)	Levofloxacin: 549/18,863 (2.9%)	Clavulanate: 318/18,863 (1.7%)	Sulfamethoxazole: 315/18,863 (1.7%)
Clavulanate	117,457/1,235,143 (9.5%)	Sulfamethoxazole: 33,562/1,235,143 (2.7%)	Ciprofloxacin: 30,151/1,235,143 (2.4%)	Levofloxacin: 17,197/1,235,143 (1.4%)
Sulfamethoxazole	148,344/1,025,123 (14.5%)	Ciprofloxacin: 52,528/1,025,123 (5.1%)	Clavulanate: 41,050/1,025,123 (4.0%)	Levofloxacin: 18,316/1,025,123 (1.8%)
Ciprofloxacin	198,842/1,125,460 (17.7%)	Metronidazole: 79,864/1,125,460 (7.1%)	Sulfamethoxazole: 54,166/1,125,460 (4.8%)	Clavulanate: 36,391/1,125,460 (3.2%)

eFigure 1. Rates of severe acute liver injury for Group 1-2 initiators additionally adjusted for metabolic comorbidities. Age-/sex-adjusted incidence rates of severe acute liver injury (per 10,000 person years and with 95% confidence intervals) are reported for medications classified in Group 1 (≥10.0 events/10,000 person-years) and Group 2 (5.0-9.9 events/10,000 person-years) in the primary analysis after additionally adjusting for diabetes mellitus, hyperlipidemia, and obesity (body mass index >30 kg/m^2). Colors represent the categories of likelihood to cause severe acute liver injury based on numbers of published case reports of hepatotoxicity.

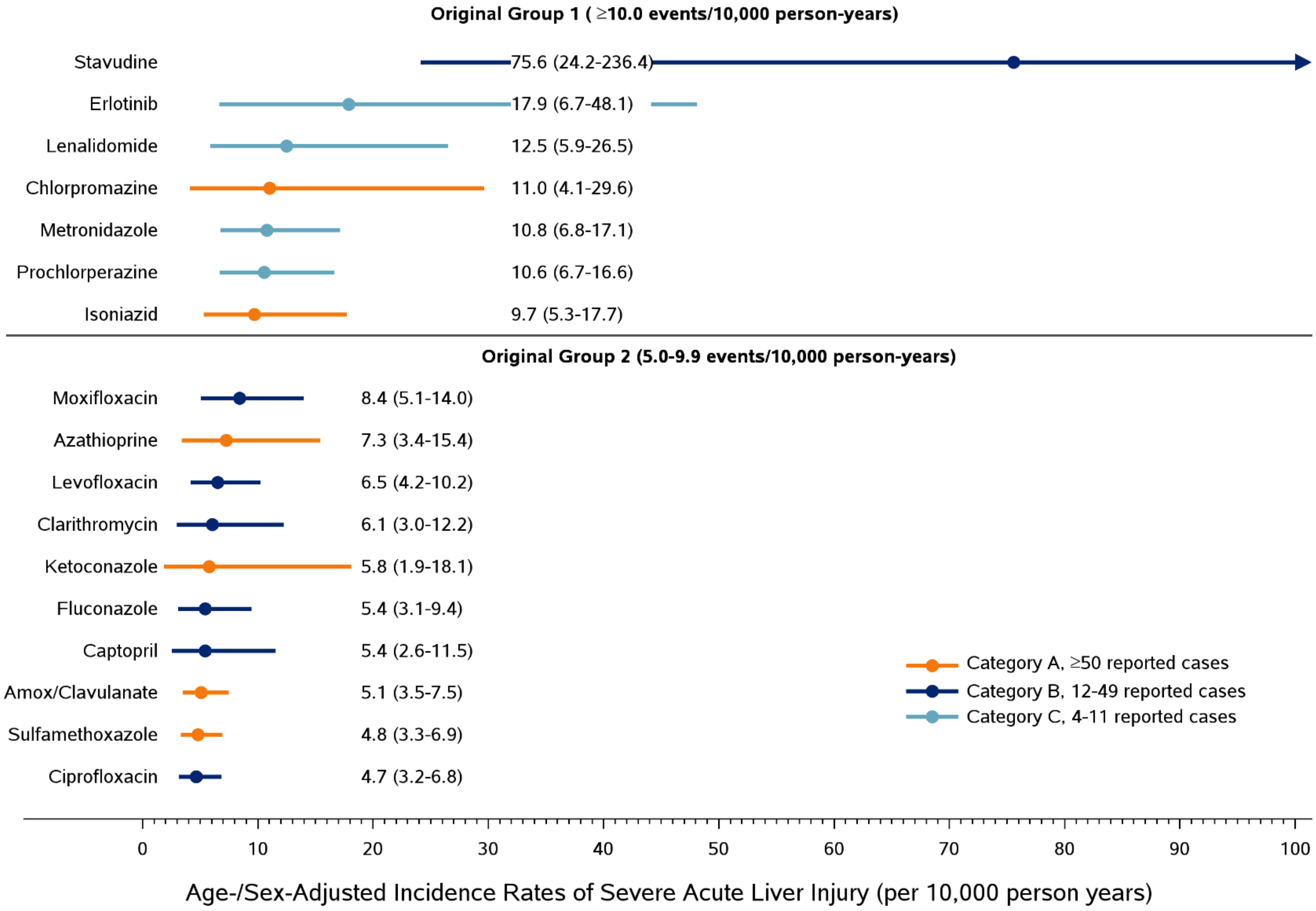

eFigure 2. Rates of severe acute liver injury for Group 3-4 initiators additionally adjusted for metabolic comorbidities. Age-/sex-adjusted incidence rates of severe acute liver injury (per 10,000 person years and with 95% confidence intervals) are reported for medications classified in Group 3 (3.0-4.9 events/10,000 person-years) and Group 4 (1.0-2.9 events/10,000 person-years) in the primary analysis after additionally adjusting for diabetes mellitus, hyperlipidemia, and obesity (body mass index >30 kg/m^2). Colors represent the categories of likelihood to cause severe acute liver injury based on numbers of published case reports of hepatotoxicity.

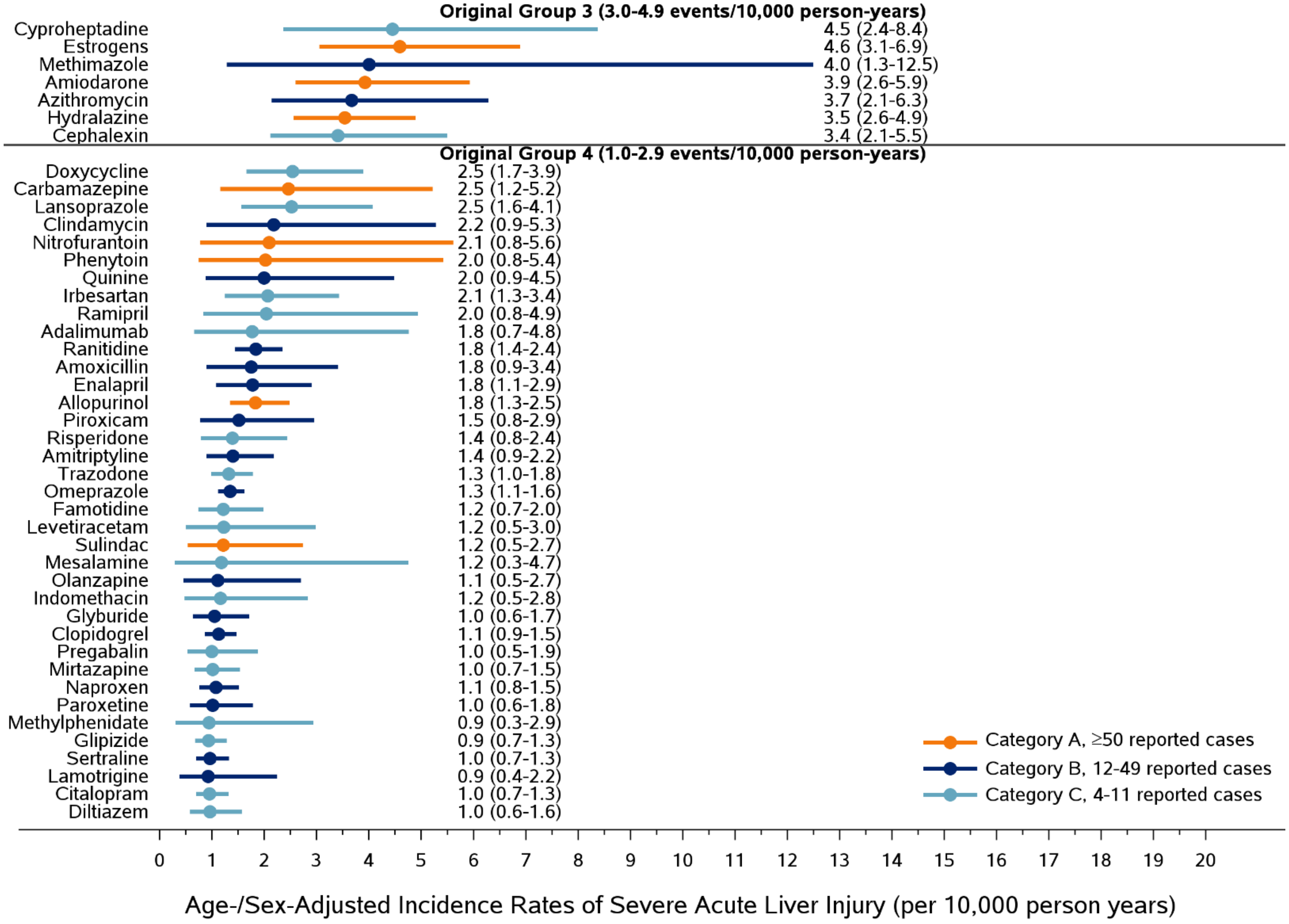

eFigure 3. Rates of severe acute liver injury for Group 5 initiators additionally adjusted for metabolic comorbidities. Age-/sex-adjusted incidence rates of severe acute liver injury (per 10,000 person years and with 95% confidence intervals) are reported for medications classified in Group 5 (<1.0 events/10,000 person-years) in the primary analysis after additionally adjusting for diabetes mellitus, hyperlipidemia, and obesity (body mass index >30 kg/m^2). Colors represent the categories of likelihood to cause severe acute liver injury based on numbers of published case reports of hepatotoxicity.

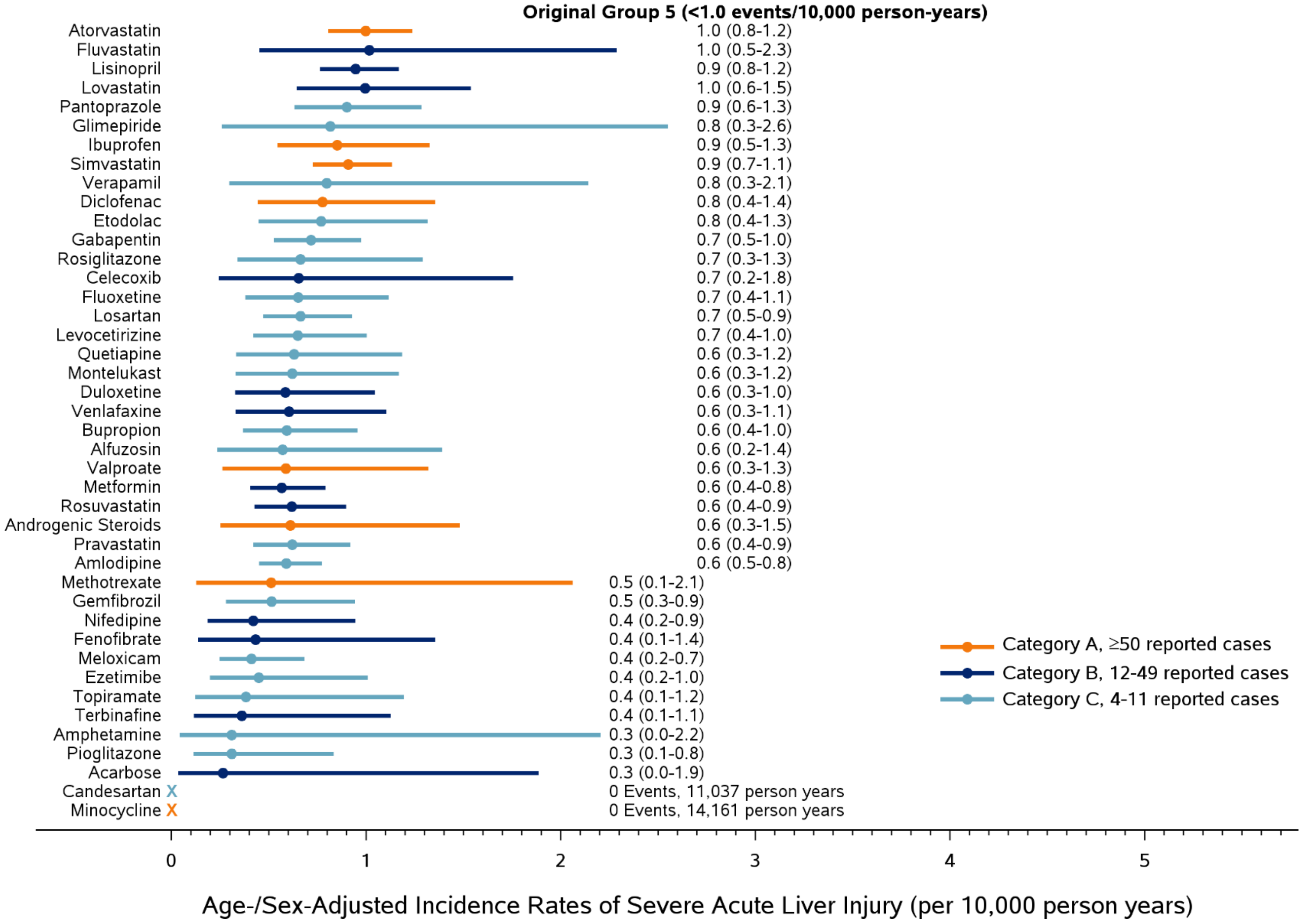

© 2024 Torgersen J et al. *JAMA Internal Medicine.*

eFigure 4. Rates of severe acute liver injury for Group 1-2 initiators <65 years of age. Age-/sex-adjusted incidence rates of severe acute liver injury (per 10,000 person years and with 95% confidence intervals) are reported for medications classified in Group 1 (≥10.0 events/10,000 person-years) and Group 2 (5.0-9.9 events/10,000 person-years) in the primary analysis after limiting each medication initiator cohort to persons <65 years of age. Colors represent the categories of likelihood to cause severe acute liver injury based on numbers of published case reports of hepatotoxicity.

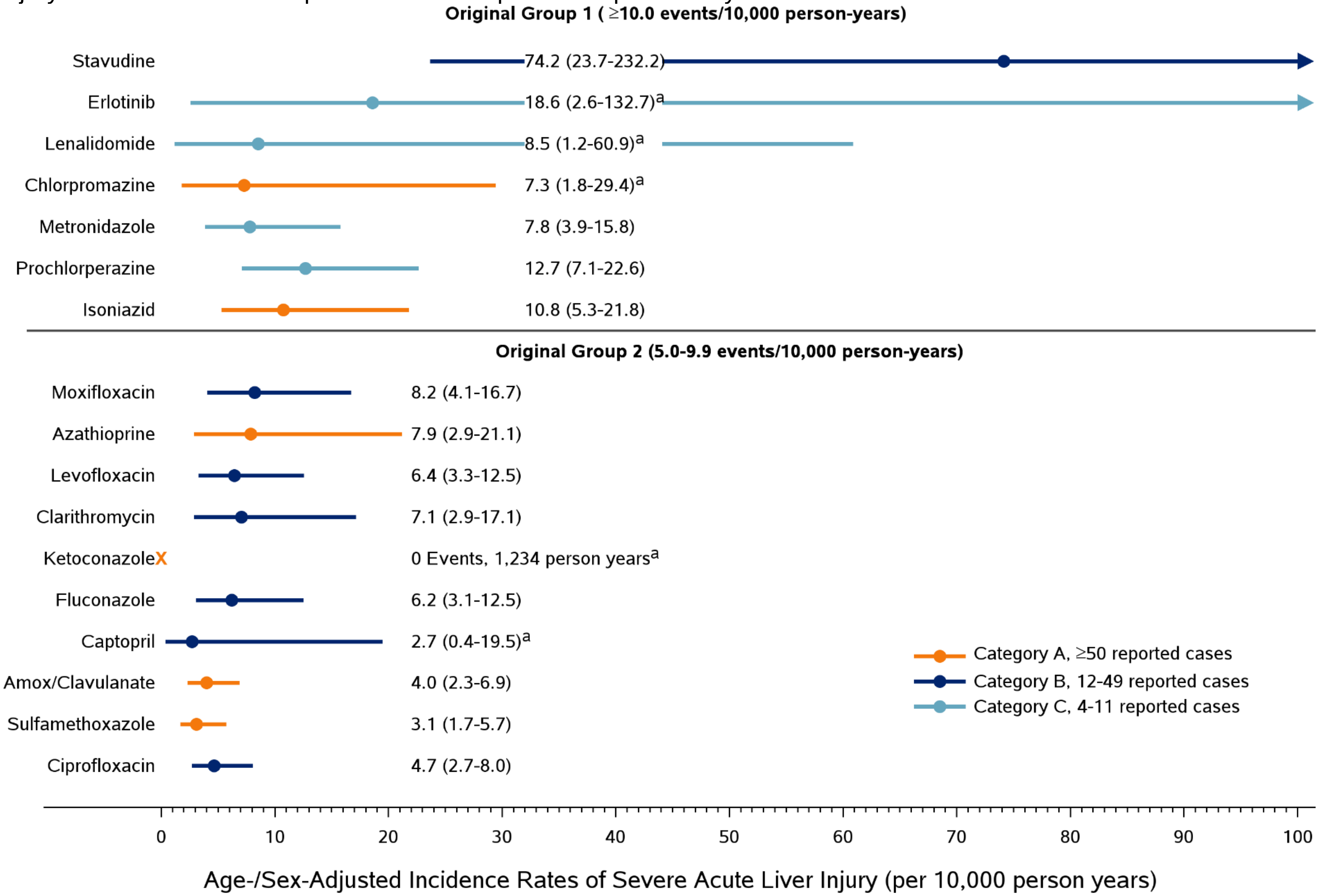

[a] After exclusion of initiators on multiple hepatotoxic medications, the 95% confidence interval of the incidence rate was too wide to meet our initial criteria for classification into Groups 1-5.

eFigure 5. Rates of severe acute liver injury for Group 3-4 initiators <65 years of age. Age-/sex-adjusted incidence rates of severe acute liver injury (per 10,000 person years and with 95% confidence intervals) are reported for medications classified in Group 3 (3.0-4.9 events/10,000 person-years) and Group 4 (1.0-2.9 events/10,000 person-years) in the primary analysis after limiting each medication initiator cohort to persons <65 years of age. Colors represent the categories of likelihood to cause severe acute liver injury based on numbers of published case reports of hepatotoxicity.

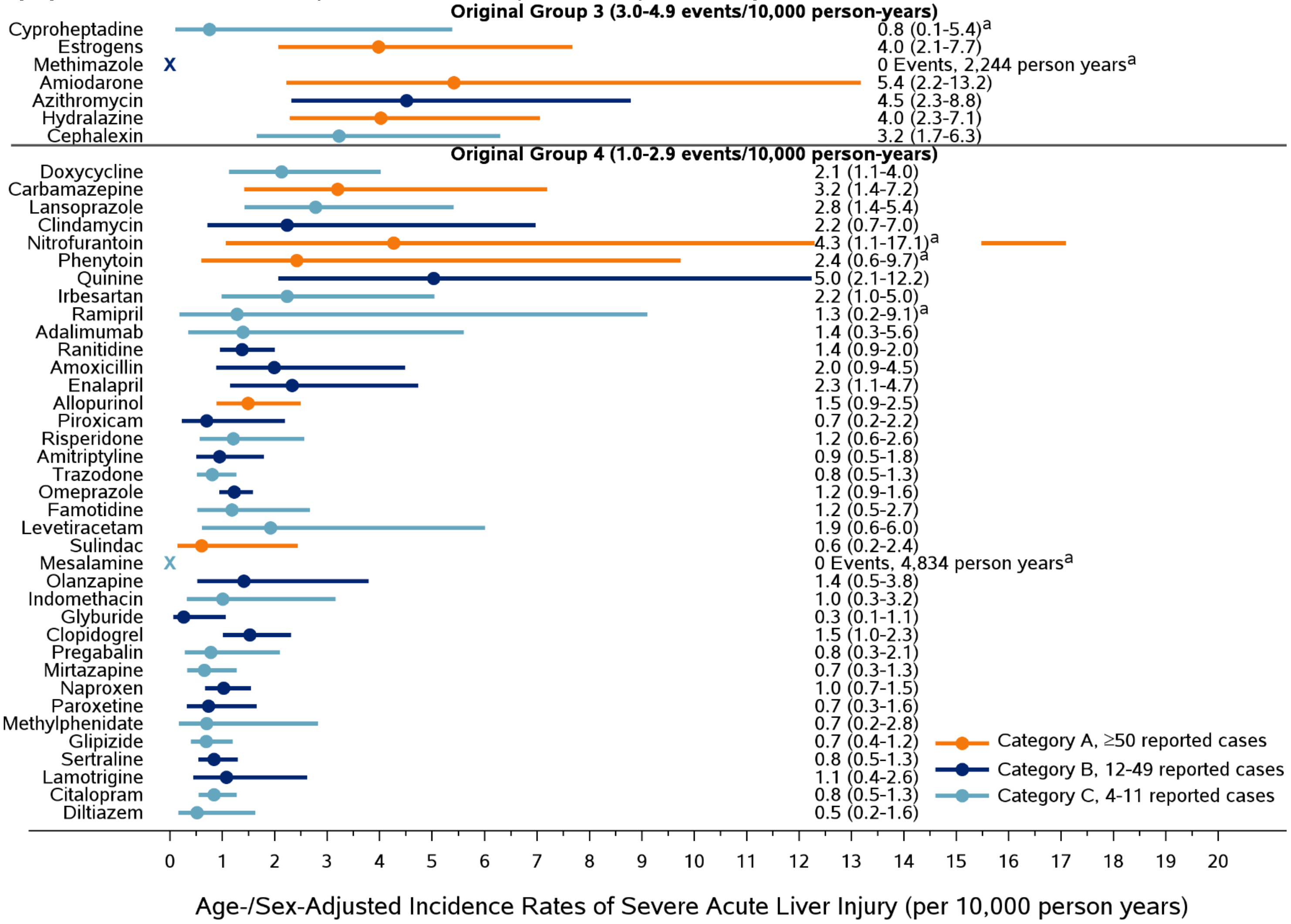

[a] After exclusion of initiators on multiple hepatotoxic medications, the 95% confidence interval of the incidence rate was too wide to meet our initial criteria for classification into Groups 1-5.

eFigure 6. Rates of severe acute liver injury for Group 5 initiators <65 years of age. Age-/sex-adjusted incidence rates of severe acute liver injury (per 10,000 person years and with 95% confidence intervals) are reported for medications classified in Group 5 (<1.0 events/10,000 person-years) in the primary analysis after limiting each medication initiator cohort to persons <65 years of age. Colors represent the categories of likelihood to cause severe acute liver injury based on numbers of published case reports of hepatotoxicity.

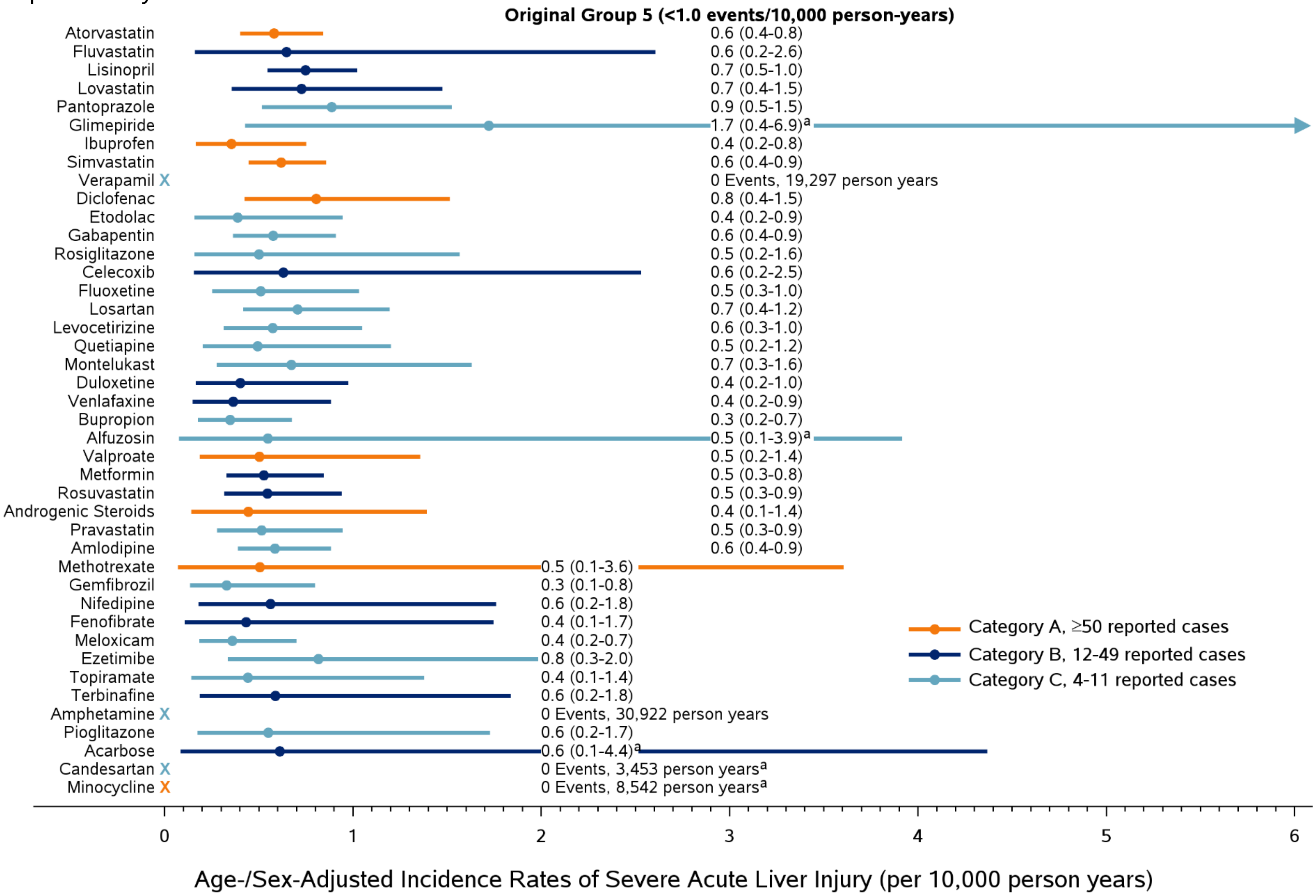

[a] After exclusion of initiators on multiple hepatotoxic medications, the 95% confidence interval of the incidence rate was too wide to meet our initial criteria for classification into Groups 1-5.

© 2024 Torgersen J et al. *JAMA Internal Medicine.*

eFigure 7. Rates of severe acute liver injury for Group 1-2 initiators additionally dispensed Group 1-2 medications. Age-/sex-adjusted incidence rates of severe acute liver injury (per 10,000 person years and with 95% confidence intervals) are reported for medications classified in Group 1 (≥10.0 events/10,000 person-years) and Group 2 (5.0-9.9 events/10,000 person-years) in the primary analysis after exclusion of initiators who were additionally dispensed any other Group 1 or 2 medications. Colors represent the categories of likelihood to cause severe acute liver injury based on numbers of published case reports of hepatotoxicity.

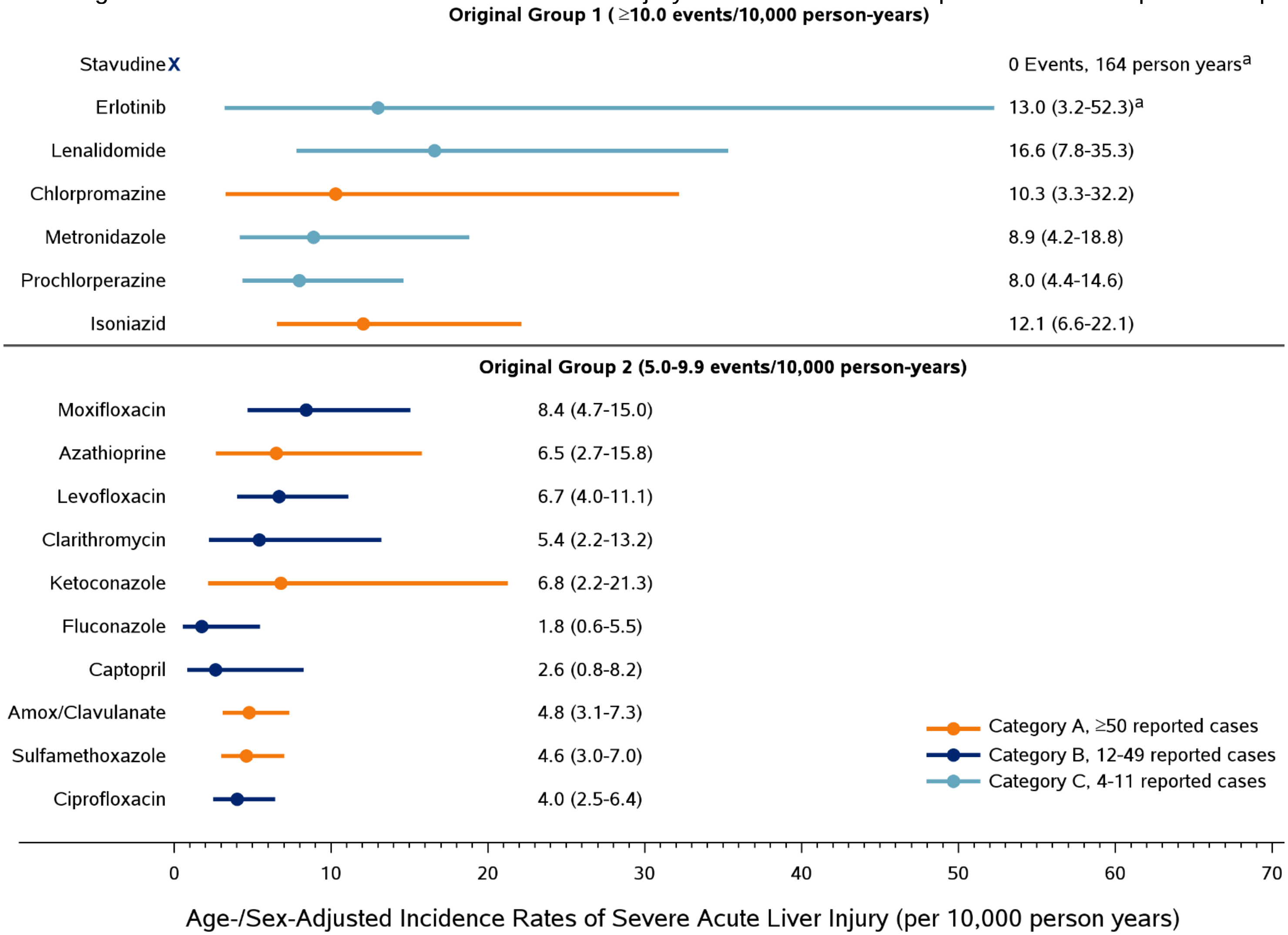

[a] After exclusion of initiators on multiple hepatotoxic medications, the 95% confidence interval of the incidence rate was too wide to meet our initial criteria for classification into Groups 1-5.

eFigure 8. Rates of severe acute liver injury for Group 3-4 initiators additionally dispensed Group 1-2 medications. Age-/sex-adjusted incidence rates of severe acute liver injury (per 10,000 person years and with 95% confidence intervals) are reported for medications classified in Group 3 (3.0-4.9 events/10,000 person-years) and Group 4 (1.0-2.9 events/10,000 person-years) in the primary analysis after exclusion of initiators who were additionally dispensed any Group 1 or 2 medications (as identified in the primary analysis). Colors represent the categories of likelihood to cause severe acute liver injury based on numbers of published case reports of hepatotoxicity.

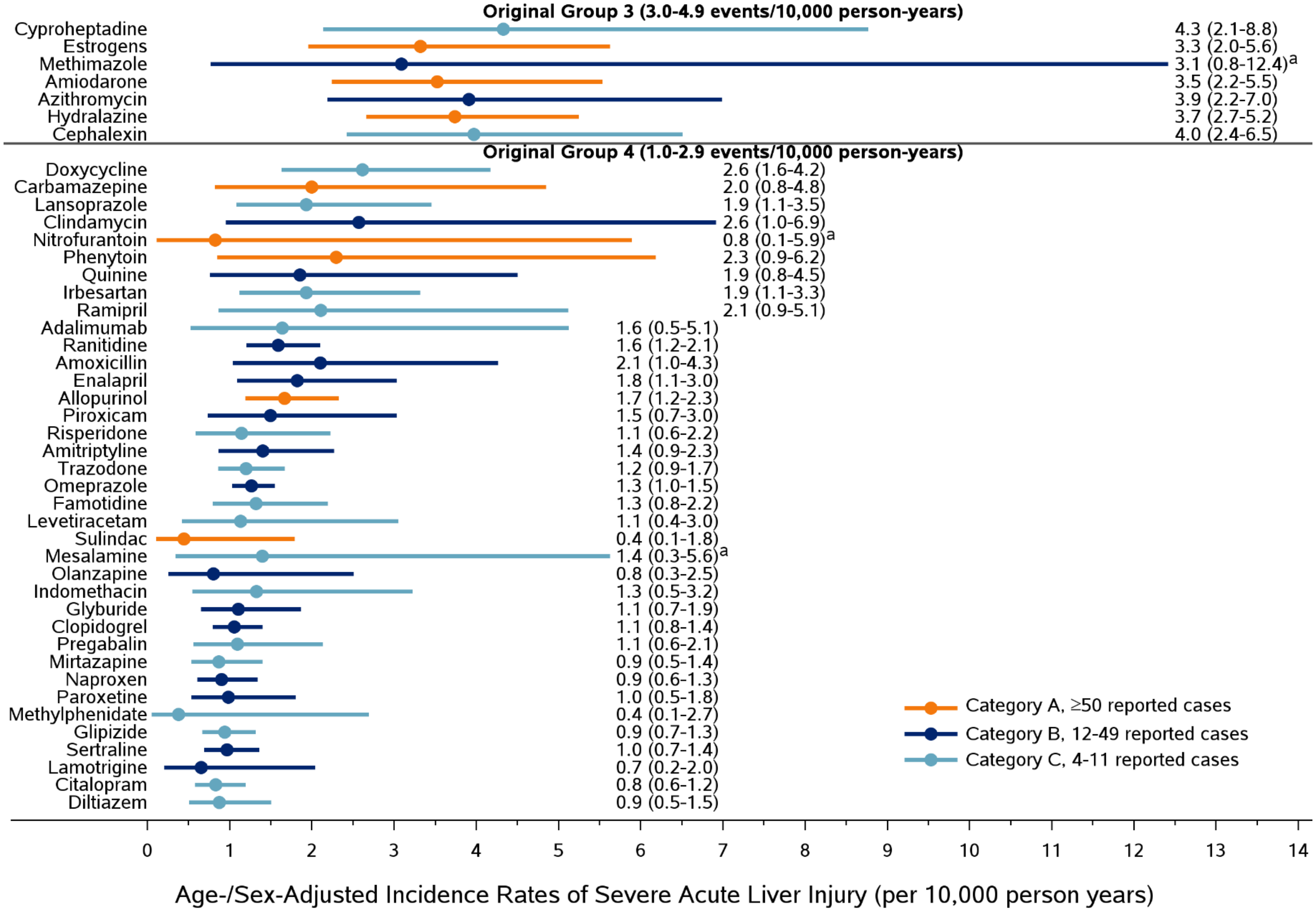

[a] After exclusion of initiators on multiple hepatotoxic medications, the 95% confidence interval of the incidence rate was too wide to meet our initial criteria for classification into Groups 1-5.

eFigure 9. Rates of severe acute liver injury for Group 5 initiators additionally dispensed Group 1-2 medications. Age-/sex-adjusted incidence rates of severe acute liver injury (per 10,000 person years and with 95% confidence intervals) are reported for medications classified in Group 5 (<1.0 events/10,000 person-years) in the primary analysis after exclusion of initiators who were additionally dispensed any Group 1 or 2 medications (as identified in the primary analysis). Colors represent the categories of likelihood to cause severe acute liver injury based on numbers of published case reports of hepatotoxicity.

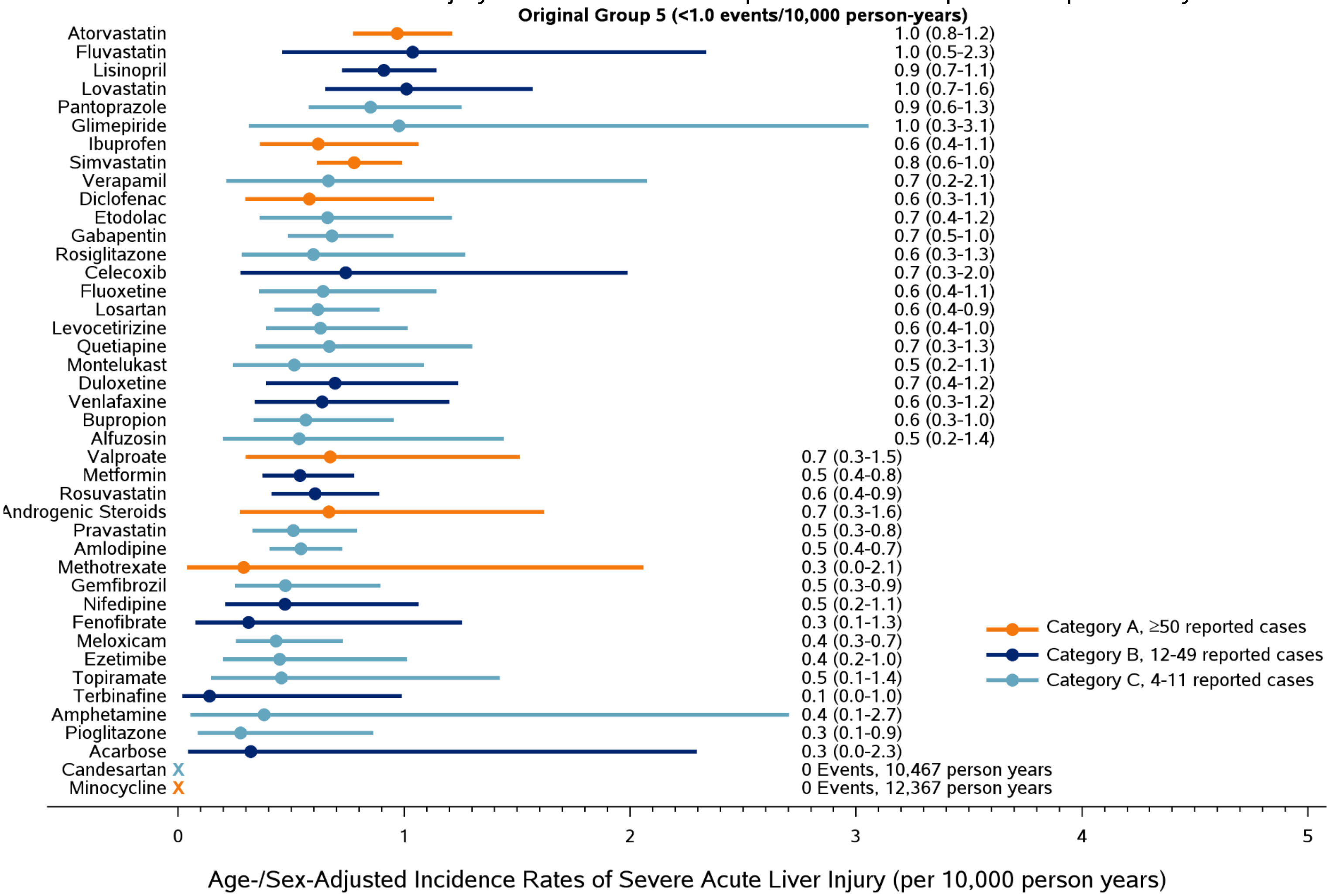

Figure 10. Rates of severe acute liver injury for Group 1-2 initiators dispensed ≥5 unique medications. Age-/sex-adjusted incidence rates of severe acute liver injury (per 10,000 person years and with 95% confidence intervals) are reported for medications classified in Group 1 (≥10.0 events/10,000 person-years) and Group 2 (5.0-9.9 events/10,000 person-years) in the primary analysis after limiting each medication initiator cohort to persons who were dispensed ≥5 unique medications on or within 90 days prior to the index date. Colors represent the categories of likelihood to cause severe acute liver injury based on numbers of published case reports of hepatotoxicity.

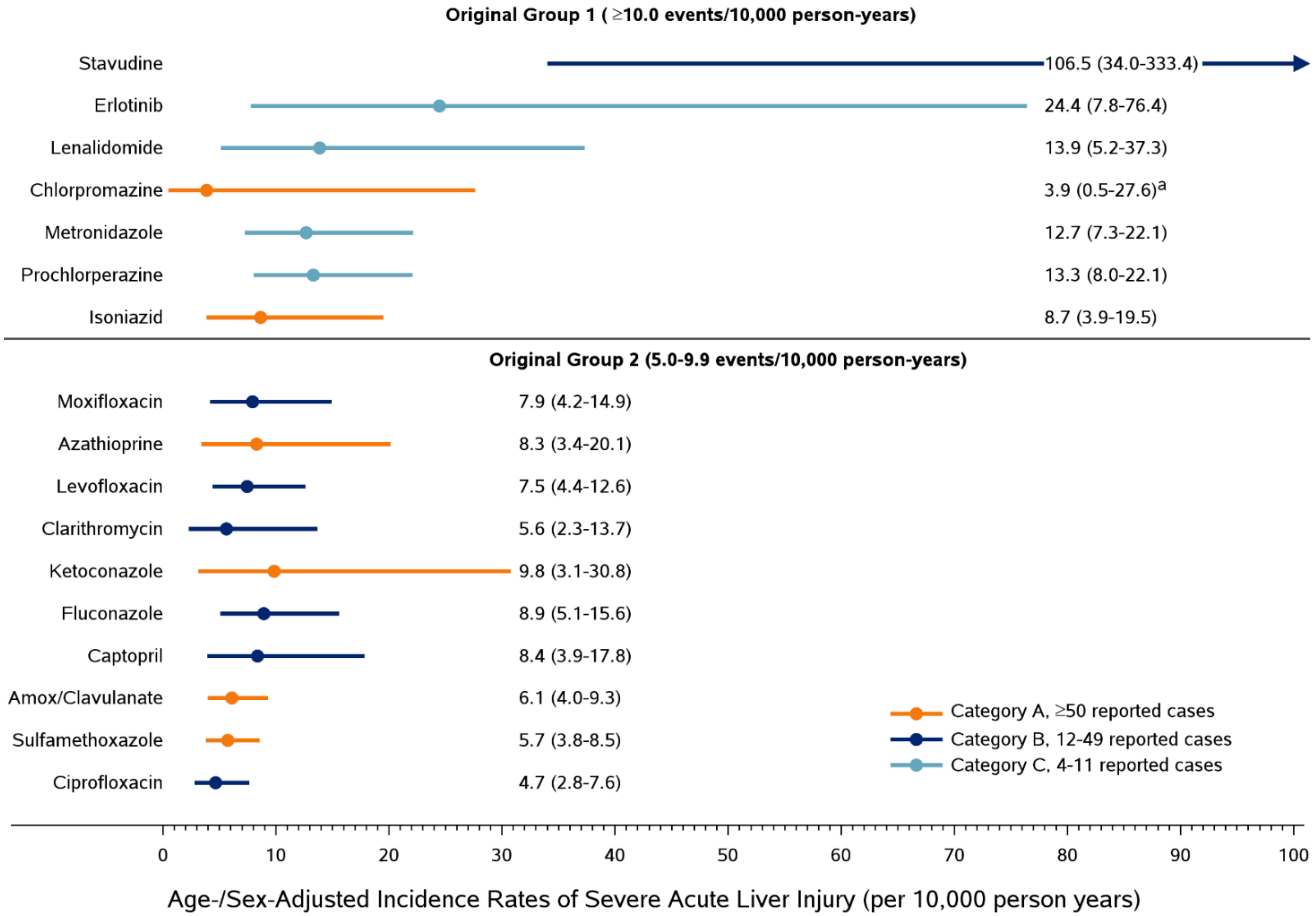

[a] After exclusion of initiators on multiple hepatotoxic medications, the 95% confidence interval of the incidence rate was too wide to meet our initial criteria for classification into Groups 1-5.

eFigure 11. Rates of severe acute liver injury for Group 3-4 initiators dispensed ≥5 unique medications. Age-/sex-adjusted incidence rates of severe acute liver injury (per 10,000 person years and with 95% confidence intervals) are reported for medications classified in Group 3 (3.0-4.9 events/10,000 person-years) and Group 4 (1.0-2.9 events/10,000 person-years) in the primary analysis after limiting each medication initiator cohort to persons who were dispensed ≥5 unique medications on or within 90 days prior to the index date. Colors represent the categories of likelihood to cause severe acute liver injury based on numbers of published case reports of hepatotoxicity.

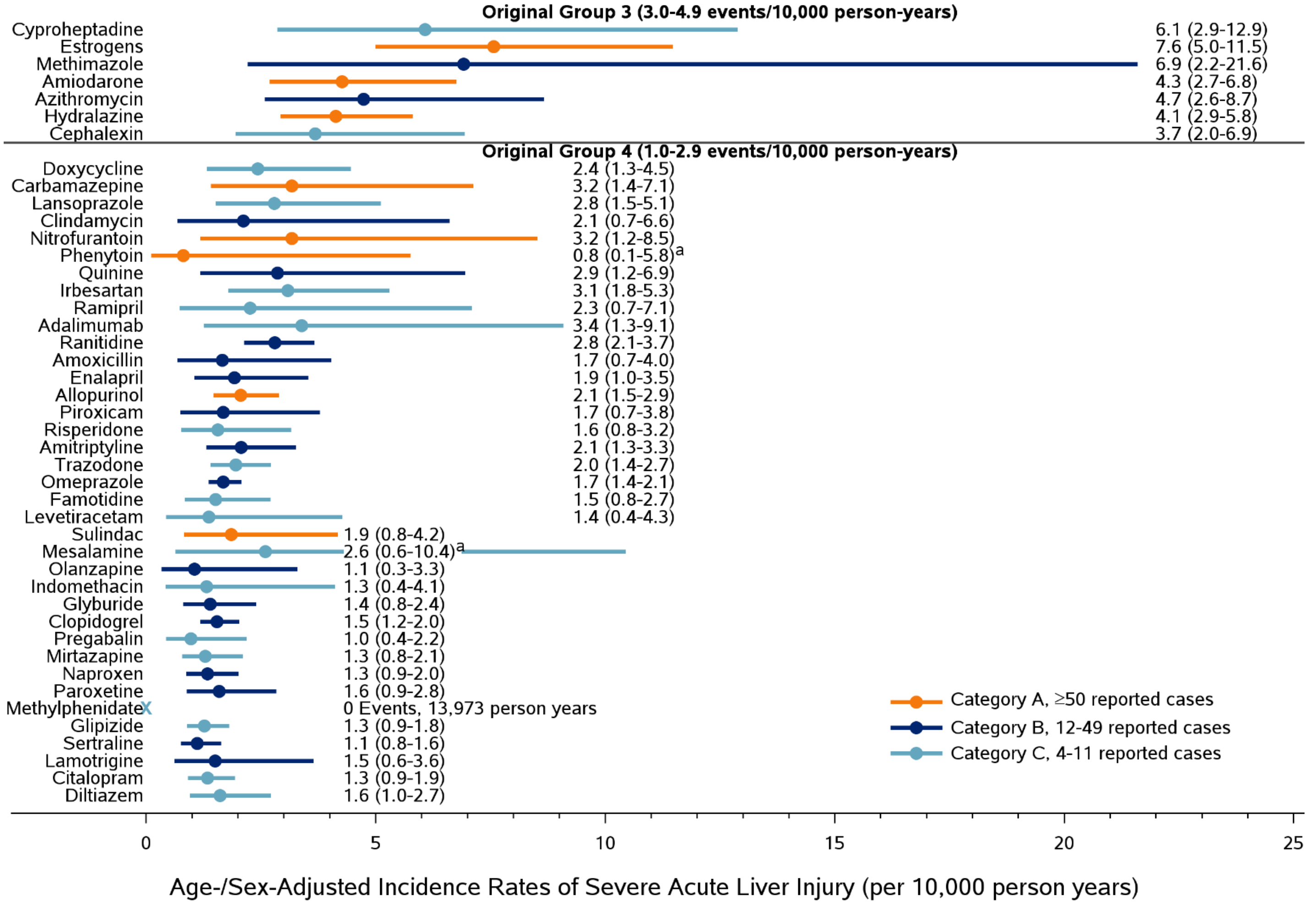

[a] After exclusion of initiators on multiple hepatotoxic medications, the 95% confidence interval of the incidence rate was too wide to meet our initial criteria for classification into Groups 1-5.

eFigure 12. Rates of severe acute liver injury for Group 5 initiators dispensed ≥5 unique medications. Age- and sex-adjusted incidence rates of severe acute liver injury (per 10,000 person years and with 95% confidence intervals) are reported for medications classified in Group 5 (<1.0 events/10,000 person-years) in the primary analysis after limiting each medication initiator cohort to persons who were dispensed ≥5 unique medications on or within 90 days prior to the index date. Colors represent the categories of likelihood to cause severe acute liver injury based on numbers of published case reports of hepatotoxicity.

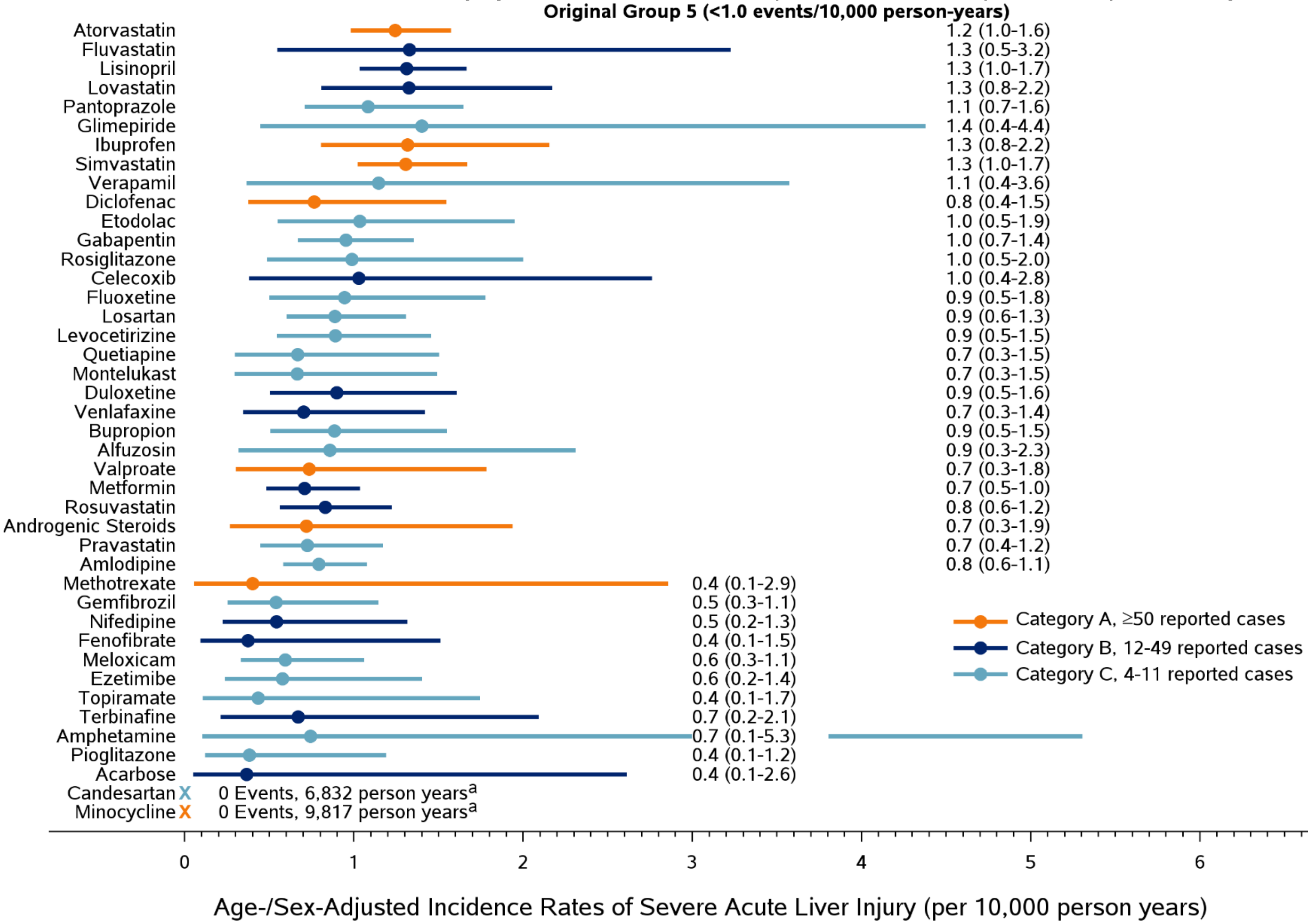

[a] After exclusion of initiators on multiple hepatotoxic medications, the 95% confidence interval of the incidence rate was too wide to meet our initial criteria for classification into Groups 1-5.
