# Supplementary material for: Severe Acute Liver Injury After Hepatotoxic Medication Initiation in Real-World Data
Source: JAMA Intern Med. 2024 Jun 24;184(8):943–52. doi: 10.1001/jamainternmed.2024.1836 (PMC11197444; doi:10.1001/jamainternmed.2024.1836)
Supplement: Supplement 2. — Data Sharing Statement [file jamainternmed-e241836-s002.pdf]

Data Sharing Statement

Torgersen. Severe Acute Liver Injury After Hepatotoxic Medication Initiation in Real-World Data. *JAMA Intern Med*. Published June 24, 2024. doi:10.1001/jamainternmed.2024.1836

Data
Data available: No

Additional Information
Explanation for why data not available: Due to US Department of Veterans Affairs (VA) regulations and our ethics agreements, the analytic data sets used for this study are not permitted to leave the VA without a Data Use Agreement. This limitation is consistent with other studies based on VA data. However, VA data are made freely available to researchers with an approved VA study protocol. For more information, please visit https://www.virec.research.va.gov or contact the VA Information Resource Center (VIReC) at VIReC@va.gov.
